# Reduced Complexity Model Intercomparison Project Phase 2: Synthesizing Earth System Knowledge for Probabilistic Climate Projections

**DOI:** 10.1029/2020EF001900

**Published:** 2021-06-04

**Authors:** Z. Nicholls, M. Meinshausen, J. Lewis, M. Rojas Corradi, K. Dorheim, T. Gasser, R. Gieseke, A. P. Hope, N. J. Leach, L. A. McBride, Y. Quilcaille, J. Rogelj, R. J. Salawitch, B. H. Samset, M. Sandstad, A. Shiklomanov, R. B. Skeie, C. J. Smith, S. J. Smith, X. Su, J. Tsutsui, B. Vega‐Westhoff, D. L. Woodard

**Affiliations:** ^1^ Australian‐German Climate & Energy College University of Melbourne Parkville VIC Australia; ^2^ School of Earth Sciences University of Melbourne Parkville VIC Australia; ^3^ Potsdam Institute for Climate Impact Research Member of the Leibniz Association Potsdam Germany; ^4^ Department of Geophysics University of Chile Santiago Chile; ^5^ Center for Climate and Resilience Research CR2 Santiago Chile; ^6^ Pacficic Northwest National Laboratory Richland WA USA; ^7^ International Institute for Applied Systems Analysis Laxenburg Austria; ^8^ Independent Researcher Potsdam Germany; ^9^ Department of Atmospheric and Oceanic Science University of Maryland‐College Park College Park USA; ^10^ Department of Physics Atmospheric, Oceanic, and Planetary Physics University of Oxford Oxford UK; ^11^ Department of Chemistry and Biochemistry University of Maryland‐College Park College Park MD USA; ^12^ Grantham Institute Imperial College London London UK; ^13^ Earth System Science Interdisciplinary Center University of Maryland‐College Park College Park MD USA; ^14^ CICERO Center for International Climate Research Oslo Norway; ^15^ NASA Goddard Space Flight Center Greenbelt MD USA; ^16^ Priestley International Centre for Climate University of Leeds Leeds UK; ^17^ Joint Global Change Research Institute Pacific Northwest National Laboratory College Park MD USA; ^18^ Research Institute for Global Change/Research Center for Environmental Modeling and Application/Earth System Model Development and Application Group Japan Agency for Marine‐Earth Science and Technology Yokohama Japan; ^19^ Environmental Science Research Laboratory Central Research Institute of Electric Power Industry Abiko Japan; ^20^ Department of Atmospheric Sciences University of Illinois at Urbana‐Champaign Urbana IL USA

**Keywords:** climate, model intercomparison, probabilistic projections, RCMIP, reduced complexity climate model

## Abstract

Over the last decades, climate science has evolved rapidly across multiple expert domains. Our best tools to capture state‐of‐the‐art knowledge in an internally self‐consistent modeling framework are the increasingly complex fully coupled Earth System Models (ESMs). However, computational limitations and the structural rigidity of ESMs mean that the full range of uncertainties across multiple domains are difficult to capture with ESMs alone. The tools of choice are instead more computationally efficient reduced complexity models (RCMs), which are structurally flexible and can span the response dynamics across a range of domain‐specific models and ESM experiments. Here we present Phase 2 of the Reduced Complexity Model Intercomparison Project (RCMIP Phase 2), the first comprehensive intercomparison of RCMs that are probabilistically calibrated with key benchmark ranges from specialized research communities. Unsurprisingly, but crucially, we find that models which have been constrained to reflect the key benchmarks better reflect the key benchmarks. Under the low‐emissions SSP1‐1.9 scenario, across the RCMs, median peak warming projections range from 1.3 to 1.7°C (relative to 1850–1900, using an observationally based historical warming estimate of 0.8°C between 1850–1900 and 1995–2014). Further developing methodologies to constrain these projection uncertainties seems paramount given the international community's goal to contain warming to below 1.5°C above preindustrial in the long‐term. Our findings suggest that users of RCMs should carefully evaluate their RCM, specifically its skill against key benchmarks and consider the need to include projections benchmarks either from ESM results or other assessments to reduce divergence in future projections.

## Key Points

- Probabilistic global‐mean temperature projections often use reduced complexity climate models (RCMs) because of their low computational cost
- We evaluate how well RCMs’ probabilistic setups can synthesize knowledge from multiple research domains for policy relevant projections
- No RCM is able to capture all forcing, warming, heat uptake and carbon cycle metrics we evaluate, however some come close across a range

## 1 Introduction

Coupled Earth System Models (ESMs) have evolved for decades as primary climate research tools (Kawamiya et al., 2020). They represent the state of the art of complex Earth system modeling. Nonetheless, they are not the tool of choice to assess the full breadth of scenario and Earth system response uncertainty that has been identified in the scientific literature. It is infeasible to assess the climate implications of hundreds to thousands of emissions scenarios with the world's most comprehensive ESMs, such as those participating in the Sixth Phase of the Couple Model Intercomparison Project (CMIP6) (Eyring et al., 2016), because of ESMs’ computational cost, the complexity in setting up input data and the sheer volume of output data generated. Yet, large scenario assessments are vital for understanding the consequences of various policy choices and their residual climate hazards.

Similarly, while some ESMs perform large, perturbed physics experiments (e.g., Murphy et al., 2014) that aim to explore a range of potential Earth system long‐term annual‐average responses, the ability to capture full uncertainty ranges is limited. The ability to capture full uncertainty ranges is limited because these ESMs are relatively rigid in their structure—lacking the ability to completely explore uncertainties in vital components like the carbon cycle or effective radiative forcings.

An answer to both of these challenges, i.e., (a) limited computational resources and (b) structural scope and flexibility to represent long‐term uncertainties in key metrics like global‐mean surface air temperatures, are Reduced Complexity Models (RCMs), often also referred to as simple climate models (SCMs). RCMs can play the vital role of extending the knowledge and uncertainties from multiple domains, particularly a multitude of ESM experiments, to probabilistic long‐term climate projections of key variables over a wide range of scenarios. Earth System Models of Intermediate Complexity (EMICs) may initially appear to be another option. However, due to the process‐based representations used by EMICs, their computational complexity and data requirements are still orders of magnitude greater than RCMs. As a result, even EMICs are not a feasible choice for the large‐scale, probabilistic assessment discussed here.

Typically, RCMs achieve computational efficiency and structural flexibility by limiting their spatial and temporal domains to global‐mean, annual‐mean quantities, i.e., the domains of relevance to long‐term, global climate change. In general, RCMs do not include representations of interannual variability, although the EMGC model (Table 1) is a clear exception to this rule. Rather than aiming to represent the physics of the climate system at the process level and high‐resolution, RCMs use parameterizations of the system which capture its large‐scale behavior at a greatly reduced computational cost. This allows them to perform 350 ‐year long simulations in a fraction of a second on a single CPU, multiple orders of magnitude faster than our most comprehensive ESMs which would take weeks to months on the world's most advanced supercomputers.

**Table 1:** Overview of the Models and Constraining Approaches Used in this Paper

Model	Constraining technique	Key references
CICERO‐SCM	591 members subsampled from a posterior of 30,040 members to form a set that match the proxy assessment ocean heat content distribution while excluding parameter sets with unrealistic aerosol ERF or unrealistic surface air temperature change from 1850–1900 to 1985–2014	Schlesinger et al. (1992), Joos et al. (1996), Etminan et al. (2016), Skeie et al. (2017, 2018), Z. R. J. Nicholls et al. (2020), and Skeie et al. (2021)
EMGC	160,000 sample members, retaining the 1,000 that minimize reduced‐chi‐squared between modeled and observed GMST and OHC from 1850 to 1999	Canty et al. (2013), Hope et al. (2017, 2020), and McBride et al. (2021)
FaIRv1.6.1	3,000 sample members retaining the 501 that minimize RMSE between modeled and observed 1850–2014 GMST	Millar et al. (2017) and Smith, Forster, et al. (2018)
FaIRv2.0.0‐alpha	1,000,000 member raw ensemble, constrained with likelihood of 2010–2019 level and rate of attributable warming, calculated using the Global Warming Index methodology (Haustein et al., 2017). 5000 members randomly drawn from the constrained ensemble for use here.	Millar et al. (2017), Haustein et al. (2017), Smith, Forster, et al. (2018), and Leach et al. (2020)
Hectorv2.5.0	10,000 member ensemble sampled from Markov chain Monte Carlo chains constrained with global surface temperature and ocean heat content	Vega‐Westhoff et al. (2019)
MAGICCv7.5.1	7,000,000 member Monte Carlo Markov Chain, 600 member subsample selected to match proxy assessed ranges	Meinshausen et al. (2009, 2011, 2020)
MCE v1.2	600 members sampled with a Metropolis‐Hastings algorithm through Bayesian updating to reflect an ensemble of complex climate models constrained with the proxy assessed ranges	Tsutsui (2017, 2020) (see also Joos et al. (1996) and Hooss et al. (2001))
OSCARv3.1	10,000 Monte Carlo members, weighted using their agreement with a set of assessed ranges (supplementary Text S1)	Gasser et al. (2017, (2018, 2020)
SCM4OPT v2.1	For each emission scenario, 2,000 sample members are used to reflect uncertainties resulting from carbon cycle, aerosol forcings and temperature change, while constrained by the historical mean surface temperature of HadCRUT.4.6.0.0 (Morice et al., 2012)	Su et al. (2017, 2018, 2020)

*Note*. Detailed descriptions of each model are available in supplementary Text S1.

A key example of large‐scale emissions scenario assessment, and the one we focus on in this paper, is the climate assessment of socioeconomic scenarios by Working Group 3 (WG3) of the Intergovernmental Panel on Climate Change (IPCC). Hundreds of emission scenarios were assessed in the IPCC’s Fifth Assessment Report (AR5, see Clarke et al. (2014)) as well as its more recent Special Report on Global Warming of 1.5°C (SR1.5, see Rogelj, Shindell, et al. (2018) and Huppmann et al. (2018)) (Scenario data is available at https://secure.iiasa.ac.at/web-apps/ene/AR5DB and https://data.ene.iiasa.ac.at/iamc-1.5c-explorer/ for AR5 and SR1.5 respectively, both databases are hosted by the IIASA Energy Program). For the IPCC's forthcoming Sixth Assessment (AR6), it is anticipated that the number of scenarios will be in the several hundreds to a thousand (an initial snapshot of scenarios based on the SSPs is available at https://tntcat.iiasa.ac.at/SspDb).

Running WG3‐type scenarios requires at least some representation of greenhouse gas cycles, atmospheric chemistry and dynamic vegetation modules. While some of the world's most comprehensive ESMs have the required components, they could not be used to sample scenario and parametric uncertainty for reasons of computational cost. The most comprehensive RCMs include parameterized representations of the required components (including feedbacks of climate on permafrost and wetland methane emissions), enabling the exploration of interacting uncertainties from multiple parts of the climate system in an internally consistent setup.

While RCMs do not include the detail of ESMs across the emissions‐climate change cause‐effect chain, they do tend to include uncertainty representations for more steps in the chain (i.e., RCMs tradeoff depth for breadth compared to ESMs). For example, many RCMs include the relationship between methane emissions and concentrations (including temperature and other feedbacks) whereas few ESMs do in their long‐term experiments. On the other hand, few RCMs directly use land‐cover information within their carbon cycles, and none consider it in the detailed way which ESMs do. In addition, there are clearly applications where RCMs are not a feasible tool. For example, near‐term attribution studies, such as the World Weather Attribution project (Uhe et al., 2016). For this latter application, large‐ensemble ESM runs are vital—as only they can reflect natural variability and weather patterns. Overall, there is no question that ESMs are by far the most important research tool to project future climate change. RCMs complement the ESM efforts. Within this paper, we focus on a very specific niche of this complementing role, i.e., the degree to which RCMs can synthesize multiple lines of evidence across the emissions‐climate change cause‐effect chain.

Typically, RCMs attempt to perform this synthesis using probabilistic parameter ensembles (see also Section 3), which are distinct from the emulator mode in which RCMs can also be run (see Nicholls et al., 2020 for a discussion of emulation with RCMs). These probabilistic parameter ensembles are derived based on knowledge of specific Earth system quantities drawn from multiple, often independent, research communities, e.g., historical global‐mean temperature increase, effective radiative forcing due to different anthropogenic emissions, ocean heat uptake, or cumulative land and ocean carbon uptake. The resulting distributions can then be used in a variety of applications, e.g., to assess the likelihood that different warming levels are reached under a specific emissions scenario (e.g., 50% and 66%) based on the combined available evidence. As a result of their probabilistic nature, the ensembles resulting from RCMs are conceptually different from an ensemble of multiple model outputs that has not been constructed with any relative probabilities in mind (such as those from CMIP6) taken without constraining or any other sort of postprocessing.

Within the IPCC, RCMs’ synthesizing niche facilitates the transfer of knowledge from Working Group I (WG1), which assesses the physical science of the climate system, to WG3, which assesses the socioeconomics of climate change mitigation. The goal of this knowledge transfer is consistency between WG3’s scenario classification and the physical science assessment of WG1—a key precondition to have confidence that WG3’s conclusions about the socioeconomic transformation required to mitigate anthropogenic climate change to specific levels are based on our latest scientific understanding. Here, we describe RCMs as “integrators of knowledge” because they integrate (a relevant subsection of) the assessment from WG1, providing WG3 with a tool that can be used for assessing the climate implications, particularly global‐mean temperature changes, of a wide range of emissions scenarios.

Due to their role in the IPCC assessment (and for analyzing mitigation options in line with temperature targets more generally), understanding the degree to which RCMs can reflect a range of independent radiative forcing, warming, heat uptake and concentration assessments simultaneously is of vital importance. Given that these assessments are independent, a single, internally consistent, model may not be able to capture them all. If RCMs are inherently biased in some way or they are unable to simultaneously capture the independent assessments, this will affect the WG3 climate assessment and interpretation of the RCMs’ outputs should be adjusted accordingly.

This study’s scope, in terms of number of climate dimensions considered and number of climate models evaluated, is unique. While there have been studies with single models which choose parameter sets that match various assessments of ECS and TCR (e.g., Meinshausen, Meinshausen, et al., 2009; Rogelj, Meinshausen, & Knutti, 2012) and Smith, Forster, et al. (2018) compared two models’ probabilistic outputs, no previous study into RCM probabilistic distributions is of the same breadth.

Here, in the second phase of RCMIP, we evaluate the degree to which multiple RCMs are able to synthesize Earth system knowledge within a probabilistic distribution. We then examine the implications of differences in these probabilistic distributions for climate projections. We extend previous probabilistic evaluation work and build on the progress made in the first phase of RCMIP (Z. R. J. Nicholls et al., 2020) and other RCM intercomparison studies (Harmsen et al., 2015; Schwarber et al., 2019; van Vuuren et al., 2011). We widen the first phase’s scope both in terms of number of climate dimensions considered and the number of models evaluated. To our knowledge, this is the most comprehensive evaluation performed to date of the ability of RCMs to capture a broad range of climate metrics and key indicators, such as those assessed by IPCC WG1.

## 2 Participating Models

Nine models have participated in RCMIP Phase 2 (Table 1 and supplementary Text S1). Models were invited to participate via an open invitation made available at rcmip.org and circulated via relevant researcher networks. All interested modeling teams were included. These models and their components range from simpler, regression‐based approaches to more complex representations with detailed processes and regions. The models have been constrained in a number of different ways, using statistical techniques ranging in complexity from Monte Carlo Markov Chains to using pass/fail criteria to determine valid parameter values. As a result, the models and techniques cover (to the best of our knowledge) the full range of techniques seen in the literature and their results allow us to evaluate the implications of different choices.

## 3 Methods

In this study, the RCMs are run in a probabilistic setup, also referred to as a probabilistic distribution. As discussed in Section 1, a probabilistic setup means that each RCM is run with an ensemble of parameter configurations. Specifically, for a given experiment, each RCM is run multiple times, each time in a different configuration, i.e., with different parameter values. All of these different runs are then combined to form a probabilistic set of outputs. With these probabilistic sets, we can then calculate ranges of each output variable of interest (e.g., global‐mean surface temperatures).

Modeling groups use a range of techniques to derive their parameter ensembles, i.e., to constrain their models (Table 1). In each probabilistic run, the parameter ensemble is fixed, i.e., the same set of parameter configurations will be used in each experiment. This choice ensures that the model outputs are deterministic, rather than including a random element due to, e.g., sampling parameter values from a range or probability distribution for each run. Typically, modeling groups will also use different data to derive their parameter ensemble. This can lead to differences in model projections which are simply based on choices made by the modeling groups and are not related to model structure or constraining technique at all. In this study, two models (MAGICC7 and MCE‐v1‐2) have used a common set of target assessed ranges, i.e., benchmarks, to derive their probabilistic distributions. For these models, we are able to rule out the choice of data as the cause of difference between these models. Accordingly, we can more clearly identify the importance of model structure and constraining technique for future projections.

In this study, our target assessment is a “proxy assessment”, which uses assessed climate system characteristics in line with IPCC AR5 as its starting point and updates key values using more recent literature (Table 2). We explicitly use the name “proxy assessment” throughout to make clear that we are not constraining to any ranges coming from the formal IPCC assessment, rather an approximation thereof. Notably, in this study, the proxy assessment does not include any future projections. While we examine future projections coming from the models, we do not explicitly compare them against future projections coming from another line of evidence because there is no obvious choice for such a line of evidence—apart from the “assessed ranges” of SSP scenarios that will be communicated in the forthcoming IPCC report (but are not available for this study). As discussed in more detail in Section 4.3, the inclusion of future projections in the proxy assessment would narrow the range of model projections but any such narrowing should be carefully considered because—depending on the types of constraints—it may lead to underestimates of uncertainty.

**Table 2:** The Proxy Assessed Ranges Used in this Study

Metric	Assessed range Unit	vll	ll	c	lu	vlu
2000–2019 GMST rel. to 1961–1990	K	0.46		0.54		0.61
Equilibrium climate sensitivity	K	2.30	2.60	3.10	3.90	4.70
Transient climate response	K	0.98	1.26	1.64	2.02	2.29
Transient climate response to emissions	K/TtC	1.03	1.40	1.77	2.14	2.51
2014 CO_2_ effective radiative forcing	W/m^2^		1.69	1.80	1.91	
2014 Aerosol effective radiative forcing	W/m^2^		−1.37	−1.01	−0.63	
2018 Ocean heat content rel. to 1971	ZJ		303	320	337	
2011 CH_4_ effective radiative forcing	W/m^2^		0.47	0.60	0.73	
2011 N_2_O effective radiative forcing	W/m^2^		0.14	0.17	0.20	
2011 F‐Gases effective radiative forcing	W/m^2^		0.03	0.03	0.03	

*Note*. The assessed ranges are labeled as “vll” (very likely lower, i.e., Fifth percentile), “ll” (likely lower, 17th percentile), “c” (central, 50th percentile), “lu” (likely upper, 83rd percentile), and “vlu” (very likely upper, 95th percentile). Sources are described in Section 3.

In order to keep the study’s scope manageable, our proxy assessment focuses on climate response parameters, with the carbon cycle examined only via the TCRE. We aim to perform a detailed analysis on carbon cycle response in the next phase of RCMIP.

We use surface air ocean blended temperatures from the HadCRUT.4.6.0.0 data set (Morice et al., 2012). HadCRUT4.6.0.0 is a widely used observational data product and is representative of other observations of changes in surface air and ocean temperatures (Simmons et al., 2017). Our key metric for evaluating RCM temperature projections is the warming between the 1961–1990 and 2000–2019 periods (using the SSP2‐4.5 scenario to extend the CMIP6 historical experiment to 2019). We choose a relatively recent period to match the increase in global observations since the 1960s.

For ocean heat content, we use the recent work of von Schuckmann et al. (2020). We focus on the change in ocean heat content between 1971 and 2018, when the largest set of observations are available.

We use the recent assessment of Sherwood et al. (2020) for equilibrium climate sensitivity (ECS). ECS is defined as the equilibrium warming which occurs under a doubling of atmospheric CO_2_ concentrations relative to preindustrial concentrations. The ECS assessment is combined with the constrained transient climate response (TCR) assessment of Tokarska et al. (2020). TCR is defined as the surface air temperature change which occurs at the time at which atmospheric CO_2_ concentrations double in an experiment in which atmospheric CO_2_ concentrations rise at one percent per year (a 1pctCO2 experiment). Carbon cycle behavior is considered only via the transient climate response to emissions (TCRE). TCRE is defined as the ratio of surface air temperature change to cumulative CO_2_ emissions at the time when atmospheric CO_2_ concentrations double in a 1pctCO2 experiment. We use the TCRE assessment from Arora, Katavouta, et al. (2020), which is based on the latest generation of Earth System Models which have participated in CMIP6 (Eyring et al., 2016). There is a potential inconsistency between our ECS, TCR and TCRE ranges, which arises because the ECS assessment comes from a study which uses multiple lines of evidence, the TCR assessment is based on a constrained set of CMIP6 models and the TCRE assessment is based on unconstrained CMIP6 Earth System Models. We discuss the importance of this inconsistency and its consequences in Section 4.

The other key metrics are related to effective radiative forcing (ERF, Forster et al., 2016). These values generally follow the AR5 assessment, except for aerosol, CO_2_, and methane ERF. For aerosol and CO_2_ ERF, we use the more recent work of Smith, Kramer, Myhre, Alterskjær, et al. (2020). For methane ERF, we increase the AR5 assessment following Etminan et al. (2016) although we note that this increase may be offset by an updated understanding of the impact of rapid adjustments (Smith, Kramer, et al., 2018).

At this point, we stress that our proxy assessed ranges are only one of a range of possible choices. Assessing all the available literature is a demanding task that is well undertaken by the IPCC. We do not attempt to reproduce this task here. Instead, the key is that our proxy assessed ranges are (a) reasonable and (b) were available at the time of the study's inception.

Following this intercomparison consortium's choice of proxy assessed ranges, modeling groups then had the opportunity to develop parameter ensembles which best reflected these assessed ranges. As previously discussed, this allowed some modeling teams (although crucially not all) to use the same “constraining benchmarks” (with a number of different techniques being employed to consider the constraining benchmarks, see Table 1). We use these consistently constrained models to gain unique insights into the impact of differences in model structure and constraining techniques when RCMs are used as integrators of knowledge, free from a typical source of disagreement between the models, namely that they were constrained to reproduce different understandings of the climate. The inclusion of results from models which were not constrained using the same benchmarks allows us to quantify the importance of constraining when using reduced complexity climate models as integrators of knowledge.

The modeling groups submitted a range of concentration‐driven, emission‐driven and idealized scenarios for their chosen parameter subsets (see scenario specifics below). Subsequently, several metrics were calculated, such as TCR from the idealized CO_2_‐only 1pctCO2 experiment (in which atmospheric CO_2_ concentrations rise at 1% per year from preindustrial levels). Calculating derived metrics on each individual ensemble member ensures that all metrics are calculated from internally self‐consistent model runs, which is of particular importance when the metric is based on more than one output variable from the model (e.g., TCRE, which relies on both surface air temperature change and inverse emissions of CO_2_). If we instead calculated results based on percentiles of different variables, we would not be using an internally self‐consistent set. Where modeling groups felt it was more appropriate (e.g., OSCARv3.1), they performed their own weighting of ensemble members before submitting.

The one metric which is not easily calculated from model results is ECS because it is defined at equilibrium. Accordingly, modeling groups reported their own diagnosed ECS for each ensemble member, rather than performing experiments which would allow it to be calculated after submission had taken place.

When evaluating model performance, we are interested not only in how well a model can reproduce the best‐estimate, but also the range, of a given quantity. A key part of any climate assessment is the uncertainty and it is critical that RCMs reflect the assessed likely and very likely ranges if they are to be used as integrators of knowledge. We assess the relative difference between the model and the assessed ranges at the very likely lower (fifth percentile, also referred to as “vll”), likely lower (17th percentile, “ll”), central (50th percentile, “c”), likely upper (83rd percentile, “lu”), and very likely upper (95th percentile, “vlu”). Assessing deviations using relative differences allows us to quickly evaluate how models perform over a range of metrics on the same scale.

The set of scenarios that each modeling group was asked to run follow the experimental protocols of CMIP6’s ScenarioMIP (O’Neill et al., 2016). The SSPX‐Y.Y experiments (e.g., SSP1‐1.9, SSP2‐4.5, SSP5‐8.5) are defined in terms of concentrations of well‐mixed greenhouse gases, i.e., CO_2_, CH_4_, N_2_O, hydrofluorocarbons (HFCs), perfluorocarbons (PFCs), and hydrochlorofluorocarbons (HCFCs), emissions of “aerosol precursor species emissions”, i.e., sulfur, nitrates, black carbon, organic carbon and ammonia and natural effective radiative forcing variations. As described in Z. R. J. Nicholls et al. (2020), where required, models may use prescribed effective radiative forcing if they do not include the required gas cycles or radiative forcing parameterizations.

The esm‐SSPX‐Y.Y experiments are identical to the SSPX‐Y.Y experiments, except CO_2_ emissions are prescribed instead of CO_2_ concentrations, following the CMIP6 C4MIP protocol (Jones et al., 2016). Finally, we also perform esm‐SSPX‐Y.Y‐allGHG experiments. These are identical to the esm‐SSPX‐Y.Y experiments, except they are defined in terms of emissions of all well‐mixed greenhouse gases, not only CO_2_, rather than concentrations. There is no equivalent of these esm‐SSPX‐Y.Y‐allGHG experiments in the CMIP6 protocol, however it is these experiments which are of most interest to WG3, given that WG3 focuses on scenarios defined in terms of emissions alone. We use the data sources described in Z. R. J. Nicholls et al. (2020) to specify the inputs for each of these scenarios. The input data set compilations, comprising emission, scenario and forcing data, as well as the protocols are archived with Zenodo (Z. Nicholls & Lewis, 2021)—and can contribute to scientific studies beyond this intercomparison as they largely reflect the CMIP6 experimental designs.

The protocol designed for this study requires that each RCM modeling group runs every probabilistic ensemble member once for each scenario and then submits their output for further analysis. With nine modeling groups participating, this intercomparison project compiled a database of results containing thousands of runs for each RCM, from which we can calculate different warming, effective radiative forcing or ocean heat uptake percentiles for a wide range of scenarios.

## 4 Results and Discussion

### 4.1 Fit to Assessed Ranges

**Figure 1:**
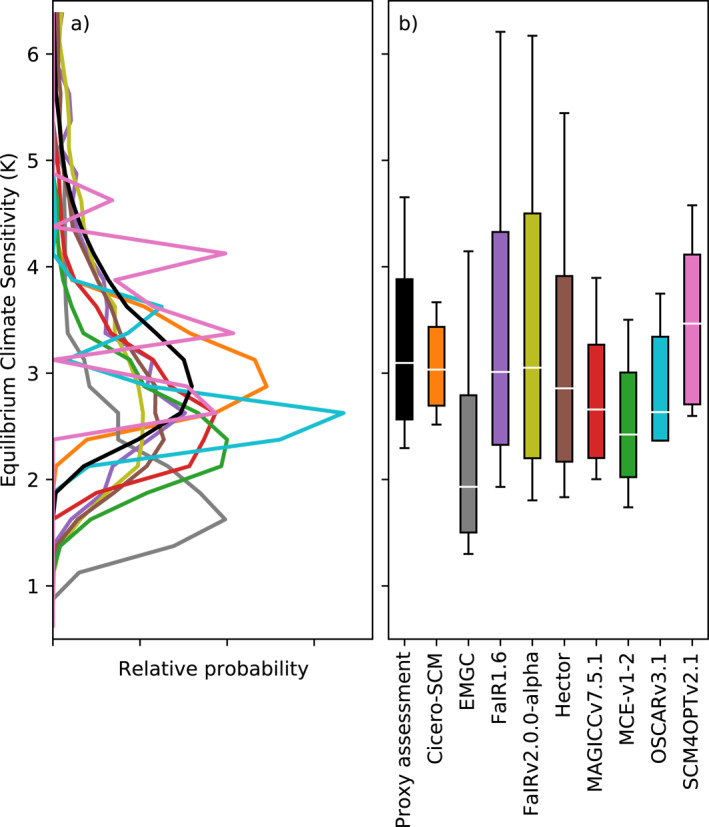
Distribution of equilibrium climate sensitivity (ECS) from each RCM (colored lines) and the proxy assessed range (solid black line). (a) Distribution of ECS; (b) very likely (whiskers), likely (box), and central (white solid line) from the proxy assessment and each RCM.

The ability of RCMs to match the assessed ranges varies (Table 3, Figure 1, Table S1, and Figures S1–S9). In general, the RCMs capture the central assessed values better than the likely and very likely ranges. Historical warming, TCR and the TCRE are notable exceptions to this. For the TCR, the upper likely and very likely upper assessed values are captured by the RCMs about as well as the central value. For TCRE and historical warming, the very likely lower and likely lower assessed values are better captured by the RCMs than the central values.

**Table 3:** Comparison of Each Model's Probabilistic Distribution With the Proxy Assessment

Climate model Assessed range	Multimodel median of magnitude of relative differences				
	vll	ll	c	lu	vlu
2000–2019 GMST rel. to 1961–1990	7%		11%		25%
Equilibrium climate sensitivity	**16%**	**15%**	**12%**	**14%**	**20%**
Transient climate response	38%	18%	7%	4%	7%
Transient climate response to emissions	**9%**	**11%**	**20%**	**19%**	**20%**
2014 CO_2_ effective radiative forcing		**5%**	**5%**	**1%**	
2014 Aerosol effective radiative forcing		**14%**	**10%**	**19%**	
2018 Ocean heat content rel. to 1971		**1%**	**1%**	**16%**	
2011 CH_4_ Effective radiative forcing		**4%**	**6%**	**18%**	
2011 N_2_O Effective radiative forcing		**11%**	**2%**	**10%**	
2011 F‐Gases effective radiative forcing		**2%**	**3%**	**4%**	

Climate model Assessed range	CICERO‐SCM					EMGC					FaIR1.6				
	vll	ll	c	lu	vlu	vll	ll	c	lu	vlu	vll	ll	c	lu	vlu
2000–2019 GMST rel. to 1961–1990	**6%**		**12%**		**17%**	−30%		−11%		35%	14%		23%		37%
Equilibrium climate sensitivity	9%	4%	−2%	−12%	−22%	−43%	−42%	−38%	−28%	−12%	−16%	−11%	−3%	11%	32%
Transient climate response	33%	14%	1%	−6%	−12%						43%	23%	10%	5%	8%
Transient climate response to emissions											**17%**	**−3%**	**−10%**	**−11%**	**−12%**
2014 CO_2_ effective radiative forcing		**15%**	**8%**	**2%**			**12%**	**6%**	**−0%**			**−2%**	**5%**	**14%**	
2014 Aerosol effective radiative forcing		28%	37%	64%			**16%**	**16%**	**16%**			**2%**	**0%**	**−4%**	
2018 Ocean heat content rel. to 1971		**0%**	**−0%**	**−0%**			−9%	2%	26%			−0%	12%	24%	
2011 CH_4_ effective radiative forcing		12%	−12%	−27%			23%	−3%	−20%			**3%**	**−8%**	**−15%**	
2011 N_2_O effective radiative forcing		**13%**	**−6%**	**−20%**			25%	4%	−12%			**8%**	**−2%**	**−9%**	
2011 F‐Gases Effective Radiative Forcing												**−1%**	**−3%**	**−4%**	

Climate model Assessed range	FaIRv2.0.0‐alpha					Hector					MAGICCv7.5.1				
	vll	ll	c	lu	vlu	vll	ll	c	lu	vlu	vll	ll	c	lu	vlu
2000–2019 GMST rel. to 1961–1990	11%		22%		38%	7%		16%		25%	**1%**		**1%**		**2%**
Equilibrium climate sensitivity	−22%	−15%	−2%	15%	31%	**−20%**	**−17%**	**−8%**	**0%**	**16%**	**−13%**	**−15%**	**−14%**	**−16%**	**−17%**
Transient climate response	26%	12%	4%	3%	4%	45%	25%	11%	3%	0%	32%	16%	7%	2%	0%
Transient climate response to emissions	**−1%**	**−16%**	**−20%**	**−19%**	**−20%**						**9%**	**−2%**	**−1%**	**1%**	**−2%**
2014 CO_2_ effective radiative forcing		**3%**	**8%**	**14%**								**−2%**	**−1%**	**−1%**	
2014 Aerosol effective radiative forcing		−12%	−13%	−27%			51%	44%	29%			**−1%**	**−7%**	**−19%**	
2018 Ocean heat content rel. to 1971												**1%**	**1%**	**1%**	
2011 CH_4_ effective radiative forcing		**5%**	**−1%**	**−5%**								**−1%**	**−3%**	**−3%**	
2011 N_2_O Effective radiative forcing		**4%**	**−2%**	**−7%**								**1%**	**2%**	**2%**	
2011 F‐Gases effective radiative forcing		**3%**	**5%**	**7%**								**1%**	**1%**	**1%**	

Climate model Assessed range	MCE‐v1‐2					OSCARv3.1					SCM4OPTv2.1				
	vll	ll	c	lu	vlu	vll	ll	c	lu	vlu	vll	ll	c	lu	vlu
2000–2019 GMST rel. to 1961–1990	**−1%**		**3%**		**3%**	**3%**		**2%**		**0%**	−8%		10%		28%
Equilibrium climate sensitivity	−24%	−22%	−22%	−23%	−26%	**3%**	**−9%**	**−15%**	**−14%**	**−20%**	**13%**	**4%**	**12%**	**6%**	**−3%**
Transient climate response	23%	4%	−6%	−12%	−15%	44%	21%	−1%	−10%	−15%	61%	35%	9%	0%	−6%
Transient climate response to emissions	0%	−18%	−23%	−23%	−27%	11%	−11%	−21%	−24%	−28%					
2014 CO_2_ Effective radiative forcing		**−1%**	**−0%**	**1%**			**13%**	**6%**	**0%**			**6%**	**−0%**	**−6%**	
2014 Aerosol effective radiative forcing		**10%**	**6%**	**−9%**			−21%	−10%	−1%			−14%	−10%	−23%	
2018 Ocean heat content rel. to 1971		**−1%**	**−0%**	**1%**			−20%	5%	28%			−21%	0%	16%	
2011 CH_4_ Effective radiative forcing		**−1%**	**−3%**	**−4%**			5%	−17%	−32%			2%	−19%	−34%	
2011 N_2_O Effective radiative forcing		**−1%**	**−0%**	**−1%**			26%	5%	−11%			21%	0%	−14%	
2011 F‐Gases effective radiative forcing		**0%**	**−0%**	**0%**			**15%**	**4%**	**−6%**			27%	14%	4%	

*Note*. In each square, we show the relative difference between the model result and the proxy assessed value (Δ_*m*_, calculated as $\Delta_{m}=\frac{m-a}{\left|a\right|}$ where *m* is the value from the model’s probabilistic distribution and *a* is the proxy assessment value). Bold cells indicate that this model is within 20% of the proxy assessment at all likelihood levels for this metric. If a row is completely empty for a model, this indicates that the model did not submit results which allowed that metric to be calculated. Empty cells within a row which is otherwise not completely empty for a model indicates that no proxy assessment at this likelihood level was available (e.g., we have proxy assessments for likely lower 2014 CO_2_ effective radiative forcing, but not for very likely lower 2014 CO_2_ effective radiative forcing). Only the magnitude of Δ_*m*_ from each model was used to calculate the multimodel median (to ensure that positive and negative values of Δ_*m*_ from different models would not cancel out). The assessed ranges are labeled as “vll” (very likely lower, i.e., fifth percentile), “ll” (likely lower, 17th percentile), “c” (central, 50th Percentile), “lu” (likely upper, 83rd percentile) and “vlu” (very likely upper, 95th percentile).

Considering the variation between metrics, we see that the proxy assessment of the ocean heat content and effective radiative forcing metrics is better captured by the RCMs than the other metrics. For the ocean heat content and effective radiative forcing metrics, the median multimodel difference is less than or equal to 10% for the central proxy assessed range. However, there is less close agreement with the very likely and likely proxy assessed ranges for the effective radiative forcing metrics, with median multimodel differences being up to 19% (aerosol effective radiative forcing).

For the other metrics (historical warming, ECS, TCR, and TCRE), the median multimodel difference is greater than 20% for at least one of the assessed ranges. However, there is significant variation across the likelihood levels. For example, the multimodel median matches the very likely lower historical warming (rows labeled “2000–2019 GMST rel. to 1961–1990” in Table 3) to within 7%. However, the multimodel median differs from the central and very likely upper historical warming by 11% and 25%, indicating that the models are having greater difficulty capturing the upper‐end warming estimates.

There is also significant spread in performance across the models. MAGICCv7.5.1 performs better than the multimodel median across all metrics and assessed ranges (very likely lower, likely lower, central, likely upper, very likely upper) except for ECS while MCE‐v1‐2 performs better than the multimodel median across all metrics and assessed ranges except for three metrics (ECS, TCR and TCRE). However, all RCMs had at least one metric where they matched the proxy assessment at all likelihood levels to within 20% (bold cells in Table 3). For many applications, agreement to within 20% will be sufficient given the uncertainty associated with assessed ranges. However, for some applications, using an RCM’s probabilistic distribution which has differences greater than 5–10% (for certain metrics) may be problematic as such differences could bias projections to an unacceptably large degree. For example, the WG3 classification of scenarios in terms of their peak warming levels should ideally be consistent with the range of evidence assessed in IPCC WG1. To have confidence that such an application is reflecting the WG1 assessment, the RCMs should be within 5–10% of the assessed results (particularly for any future warming assessment).

When interpreting these results it is vital to keep in mind that, for some models, the same benchmarks are used to both constrain and evaluate the models. The reason for this choice is that we are evaluating the ability of the models to act as integrators of knowledge, i.e., to simultaneously capture all the independent assessments (see also discussion in Section 1). We are not attempting to do a calibration followed by an out‐of‐sample evaluation.

As a result, it is not so surprising that the models which calibrated to the benchmarks, specifically MAGICC7 and MCE‐v1‐2, better reflect the benchmarks during the evaluation phase. However, the results presented here highlight just how important it is to calibrate if the model is to be used as an integrator of knowledge. If the goal is an integrator of knowledge which reflects key benchmarks, our results suggest that models which are calibrated will perform better.

### 4.2 Projection Results

For each probabilistic setup, the RCMs also submitted projections of global‐mean surface temperature, effective radiative forcing (split into total, aerosols and CO_2_) and atmospheric CO_2_ concentrations for the SSPX‐Y.Y, ESM‐SSPX‐Y.Y, and ESM‐SSPX‐Y.Y‐allGHG experiments.

#### 4.2.1 Global‐Mean Surface Air Temperature

**Figure 2:**
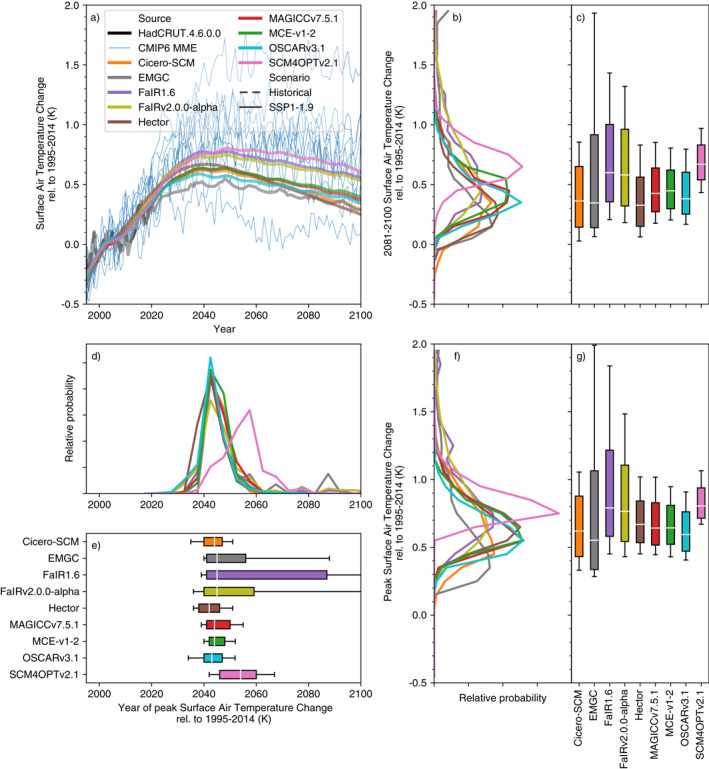
Surface air temperature (also referred to as global‐mean surface air temperature, GSAT) change under the very‐low‐emissions SSP1‐1.9 scenario. (a) GSAT projections from 1995 to 2100. We show the median RCM projections (colored lines), GMST observations from HadCRUT4.6.0.0 (Morice, Kennedy, Rayner, & Jones, 2012) up to 2019 (dashed black line) and CMIP6 model projections (thin blue lines, we show a single ensemble member for each CMIP6 model to preserve the CMIP6 models’ natural variability signal); (b) distribution of 2081–2100 mean GSAT from each RCM; (c) very likely (whiskers), likely (box) and central (white line) 2081–2100 mean GSAT estimate from each RCM; (d) as in (b) except for the year in which GSAT peaks; (e) as in (c) except for the year in which GSAT peaks; (f) as in (b) except for the peak GSAT; (g) as in (c) except for the peak GSAT. All results are shown relative to the 1995–2014 reference period.

Under SSP1‐1.9, median end of century (2081–2100) projections relative to 1995–2014 vary by 0.4°C across the models (from Hector with 0.3°C of warming to SCM4OPTv2.1 with 0.7°C, Figures 2a–2c). Variations in fifth percentile warming show a similar range, from 0.0 to 0.4°C. In contrast, upper‐end, 95th percentile warming shows far greater variation, from 0.8 to 1.9°C. For the SSP1‐1.9 scenario, the spread in RCMs’ probabilistic projections is similar to the spread in the CMIP6 multimodel ensemble. Nonetheless, the most extreme CMIP6 model projections are outside the range of most RCMs’ 5–95th percentiles. We discuss reasons for this difference in Section 4.3.

A slightly smaller spread is seen in peak temperature (Figures 2f and 2g). Across the RCM ensemble, SSP1‐1.9 median peak warming ranges from 0.55 to 0.8°C while the 5th and 95th percentiles range from 0.3 to 0.7°C and 0.9 to 2.0°C, respectively. The year of peak warming shows much more variation, particularly at the upper end (Figures 2d and 2e). While the median peak year is fairly consistent across the RCMs’ ensembles, around 2045 (although SCM4OPTv2.1’s 2055 peak is a clear outlier), and the fifth percentile peak year varies from 2030 to 2040, the 95th percentile varies from 2050 to beyond the end of this century. In the EMGC, FaIR1.6 and FaIRv2.0.0‐alpha probabilistic distributions, there is a significant area of parameter space which results in ongoing warming even after CO_2_ emissions have reached net zero. These models also drive the spread in end of century temperature projections, particularly in the 95th percentile (Figures 2b and 2c).

In the SSP1‐2.6 scenario (Supplementary Figure S10), median peak warming ranges from 0.65 to 1.1°C (0.1–0.3°C higher than in SSP1‐1.9). Median end of century warming (relative to 1995–2014) ranges from 0.6 to 1.0°C. End of century fifth percentile warming ranges from 0.2 to 0.8°C and 95th percentile warming ranges from 1.2 to 2.0°C. As in SSP1‐1.9, a number of CMIP6 model projections lie above the upper end of the constrained RCMs.

Under SSP1‐2.6, the RCMs diverge more in their peak temperature projections, both compared to end of century warming and compared to SSP1‐1.9. Once again, the fifth percentile and median are fairly consistent (ranging from 0.3 to 0.9°C and 0.65 to 1.1°C, respectively). However, 95th percentile projections vary from 1.2 to 2.8°C. The divergence in upper‐end warming between SSP1‐2.6 and SSP1‐1.9 is driven by FaIR1.6, and appears to be the result of persistent warming after CO_2_ emissions reach net zero given that its 83rd percentile peak warming year is after 2100. Across the models, peak warming year shows a similar range to SSP1‐1.9, albeit occurring 25–30 years later in the median (ranging from 2065 to 2075). Once again, the fifth percentile (ranging from 2050 to 2060) shows a much smaller spread across the models than the 95th percentile (ranging from 2075 to beyond the end of the 21st Century).

The warmest RCMs in mitigation scenarios are also the warmest under the high‐emissions, SSP5‐8.5, scenario (Supplementary Figure S11). The exceptions to this are MAGICC7, which is one of the warmest models in SSP5‐8.5 even though it was around the median in mitigation scenarios, and SCM4OPTv2.1, which was the warmest model in mitigation scenarios but is slightly cooler than the warmest models in SSP5‐8.5. Under SSP5‐8.5, median end of century warming ranges from 2.5 to 3.6°C across the RCMs. Unlike the mitigation scenarios, there is a similar level of disagreement in 5th and 95th percentile warming, with the fifth percentile ranging from 1.7 to 3.1°C and the 95th percentile ranging from 3.8 to 5.4°C. The RCMs all make future warming projections in the lower‐half of the CMIP6 multimodel ensemble. Such a difference is largely explained by the constraints applied to the RCMs (see discussion in Section 4.3).

**Figure 3:**
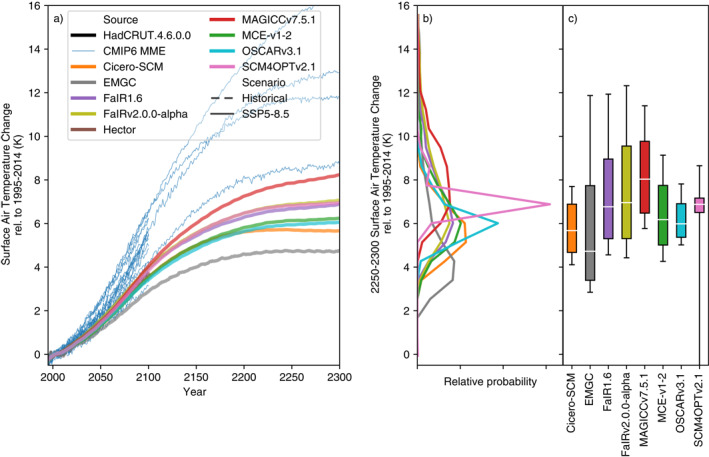
Long‐term surface air temperature (also referred to as global‐mean surface air temperature, GSAT) change under the high‐emissions SSP5‐8.5 scenario. (a) GSAT projections from 1995 to 2300. We show the median RCM projections (colored lines), GMST observations from (Morice, Kennedy, Rayner, & Jones, 2012) up to 2019 (dashed black line) and available CMIP6 model projections (thin blue lines, we show a single ensemble member for each CMIP6 model to preserve the CMIP6 models’ natural variability signal); (b) distribution of 2250–2300 mean GSAT from each RCM; (c) very likely (whiskers), likely (box), and central (white line) 2250–2300 mean GSAT estimate from each RCM. All results are shown relative to the 1995–2014 reference period.

If we consider long‐term (2250–2300) warming under the SSP5‐8.5 scenario (Figure 3, see Supplementary Figures S12 and S13 for long‐term warming under SSP1‐1.9 and SSP1‐2.6, respectively), the difference between RCMs and CMIP6 is even clearer (although the few CMIP6 models which have run the SSP5‐8.5 extension are all at or above the median of the CMIP6 multimodel ensemble in 2100). On these timescales, MAGICC7 is clearly the warmest model, despite having slightly lower long‐term effective radiative forcing than FaIR1.6, FaIR‐v2.0.0‐alpha, and MCE‐v1‐2 (Supplementary Figure S14). There is a significant spread in long‐term projections across the RCMs, with the median ranging from 4.5 to 8.0°C, fifth percentile from 3°C (ignoring SCM4OPTv2.1 as an outlier) to 5.8°C and 95th from 7.8 to 12.3°C. Even these upper end projections are well below the highest CMIP6 projections, which reach over 16°C of global‐mean warming (again, likely due to constraining, see discussion in Section 4.3). Across all the RCMs, only CICERO‐SCM shows any sign of temperatures peaking by 2300 under such a high‐emissions scenario.

#### 4.2.2 Effective Radiative Forcing

**Figure 4:**
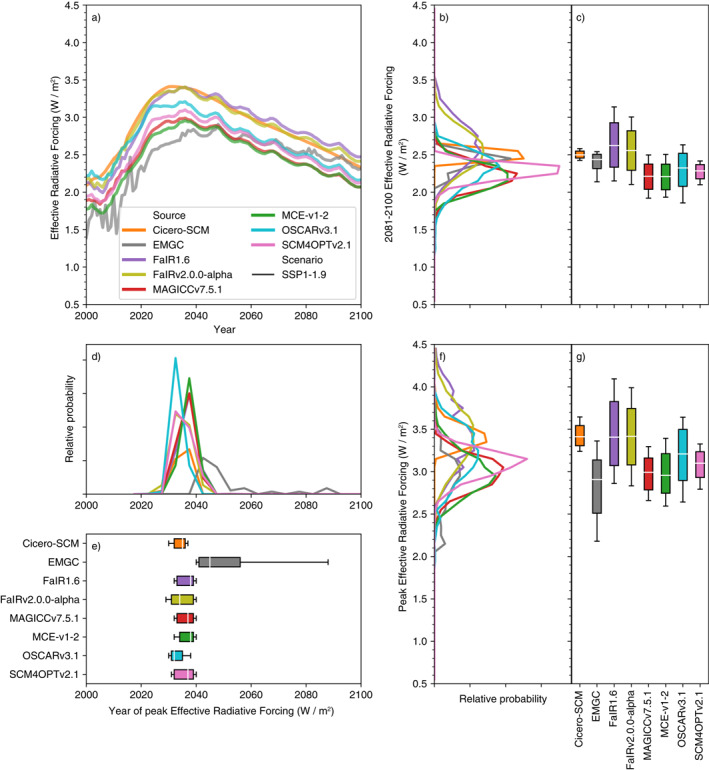
Effective radiative forcing under the very‐low‐emissions SSP1‐1.9 scenario. (a) Median effective radiative forcing projections from 1995 to 2100 for each RCM; (b) distribution of 2081–2100 mean effective radiative forcing from each RCM; (c) very likely (whiskers), likely (box), and central (white line) 2081–2100 mean effective radiative forcing estimate from each RCM; (d) as in (b) except for the year in which effective radiative forcing peaks; (e) as in (c) except for the year in which effective radiative forcing peaks; (f) as in (b) except for the peak effective radiative forcing; (g) as in (c) except for the peak effective radiative forcing.

Compared to temperatures, there is less variance in end of century total effective radiative forcing projections (Figure 4, Supplementary Figure S15 and S16). This finding reinforces the understanding that the parameterization of the climate response to effective radiative forcing is a key driver of climate projection uncertainty.

In SSP1‐1.9, 2081–2100 mean total effective radiative forcing varies from 2.2 to 2.6 W/m^2^. The fifth percentile ranges from 1.8 to 2.1 W/m^2^ across the models (excluding CICERO‐SCM which has an extremely narrow range). The spread is larger for the 95th percentile, which ranges from 2.4 to 3.2 W/m^2^. This pattern, of uncertainty being higher for upper percentiles than lower percentiles, is seen across other key scenarios and highlights that the high‐end effective radiative forcing projections are much more uncertain than the median and low‐end effective radiative forcing projections.

In SSP1‐2.6 (Supplementary Figure S15, once again excluding CICERO‐SCM because of its narrow range) median 2081–2100 total effective radiative forcing ranges from 2.9 to 3.4 W/m^2^ while the fifth percentile only ranges from 2.4 to 2.7 W/m^2^ and the 95th percentile has a much wider range of 3.1 to 4.1 W/m^2^. Under SSP5‐8.5 (Supplementary Figure S16, excluding EMGC and CICERO‐SCM as outliers), median 2081–2100 total effective radiative forcing ranges from 8.0 to 9.3 W/m^2^ while the fifth percentile only ranges from 7.4 to 7.8 W/m^2^ and the 95th percentile has a much wider range of 8.4 to 11.0 W/m^2^.

**Figure 5:**
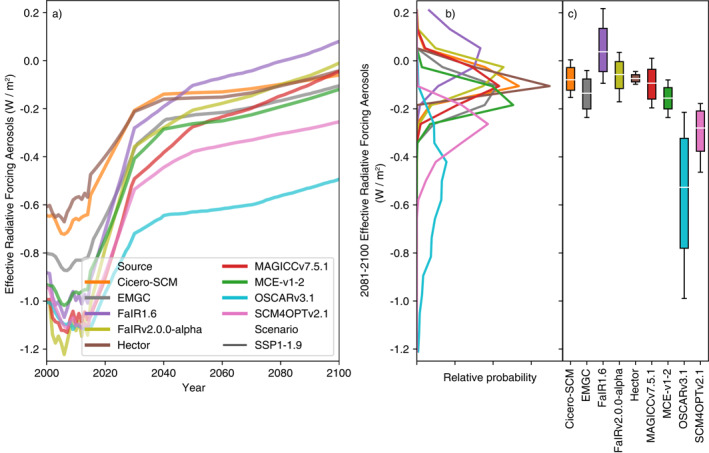
As in panels (a–c) of Figure 4, except for effective radiative forcing due to aerosols.

The approximate agreement in total effective radiative forcing is reflected in the agreement of each of the key contributors to this total, namely CO_2_ and aerosol effective radiative forcing (Figure 5 and Supplementary Figures S17–S29, which also show ERF output up to the year 2300). The key exceptions to this are SCM4OPTv2.1 and OSCARv3.1’s aerosol effective radiative forcing. This negative aerosol forcing is driven by SCM4OPTv2.1 and OSCARv3.1’s inclusion of a climate feedback on aerosol effective radiative forcing. The climate feedback makes their median end of century aerosol effective radiative forcing 0.3–0.8 W/m^2^ more negative than other RCMs across the scenarios, although the effect is stronger in OSCARv3.1 than in SCM4OPTv2.1. The strong aerosol forcing is somewhat compensated by other forcing agents although both these models have long‐term ERF which is at the low end of the RCM ensemble under SSP5‐8.5 (Supplementary Figure S14). The different aerosol ERF parameterizations warrant further attention, particularly because models without this aerosol ERF—climate feedback may be underestimating the spread in future temperature projections.

#### 4.2.3 Carbon Cycle

Moving beyond effective radiative forcing and its temperature response, we consider the behavior of the carbon cycle in the different RCMs. Clearly, the analysis presented here covers only a limited subset of the full range of carbon cycle behavior and metrics. The analysis is intended to highlight variance in carbon cycle behavior across the RCMs, providing the motivation for a more detailed future analysis. We use the emissions‐driven ESM‐SSPX‐Y.Y set of scenarios, in which emissions of CO_2_ are prescribed and atmospheric CO_2_ concentrations are allowed to freely evolve (in contrast to the SSP experiments in which CO_2_ concentrations are prescribed).

**Figure 6:**
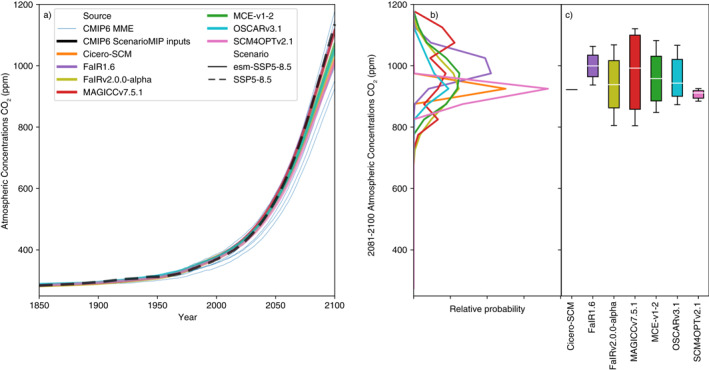
Atmospheric CO_2_ concentration projections in the esm‐SSP5‐8.5 experiment. (a) Atmospheric CO_2_ concentration projections from 1995 to 2100. We show the median RCM projections (colored lines), prescribed CMIP6 ScenarioMIP input concentrations from the SSP5‐8.5 concentration‐driven experiment (dashed black line) and available CMIP6 model projections (thin blue lines, we show a single ensemble member for each CMIP6 model to preserve the CMIP6 models’ natural variability signal); (b) distribution of 2081–2100 mean atmospheric CO_2_ concentration projections from each RCM; (c) very likely (whiskers), likely (box), and central (white line) 2081–2100 mean atmospheric CO_2_ concentration projections estimate from each RCM. *Note*. that FaIR1.6 data is taken from the esm‐SSP5‐8.5‐allGHG simulations because esm‐SSP5‐8.5 simulations are not available.

There are considerable variations between the RCMs which submitted relevant results (Supplementary Figures S30 and S31, Figure 6). In esm‐SSP1‐1.9 (Supplementary Figure S30, excluding CICERO‐SCM because of its narrow range), the spread in median peak atmospheric CO_2_ concentrations (430–450 ppm) is similar to the spread in 2081–2100 median concentrations (385–410 ppm). Similarly, in esm‐SSP1‐2.6 (Figure S31, again excluding CICERO‐SCM), the spread in median peak atmospheric CO_2_ concentrations (450–480 ppm) shows a spread similar to the spread in 2081–2100 median concentrations (430–460 ppm). Under both scenarios, there are wide variances in percentile ranges across the models, with MAGICC7 showing the largest uncertainty in 2081–2100 atmospheric CO_2_ concentrations and SCM4OPTv2.1 showing the least (arguably, this model's range is overly confident). The considerable spread in projections from the models highlights the importance of carbon cycle uncertainty for emissions‐driven projections. The spread reinforces the need for a detailed study into available techniques for evaluating and potentially constraining carbon cycle behavior. Such a study would provide information about whether any of these projections can be ruled out based on other lines of evidence.

Next, we consider esm‐SSP5‐8.5, the only scenario with available CMIP6 Earth System Model results (Figure 6). Median 2081–2100 atmospheric CO_2_ concentrations range from 920 ppm to 1,000 ppm while fifth percentile and 95th percentile concentrations range from 800 to 930 ppm and 910 ppm to 1,130 ppm respectively. MAGICC7 once again shows the largest uncertainties, but is more similar to the other RCMs than in the other scenarios. These comparisons highlight differences in the dynamics of the carbon cycle (and its feedbacks) in the various RCMs: uncertainties widen to a greater extent in higher‐warming scenarios in FaIR1.6, FaIRv2.0.0‐alpha, MCE‐v1‐2, OSCARv3.1, and SCM4OPTv2.1 compared to MAGICC7.

Median atmospheric CO_2_ projections from all of the RCMs lie within the plume of available CMIP6 results (Figure 6). FaIR1.6 lies at the top end of the CMIP6 plume, and its 5–95th range does not include low‐end CMIP6 results. In contrast, SCM4OPTv2.1 lies at the bottom end of the CMIP6 plume. FaIR‐v2.0.0‐alpha, MAGICC7, MCE‐v1‐2 and OSCARv3.1 approximately span the CMIP6 range, with FaIR‐v2.0.0‐alpha's and MCE‐v1‐2’s ranges being almost exactly in line with the CMIP6 range while MAGICC7’s projections are slightly wider than the CMIP6 range and OSCARv3.1’s projections are slightly narrower than the CMIP6 range. CICERO‐SCM does not include uncertainty in the carbon cycle, nor temperature feedbacks on the carbon cycle, hence produces only a single best‐estimate projection.

Despite the limits of our carbon cycle evaluation, it is notable that the CMIP6 ScenarioMIP input concentrations are generally higher than the RCMs’ medians in emissions‐driven runs across all considered scenarios. Emissions‐driven scenario data from CMIP6 ESMs is almost exclusively related to the esm‐SSP5‐8.5 experiment. Hence, while the pattern appears to be that the prescribed SSP5‐8.5 CMIP6 concentrations are at the high‐end of the range compared to the esm‐SSP5‐8.5 CMIP6 ESM results, there is little data with which to determine whether the prescribed CO_2_ concentrations in the low‐emissions scenarios would be within the projected concentration change by emission‐driven ESM models. In hindsight, the input atmospheric CO_2_ concentrations used in the concentration‐driven runs may turn out to be at the high‐end of CMIP6 ESM results across a range of scenarios. Given that only one set of input concentrations can be used in CMIP6, it is not surprising that the CO_2_ concentrations prescribed for CMIP6 experiments do not sit exactly in the middle of later emissions‐driven runs. The opposite was observed in CMIP5: the input CO_2_ concentrations (derived with MAGICC6) were found to be in the lower‐half of the results from the CMIP5 emissions‐driven runs (Friedlingstein et al., 2014). The CMIP6 concentrations were derived using an alpha version of MAGICC7, calibrated to approximately the median of the CMIP5 ESM carbon cycle responses with the inclusion of permafrost CO_2_ and methane feedbacks (Meinshausen, Nicholls, et al., 2020). Choosing a carbon cycle parameterization more in line with the median of CMIP5 models appears to have led to CO_2_ concentrations which are now in the upper‐half of CMIP6 ESM projections (Figure 6). Whenever a single estimate of the relationship between CO_2_ emissions and concentrations is used, there is always the risk that it will not be the central estimate of the next generation of ESMs as our understanding of the carbon cycle improves and the ensembles of participating ESMs changes in each intercomparison phase. While this does not invalidate the design of concentration‐driven experiments which are developed in this way, it must be kept in mind when relating emissions scenarios and the output of concentration‐driven CMIP experiments.

#### 4.2.4 All Greenhouse Gas Emissions‐Driven Runs

The final set of experiments we present are the experiments which are most relevant to WG3: all greenhouse gas emissions‐driven runs. As discussed in Section 1, WG3 describes scenarios in terms of their emissions hence needs models which can run in a fully emissions‐driven setup. The cost of running ESMs for a large number of scenarios and parameter configurations in such a setup is computationally prohibitive (and few ESMs include key feedbacks such as methane permafrost and wetland emissions), hence there is a paucity of data against which to evaluate the projections of RCMs in such experiments. Nonetheless, here we present the results of such experiments in the hope that they will inspire further efforts into how to validate RCMs in this fully coupled, all greenhouse gas emissions‐driven setup.

**Figure 7:**
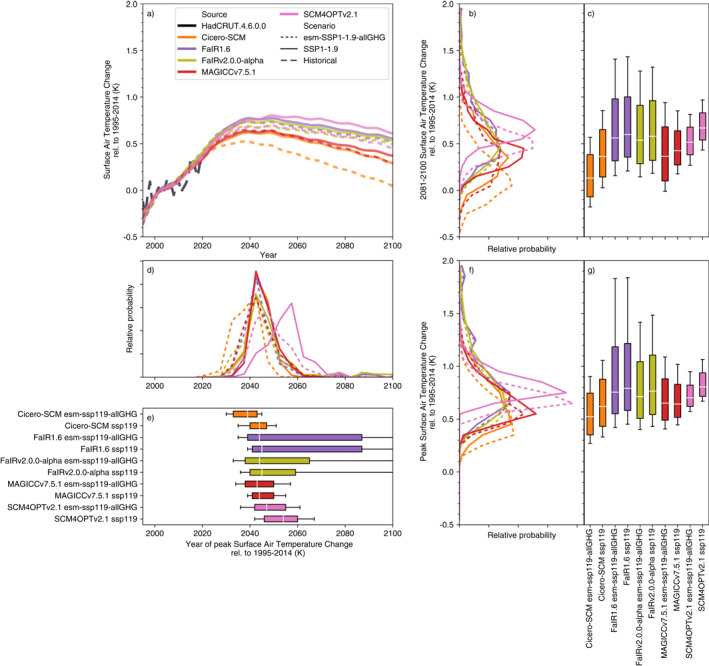
Surface air temperature (also referred to as global‐mean surface air temperature, GSAT) change in the concentration‐driven SSP1‐1.9 experiment and the all greenhouse gas emissions‐driven esm‐SSP1‐1.9‐allGHG experiment. (a) GSAT projections from 1995 to 2100. We show the median RCM projections (colored lines) for the concentration‐driven experiment (solid) and all greenhouse gas emissions‐driven experiment (dashed) as well as observations up to 2019 (dashed black line); (b) distribution of 2081–2100 mean GSAT for each scenario from each RCM; (c) very likely (whiskers), likely (box), and central (white line) 2081–2100 mean GSAT estimate for each scenario from each RCM; (d) as in (b) except for the year in which GSAT peaks; (e) as in (c) except for the year in which GSAT peaks; (f) as in (b) except for the peak GSAT; (g) as in (c) except for the peak GSAT. All results are shown relative to the 1995–2014 reference period.

Five models (CICERO‐SCM, FaIR1.6, FaIRv2.0.0‐alpha, MAGICC7, and SCM4OPTv2.1) have submitted results for the all greenhouse gas emissions‐driven experiments. The results suggest that the all greenhouse gas emissions‐driven runs are cooler and peak earlier than the concentration‐driven runs (Figure 7, Supplementary Figure S32 and S33). However, the magnitude of the difference varies across the models. For median projections, MAGICC7 suggests the smallest difference between concentration‐driven and all greenhouse gas emissions‐driven runs while CICERO‐SCM and SCM4OPTv2.1 imply differences of up to 0.3°C for peak and 2081–2100 warming and a peak in warming up to 10 years earlier. The range of projections in the all greenhouse gas emissions‐driven runs are generally about the same or slightly wider than in the concentration‐driven runs, with MAGICC7 showing the largest increase in projection ranges.

The lower‐warming and wider projection ranges seen in all greenhouse gas emissions‐driven runs are consistent with two other pieces of knowledge. The first is that median CO_2_ concentrations are lower in all greenhouse gas emissions‐driven runs than in concentration‐driven runs (Section 4.2.3). The second is that carbon cycle and other greenhouse gas cycle uncertainties are included in temperature projections in all greenhouse gas emissions‐driven runs, while these uncertainties are missing in concentration‐driven runs. The difference between the all greenhouse gas emissions‐driven runs and concentration‐driven runs reinforces the need for further consideration of RCM behavior beyond the climate response to ERF.

### 4.3 Further Discussion

Our results prompt consideration of a number of further points. First, the assessment performed here provides a way to easily identify differences between an RCM’s behavior and the assessed range of a particular metric. Such differences are important to quantify, as they can reveal biases in a probabilistic distribution. The quantification makes it possible for the users of these distributions to identify where the biases might impact their own conclusions.

There are, however, cases where the issue lies in the combination of the proxy assessed ranges taken together, rather than in the probabilistic distributions. In this study, we used a combination of ECS from the literature (based on multiple lines of evidence), TCR from constrained CMIP6 models and TCRE from unconstrained CMIP6 Earth System Models. This combination is likely to be slightly inconsistent. Unfortunately, inconsistency between metric values is an inevitable risk of using independent lines of evidence. The potential inconsistency could in part explain our finding that the RCMs’ TCR ranges are generally too high, while their ECS and TCRE ranges are generally too low. To explain the inconsistency in more detail, first consider the ratio between TCR and ECS, i.e., the realized warming fraction. The realized warming fraction implied by our TCR and ECS distributions is around 0.5. This is at the low end of the assessment by Millar, Otto, et al. (2015). Hence, it can be argued that greater consistency within the proxy assessment would be achieved if either our proxy assessed TCR values were larger, or our proxy assessed ECS values were smaller. Similarly, the airborne fraction implied by our TCR and TCRE assessment is around 0.65. This is at the high‐end of the CMIP5 and CMIP6 range quantified by Arora, Katavouta, et al. (2020). Once again, it can be argued that greater consistency within the proxy assessment would be achieved if either our proxy assessed TCR values were larger, or our proxy assessed TCRE values were smaller. Identifying such inconsistencies is a useful secondary benefit of exercises such as the one performed here.

Next, while they are a useful way of quickly visualizing a model's agreement with the (here proxy) assessed ranges, summary tables of the form of Table 3 hide the full story. Specifically, for timeseries based variables, assessed ranges can only consider the trend or change between specific reference periods and don’t consider the entire timeseries as a whole.

**Figure 8:**
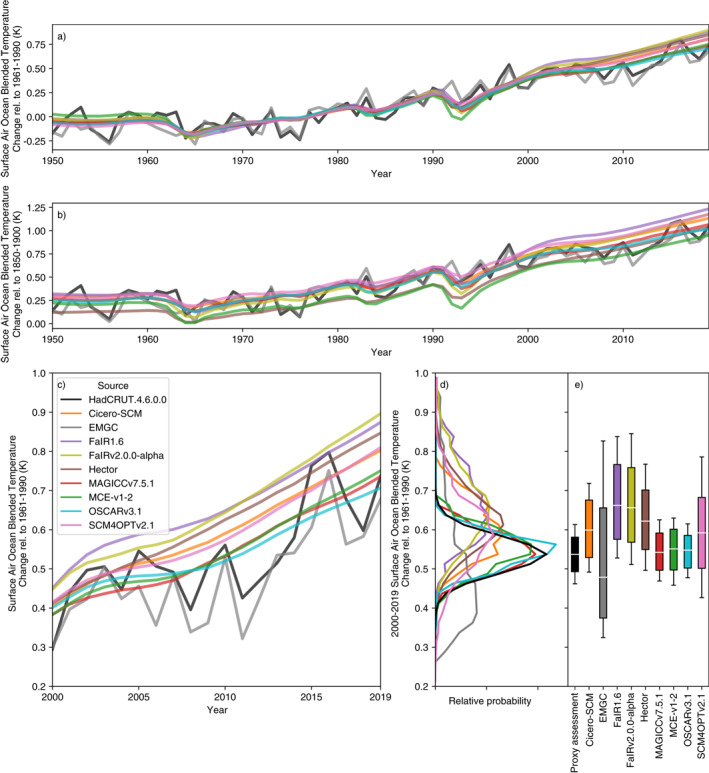
Historical surface air ocean blended temperature change (also referred to as global‐mean surface temperature, GMST) from each RCM. We compare observations from HadCRUT4.6.0.0 (Morice, Kennedy, Rayner, & Jones, 2012) (solid black line) to the distribution from each RCM (colored lines). All panels use 1961–1990 as the reference period, the same reference period as is used in our proxy assessed ranges, except (b) which uses 1850–1900. (a, b) median GMST from 1950 to 2019; (c) median GMST from 2000 to 2019 (the proxy assessment period); (d) distribution of 2000–2019 mean GMST from each RCM and the proxy assessed range; (e) Very likely (whiskers), likely (box), and central (white line) estimate of 2000–2019 mean GMST from each RCM and the proxy assessed range. The historical simulation has been extended with SSP2‐4.5 for the period 2015–2019.

Not considering the entire timeseries can lead to problematic interpretations of the agreement between a model and the assessment. A clear example here is historical surface air ocean blended temperature change. In our proxy assessment, we focused on 2000–2019 warming relative to the 1961–1990 reference period. On this measure, many of the RCMs were too warm compared to observations. However, the level of agreement is clearly reference period dependent (Figures 8a and 8b). In Figure 8a, which uses a 1961–1990 reference period, MAGICC7, MCE‐v1‐2, and OSCARv3.1 show the best agreement with observations (as also seen in Table 3). However, if we use a different reference period, e.g., 1850–1900 (Figure 8b), that impression changes with Hector, MAGICC7, and OSCARv3.1 being the closest to observations in the recent period.

Considering the entire timeseries provides a more robust check on model behavior. Fitting only to one evaluation and reference period can be achieved by slightly adjusting different model behavior, e.g., aerosol effective radiative forcing. However, if the entire timeseries are considered with multiple reference periods, such tuning quickly becomes impossible and the check provides detail into how well a model's dynamics are consistent with observations.

Moving away from evaluating the models, we find that higher historical warming, ECS and TCR values generally lead to higher‐warming projections (an intuitive result). Hector provides an exception to this pattern, with relatively low temperature projections, especially in SSP1‐1.9, despite its relatively high historical warming and TCR.

In the strong mitigation scenarios (SSP1‐1.9 and SSP1‐2.6), there is agreement to within ∼ 0.1°C in future projections (both best‐estimate and range) between the models which best reflect historical warming (MAGICC7, MCE‐v1‐2, and OSCARv3.1). This agreement suggests that constraining greatly increases confidence in future projections. However, a limited set of models also provided probabilistic distributions that are constrained to match HadCRUT.5.0.1.0 (Morice, Kennedy, Rayner, Winn, et al., 2021), which is significantly warmer than the HadCRUT.4.6.0.0 based constraint used in the rest of the study. The future projections from these HadCRUT.5.0.1.0‐constrained distributions are noticeably warmer (Supplementary Figures S34–S36) than projections from HadCRUT.4.6.0.0‐constrained distributions, which demonstrates that projections are sensitive to the choice of constraint.

Given the sensitivity of conclusions to the constraint, the use of constraints must be carefully considered as it could lead to overconfidence (Sanderson et al., 2017). Even though considerable care is taken both here and elsewhere to identify and use relevant, physically justifiable, constraints, it is still possible that future research may show that the constraints are leading to overconfident future projections. Having said this, Herger et al. (2019) suggest that using multiple constraints, as is done by many RCMs here, reduces the likelihood of overconfidence.

Studies which constrain the raw CMIP6 model ensemble help explain the difference between the RCM‐based results presented here and the raw CMIP6 model ensemble. Brunner et al. (2020), Liang et al. (2020), and Tokarska et al. (2020) all find significant reductions in both the best‐estimate and 5–95% range GSAT projections after applying observed‐warming constraints to the CMIP6 model ensemble. For the SSP1‐2.6 and SS5‐8.5 scenarios respectively, these studies find 5–95% GSAT (relative to 1995–2014) ranges of: Tokarska et al. (2020): 0.41–1.46 and 2.26–4.60°C; Liang et al. (2020) 0.52–1.66 and 2.72–4.77°C and Brunner et al. (2020) 0.61–1.85 and 2.72–4.86°C. These estimates, particularly for the SSP1‐2.6 scenario, are slightly wider than our results based on RCMs. However, the constrained CMIP6 estimates are much closer to our RCM‐based estimates than the raw CMIP6 model ensemble, in particular for the 95th percentile. This suggests that the majority of the difference between our RCM‐based results and the raw CMIP6 model ensemble is explained by the constraining applied to the RCMs, rather than structural differences between RCMs and CMIP6 models (although structural differences may explain the disagreement between constrained CMIP6 output and our results). Further studies are needed to explore the validity of the constraining approaches for both ESMs and RCMs—as investigated here—but this study lays the foundation for systematically investigating probabilistic RCM ensembles in more detail.

Given the proxy assessment and results, we make one final observation: to extrapolate assessed warming ranges from one set of scenarios (e.g., the RCP or SSP‐based scenarios) to a wider set of scenarios, it may be beneficial to include a benchmark of assessed future warming under a selection of scenarios. This benchmark could be taken from other studies, e.g., those that constrain CMIP projections (for the limited number of scenarios run by CMIP) based on historical observations (e.g., Brunner et al., 2020; Liang et al., 2020; Tokarska et al., 2020). Adding such a benchmark to the historical observations, present‐day assessments and idealized metrics used in this study would highlight where future warming significantly diverges from other lines of evidence. Including scenarios with similar end of century total ERF but different transient evolutions (like the SSP4‐3.4 and SSP5‐3.4‐overshoot scenario pair) would provide an even stronger check of the models’ transient response. Such quantifications could be key when assessing future projections under large sets of scenarios, like the WG3 scenario database climate assessment. Of course, the risk of adding such benchmarks is an artificial narrowing of uncertainty in projected warming. Hence, future projections should only be included where there is a clear need and justification for consistency between the RCMs’ projections and the projections from other lines of evidence.

## 5 Future Work

This exercise is a first step toward more comprehensive, routine evaluation of RCMs’ probabilistic parameter ensembles and their corresponding projections. However, there is still much room for future work to improve on this study and the first phase of RCMIP. As a first suggestion, repeating this exercise with the assessed ranges from Working Group 1 of the Intergovernmental Panel on Climate Change’s Sixth Assessment Report (due in mid‐2021) would provide an evaluation of the extent to which RCMs can capture the latest international assessment of the scientific literature.

This future work could go beyond evaluation and also diagnose the root causes of differences between the models. One obvious area for examination would be the aerosol ERF, particularly the inclusion of a climate feedback in aerosol ERF parameterizations. Such an exercise could also provide greater insights into differences between the constrained RCMs’ probabilistic distributions, the raw CMIP6 multimodel ensemble and constrained CMIP6 output (building on the discussion in Section 4.3).

A clear limitation of this study is the relative lack of examination of carbon cycle behavior and carbon cycle related metrics. Given the importance of the carbon cycle for emissions‐driven projections, this is another clear area for future work. In the limited examination we have performed, we chose to focus on emissions‐driven simulations. This choice provides the cleanest comparison between RCMs and CMIP6 models, given that many RCMs do not separate the land and ocean carbon pools, although it limits us to a relatively small set of CMIP6‐comparison data (given that only few emissions‐driven simulations (Jones et al., 2016) have been run by CMIP6 models). An increase in the number of emissions‐driven CMIP6 ESM model output, particularly for mitigation scenarios, would greatly aid such evaluations. Using the concentration‐driven simulations in future work will also provide a greater set of comparison data and will facilitate evaluation of RCMs’ land and ocean carbon cycles under more varied scenarios.

Finally, given how RCMs are typically used by WG3, it appears that a truly thorough evaluation would need to consider a larger set of individual steps in the emissions‐climate change cause‐effect chain. Such an evaluation would provide insights into the drivers of differences between future projections based on the concentration‐driven experiments typical of CMIP and results based on the all greenhouse gas emissions‐driven experiments required by WG3. While it is not completely clear to us which components would need to be considered (and which could be ignored), a first suggestion of important components is: the carbon cycle, other earth system feedbacks, e.g., representation of permafrost, representation of aerosols, non‐CO_2_ greenhouse gas cycles, translation between changes in greenhouse gas concentrations and effective radiative forcing, ozone representation, land‐use change albedo representation, temperature response to effective radiative forcing, and all the feedbacks and interactions. To see the full picture, a broad range of literature would need to be considered as a validation source and a wide range of experiments, spanning historical, scenario‐based, and idealized experiments, would need to be performed. In performing a more thorough evaluation, an updated evaluation technique may be required. Specifically, using percentage differences from the assessed range will lead to problems when the assessed range is close to or spans zero. Hence, more sophisticated ways of evaluating the agreement between model results and assessed ranges may be required. For reasons of scope, we have not achieved such a thorough evaluation here, but we hope that this work provides a basis upon which future work can aim for the lofty goal of more complete evaluation of all of the relevant parts of the climate system.

## 6 Conclusions

We have found that the best performing RCMs can match our proxy assessment across a range of climate metrics. However, no RCM matched the proxy assessment across all metrics. At the same time, all RCMs matched the proxy assessment well for at least one metric.

Our evaluation is the first multimodel comparison of probabilistic projections from RCMs. This exercise provides a unique insight into RCMs probabilistic parameter ensembles, specifically how they compare with a set of proxy assessed ranges, which reflect wider scientific understanding of key climate metrics, and the implications of differences in probabilistic distributions for climate projections across a range of climate variables and scenarios.

Notably, although unsurprisingly, we found that models whose probabilistic distribution were constrained to the proxy assessed ranges were better able to reflect the proxy assessed ranges. This point is notable because it makes clear that if RCMs are to be used as integrators of knowledge, conveying multiple lines of evidence from one domain to another (e.g., IPCC WG1 to IPCC WG3), then RCMs whose probabilistic distributions have been constrained to the intended lines of evidence are likely to be the best tool.

Even among models which had similar levels of agreement with the proxy assessment, some divergence in future projections was observed. Given the various model structures that the reduced complexity models employ, ranging from linearized impulse response functions to 50‐layer ocean models, it is not surprising that models may diverge in scenarios that go significantly beyond the domain of the validation data. Adding constraints on future performance, i.e., extending the domain of validation data (e.g., based on an independent assessment of warming in a limited subset of scenarios) would likely reduce the divergence, although such extra constraints should be carefully considered given that they risk artificially narrowing projection uncertainty.

While exercises such as the one performed here can provide helpful information about where the biases may lie, they cannot provide definitive answers about what the future holds. It is possible to make judgments about what is more reasonable based on the evaluation performed here, and to rule out clearly incorrect projections, yet it must be recognized that a definitive answer is impossible: we will not know which projections are correct until we get there, by which time it is too late for climate policy. Hence, while it is important to continue to evaluate and improve our models to remove as many sources of error as possible, it is also important that research into decision making under uncertainty (e.g., Dittrich et al., 2016; Weaver et al., 2013) continues to develop and be used because the uncertainty in projections will not disappear anytime soon, never in fact. In addition, those who use RCMs for climate projections should carefully consider how they are going to use the RCMs and how they are going to validate them before making conclusions about the implications of their projections.

In addition, we found that many of the RCMs did not reproduce the high warming seen in CMIP6 models. However, studies which constrain CMIP6 models based on observational constraints also exclude such high warming which suggests that the lack of high warming is due to the constraining applied to the RCMs, rather than structural differences between RCMs and CMIP6 models. Beyond the question of temperature projections, we found that the prescribed CO_2_ concentrations used in the CMIP6 SSP‐based experiments are at the high‐end of projections made with historically constrained carbon cycles. Although, further investigations into carbon cycle behavior are required to provide a clearer picture of the influence of carbon cycle uncertainties on emissions‐driven projections. Finally, we observed that a change in reference period significantly altered how well some models agreed with observations, reinforcing the need to consider more than one reference period when evaluating models.

With sufficient validation, RCMs provide a unique synthesis tool to integrate the latest scientific understanding, including its uncertainties, along the complex cause‐effect chain from emissions to global‐mean temperatures. Integrating this understanding in an internally consistent RCM framework, with all the implicit cross‐correlations, is our best method to inform decision making and other scientific domains, for example the likelihood of exceeding a given global‐mean temperature threshold under a specific emissions scenario. Further developing these tools opens vast opportunities to go beyond global‐mean variables and temperature changes, and to robustly represent the complex science beneath.

## Supporting information

Supporting Information S1

## Data Availability

Data and code to produce all the figures and tables can be obtained from https://doi.org/10.5281/zenodo.4269711.
