# Supplementary material for: Reduced Complexity Model Intercomparison Project Phase 2: Synthesizing Earth System Knowledge for Probabilistic Climate Projections
Source: Earths Future. 2021 Jun 4;9(6):e2020EF001900. doi: 10.1029/2020EF001900 (PMC8243973; doi:10.1029/2020EF001900)
Supplement: Supplementary file 1 — Supporting Information S1 [file EFT2-9-e2020EF001900-s001.pdf]

Supporting Information for "Reduced Complexity Model Intercomparison Project Phase 2: Synthesising Earth system knowledge for probabilistic climate projections"

Z. Nicholls[1,2], M. Meinshausen[1,2,3], J. Lewis[1], M. Rojas Corradi [4,5], K. Dorheim[6], T. Gasser[7], R. Gieseke[8], A. P. Hope[9], N. J. Leach[10], L. A. McBride[11], Y. Quilcaille[7], J. Rogelj[12,7], R. J. Salawitch[11,9,13], B. H. Samset[14], M. Sandstad[14], A. Shiklomanov[15], R. B. Skeie[14], C. J. Smith[16,7], S. J. Smith[17], X. Su[18], J.Tsutsui[19], B. Vega-Westhoff[20]and D. L. Woodard[5]

[1]Australian-German Climate & Energy College, The University of Melbourne, Parkville, Victoria, Australia

[2]School of Earth Sciences, The University of Melbourne, Parkville, Victoria, Australia

[3]Potsdam Institute for Climate Impact Research (PIK), Member of the Leibniz Association, Potsdam, Germany

[4]Department of Geophysics, University of Chile, Santiago, Chile

[5]Center for Climate and Resilience Research, CR2, Santiago, Chile

[6]Pacficic Northwest National Laboratory

[7]International Institute for Applied Systems Analysis, Laxenburg, Austria

[8]Independent researcher, Potsdam, Germany

[9]Department of Atmospheric and Oceanic Science, University of Maryland-College Park, College Park, 20740, USA

[10]Atmospheric, Oceanic, and Planetary Physics, Department of Physics, University of Oxford, United Kingdom

[11]Department of Chemistry and Biochemistry, University of Maryland-College Park, College Park, 20740, USA

[12]Grantham Institute, Imperial College London, London, UK

[13]Earth System Science Interdisciplinary Center, University of Maryland-College Park, College Park, 20740, USA

[14]CICERO Center for International Climate Research, Oslo, Norway

May 18, 2021, 12:25pm

[15]NASA Goddard Space Flight Center, Greenbelt, MD, USA 20771

[16]Priestley International Centre for Climate, University of Leeds, United Kingdom

[17]Joint Global Change Research Institute, Pacific Northwest National Laboratory, College Park, MD, USA

[18]Research Institute for Global Change / Research Center for Environmental Modeling and Application / Earth System Model Development and Application Group, Japan Agency for Marine-Earth Science and Technology (JAMSTEC), Yokohama, Japan

[19]Environmental Science Research Laboratory, Central Research Institute of Electric Power Industry, Abiko, Japan

[20]Department of Atmospheric Sciences, University of Illinois at Urbana-Champaign, Urbana, IL, USA

Contents of this file

1. Text S1: Model descriptions

2. Table S1: Comparison of the proxy assessed ranges with each model's probabilistic distribution (continues over multiple pages)

3. Figures S1 - 9: Probabilistic distributions for each model compared to the proxy assessed ranges

4. Figure S10: SSP1-2.6 GSAT projections until 2100

5. Figure S11: SSP5-8.5 GSAT projections until 2100

6. Figure S12: SSP1-1.9 GSAT projections until 2300

7. Figure S13: SSP1-2.6 GSAT projections until 2300

8. Figure S14: SSP5-8.5 ERF projections until 2300

9. Figure S15: SSP1-2.6 ERF projections until 2100

10. Figure S16: SSP5-8.5 ERF projections until 2100

11. Figure S17: SSP1-1.9 ERF projections until 2300

12. Figure S18: SSP1-2.6 ERF projections until 2300

13. Figure S19: SSP1-1.9 aerosol ERF projections until 2300

14. Figure S20: SSP1-1.9 CO_2 ERF projections until 2100

15. Figure S21: SSP1-1.9 CO_2 ERF projections until 2300

16. Figure S22: SSP1-2.6 aerosol ERF projections until 2100

17. Figure S23: SSP1-2.6 aerosol ERF projections until 2300

18. Figure S24: SSP1-2.6 CO_2 ERF projections until 2100

19. Figure S25: SSP1-2.6 CO_2 ERF projections until 2300

20. Figure S26: SSP5-8.5 aerosol ERF projections until 2100

21. Figure S27: SSP5-8.5 aerosol ERF projections until 2300

22. Figure S28: SSP5-8.5 CO_2 ERF projections until 2100

23. Figure S29: SSP5-8.5 CO_2 ERF projections until 2300

24. Figure S30: ESM-SSP1-1.9 atmospheric CO_2 concentration projections until 2100

25. Figure S31: ESM-SSP1-2.6 atmospheric CO_2 concentration projections until 2100

26. Figure S32: SSP1-2.6 and ESM-SSP1-2.6-allGHG GSAT projections until 2100

27. Figure S33: SSP5-8.5 and ESM-SSP5-8.5-allGHG GSAT projections until 2100

28. Figure S34: SSP1-1.9 HadCRUT.4.6.0.0 and HadCRUT.5.0.1.0 constrained GST projections until 2300

29. Figure S35: SSP1-2.6 HadCRUT.4.6.0.0 and HadCRUT.5.0.1.0 constrained GST projections until 2300

30. Figure S36: SSP5-8.5 HadCRUT.4.6.0.0 and HadCRUT.5.0.1.0 constrained GST projections until 2300

Text S1.

CICERO-SCM

The CICERO Simple climate model (CICERO-SCM) consists of a carbon cycle model (Joos et al., 1996), simplified expressions relating emissions of components to forcing, either directly or via concentrations (Etminan et al., 2016; Skeie et al., 2017) and an energy balance/upwelling diffusion model (Schlesinger et al., 1992). A detailed description of the CICERO-SCM is presented in Skeie et al. (2017) with recent updates in Nicholls et al. (2020). The energy balance/upwelling diffusion model calculates warming separately for the two hemispheres and includes 40 vertical layers in the ocean. The parameters that govern the mixing of heat in the ocean as well at the climate sensitivity and radiative forcing is estimated in a Bayesian approach using observational based time series of global mean surface temperature change and ocean heat content and prior estimates of radiative forcing times series (Skeie et al., 2018). The posteriori distribution of the parameters in the energy balance/upwelling diffusion model including the climate sensitivity and aerosol forcing are used in the probabilistic run. Uncertainties in other climate drivers are ignored. The climate sensitivity estimated in Skeie et al. (2018) is the inferred effective climate sensitivity (ECS_{inf}), known to be lower than the equilibrium climate sensitivity (ECS). This is due to the use of global-mean surface temperature instead of global-mean surface air temperature as well as feedback on long time scales that have not come into play when inferring the climate sensitivity from the historical record. The assessed range for the equilibrium climate sensitivity is therefore shifted by 0.9°C (Skeie et al., 2018)

to lower values when selecting the parameter set to be used in the probabilistic simulations. Another selection criterion is the assessed range of surface air temperature change in 1985-2014 relative to the baseline 1850-1900. The 30 040 original parameter set from Skeie et al. (2018) is subsetted using the RCMIP defined ECS distribution as the primary constraint. By binning the data, a subset following this same distribution is built. Simultaneously, the surface air temperature constraint is used as a hard cutoff to choose between parameter sets in this distribution, producing a final subset of between 550 and 600 ensemble members.

EMGC

The University of Maryland Empirical Model of Global Climate (EMGC) is a multiple linear regression energy balance representation of various factors (both natural and anthropogenic) that control global-mean surface temperature (GMST) (Canty et al., 2013; Hope et al., 2017). Values of climate feedback and ocean heat uptake efficiency are found in a regression framework constrained by observed time series of GMST, the radiative forcing due to tropospheric aerosols (AER RF), and ocean heat content. Several natural and anthropogenic components that affect GMST are also considered. Recently, we have added an interactive ocean module that represents the warming of the ocean profile in response to rising GMST (Hope et al., 2020; McBride et al., 2020). As a result, transport of heat from the atmosphere to the world's oceans evolves over time in a more realistic fashion compared to earlier versions of our model.

The EMGC forecasts of GMST all use values of radiative forcing due to future greenhouse gas abundances and aerosols prescribed by RCMIP. Values of climate feedback and ocean heat uptake for each simulation are found based upon regression analysis of the data

record for GMST from HadCRUT4 (Morice et al., 2012) that spans 1850 until the end of 1999 (an alternate parameter set is also submitted based on using the HadCRUT.5.0.1.0 dataset instead) and an ocean heat content record that is the average of data from five groups that spans 1955 to 1999 (Hope et al., 2020; McBride et al., 2020). The time series of radiative forcing due to tropospheric aerosols is scaled such that the values in 2011 (AER RF_{2011}) statistically sample the uncertainty in the value of AER RF_{2011} given by Chapter 8 of AR5 (Myhre et al., 2013). Projections of GMST are based upon analyses of the subset of the 160,000 possible combinations of climate feedback and AER RF_{2011} that provide a good fit (reduced-chi-squared metric less than or equal to 2) to the observed variation in GMST and OHC, as described in Hope et al. (2020) and McBride et al. (2020). The largest factor driving spread in our future projections of GMST from the ensemble of model runs that satisfy the reduced-chi-squared metric is imprecise knowledge of the radiative forcing of climate by tropospheric aerosols over the historical time period. Ensemble members with largest future warming are characterized by values of AER RF_{2011} towards the low end of the distribution (i.e., -1.5 W m^{-2} to -1.9 W m^{-2}) and the ensemble members with smallest future warming are characterized by values of AER RF_{2011} towards the high end of the distribution (i.e., -0.1 W m^{-2} to -0.4 W m^{-2}).

FaIRv1.6.1

The Finite-amplitude Impulse Response (FaIR) model is an emissions-driven simple climate model written in Python. Since the v1.3 model description paper (Smith et al., 2018), a number of features have been added. FaIR v1.6 separately reports greenhouse gas forcings from 28 different fluorinated species (which were all aggregated in FaIR v1.3), and breaks aerosol forcing down into five direct species and indirect aerosol forcing.

Functionality has been added for running experiments in concentration-driven mode and for deriving CO_2 emissions from prescribed concentrations, enabling a greater set of the RCMIP experiments to be run. Additionally, it is now possible to run FaIR using the parameters of the two-layer model defined by Geoffroy, Saint-Martin, Bellon, et al. (2013), given that this model is simply a mathematical transformation of FaIR's impulse response setup (as shown by Geoffroy, Saint-Martin, Olivié, et al. (2013)). Finally, the carbon cycle has been optimised following FaIR 2.0.0, speeding up runtime (Leach et al., 2020).

An initial ensemble of 3000 members were drawn for RCMIP. Forcing uncertainties for CH4, N2O, other GHGs, tropospheric ozone, stratospheric ozone, contrails, black carbon on snow, land use change, solar and volcanic are taken from AR5 uncertainty ranges (Myhre et al., 2013). Two-layer model parameters for ocean heat exchange coefficient, climate feedback parameter, efficacy of deep ocean heat uptake and heat capacity of the mixed layer and deep ocean are sampled with distributions informed by 44 CMIP6 models built from joint kernel density distributions that take correlations of terms into account (Smith et al., submitted). The ERF from 4xCO2 is also taken from the ensemble based on abrupt-4xCO2 experiments from CMIP6 models and used to inform the uncertainty range for CO2 forcing. For ESM runs, the carbon cycle parameters (pre-industrial airborne fraction, and sensitivity to temperature and atmospheric CO2 burden) are sampled from normal distributions as in Smith et al. (2018). Direct aerosol forcing from SO2, BC and OC is sampled from CMIP6 models participating in RFMIP and AerChemMIP (Smith et al., submitted). Nitrate and secondary organic aerosol are not included. Indirect aerosol forcing is sampled by scaling the 1850-2010 aerosol forcing to a Gaussian distribution centred on -0.85 W / m^2 with standard deviation of 0.91 W / m^2. The 3000-member

prior ensemble is reduced to a final ensemble of 501 members, where this ensemble was selected from the members with the smallest RMSE for their GMST from historical (1850-2014) integrations compared to the Cowtan and Way (2014) dataset (v2.0.0) for 1850-2014. FaIR does not report GMST, but the simple assumption that GSAT anomalies are 4% greater than GMST is used based on CMIP6 models and reanalysis datasets.

FaIRv2.0.0-alpha

FaIRv2.0.0-alpha (Leach et al., 2020) is an update to the FaIR model (version 1.6 is described above). This update reduces the model's structural complexity as comprehensively as possible. The result is a set of six equations - the five equations that made up the impulse-response model used for GHG metric calculations in the IPCC 5th Assessment Report (Myhre et al., 2013), plus one additional equation that introduces a state-dependence to the carbon and methane cycles (Millar et al., 2017).

A 1 million-member ensemble is generated by perturbing parameters relating to the modelled carbon-cycle, ERF and thermal response. Prior carbon-cycle and thermal response distributions are inferred from parameter samples obtained by tuning the model to idealised experiments in the CMIP6 ensemble. The carbon-cycle was tuned to 11 models from C4MIP (Arora et al., 2020); the thermal response cycle was tuned to 28 models using a maximum likelihood method (Cummins et al., 2020). Prior ERF parameter uncertainties were taken from AR5 uncertainty ranges (Myhre et al., 2013) for all forcing classes except for aerosol-radiation and -cloud interaction. These were sampled from distributions informed by tuning the aerosol ERF parameterisations to 10 CMIP6 models (Smith et al., submitted) and then quantile mapped to match the process-based assessment in Bellouin et al. (2020).

This large prior ensemble is constrained by setting the selection probability of an individual member equal to the likelihood of its corresponding present-day level and rate of anthropogenic warming calculated using the Global Warming Index methodology (Haustein et al., 2017) (with the HadCRUT.4.6.0.0 timeseries for the main results but also the HadCRUT.5.0.1.0 timeseries for illustration). A 5 000-member subset of this constrained ensemble (total size 250 651) is used in RCMIP phase 2.

Hector v2.5.0

Hector is an open-source globally resolved, process-based carbon-climate model that calculates the annual energy fluxes between the ocean, atmosphere, and terrestrial biosphere (Hartin et al., 2015). As of Hector v2.0 (Vega-Westhoff et al., 2019), the model uses an implementation of the 1-D ocean heat diffusion model, DOECLIM (Kriegler, 2005; Urban et al., 2014). Recent model updates to v2.5.0 include: reorganizing the code as an R package, constraining pre-industrial atmospheric CO_2 to a prescribed value during model spin-up, and updating the OH lifetime.

For each scenario, Hector was run 10 000 times with parameters (equilibrium climate sensitivity, ocean heat diffusivity, and aerosol forcing) randomly sampled from the joint posterior distribution from the Vega-Westhoff et al. (2019) MCMC calibration against historical global surface temperature observations and ocean heat content. Using the parametrization from the posterior distribution we produced probabilistic Hector output for global mean air temperature, air-ocean blended temperature, and aerosol radiative forcing.

MAGICC7

MAGICC's climate core is based on a 50-layer, hemispherically resolved upwelling-diffusion-entrainment ocean model coupled to a four-box (hemispheric land/ocean) spatial resolution for effective radiative forcing. MAGICC and runs on monthly timesteps, which improves its representation of the response to volcanic eruptions compared to an annual timestep. The version of MAGICC used here (v7.4.1) is an update of MAGICC6 (Meinshausen et al., 2011) and the setup used to generate the GHG concentration projections (Meinshausen et al., 2020) for the historical and SSP-based CMIP6 experiments (Eyring et al., 2016; O'Neill et al., 2016). The key updates are the inclusion of a state-dependent climate feedback factor (previously it was only forcing-dependent) which has been calibrated to CMIP6 models (Nicholls et al., 2020), accounting for the effect of large historical anthropogenic biomass burning aerosol precursor emissions on aerosol effective radiative forcing, a nitrate aerosol forcing scheme which accounts for the sulfate competition for ammonia based on Hauglustaine, Balkanski, and Schulz (2014) and the inclusion of a non-ocean heat uptake parameterisation which represents land surface and cryosphere heat uptake in each hemisphere. In addition, it includes an updated effective radiative forcing parameterisations for CO_2, CH_4 and N_2O that capture results by Etminan et al. (2016), while allowing for a wider range of input concentrations (see Meinshausen et al. (2020)] for details).

We derive a posterior parameter distribution using the methodology of Meinshausen et al. (2009), updated to use observations of global-mean temperature up to 2019 based on HadCRUT4.6.0.0(Morice et al., 2012) (an alternate set which uses HadCRUT.5.0.0.0 is also included for sensitivity analysis) and ocean heat content up to 2018 based on von Schuckmann et al. (2020) as well as the proxy effective radiative forcing assessment used

in this study. We run a Monte Carlo Markov Chain with 20 million steps, from which we draw every 200th member, resulting in a 100 000 member posterior distribution. The probabilistic distribution used here is the result of sub-sampling the posterior distribution to draw a set of 600 parameter sets which best match the proxy assessed ranges and also maintain the covariance of MAGICC's parameters as derived from the posterior distribution.

MCE

MCE consists of a thermal response module and a carbon cycle module (the version used in this study is documented in Tsutsui (2021)). These are represented by impulse response functions (Hooss et al., 2001; Joos et al., 1996), responding to anthropogenic carbon input which then alters the ERF of the atmospheric CO_2 and natural processes in the ocean and terrestrial carbon cycle. The carbon cycle incorporates temperature feedbacks via dissociation constants in the chemical equilibrium of the carbonic acid system in seawater and the respiration of organic materials in the terrestrial biosphere. After being used in RCMIP Phase 1, the CO_2 forcing scheme was slightly changed, and schemes for non-CO_2 well-mixed GHGs were newly incorporated instead of using prescribed scenario data. The CO_2 scheme has two control parameters: one for scaling in terms of the logarithm of CO_2 concentrations, and the other for amplifying deviations from the logarithmic increase (Tsutsui, 2017). The latter is activated when the concentration exceeds a two-times level with a quadratic term, but was modified here to be linear when the concentration further exceeds a four-times level. The non-CO_2 schemes use those by Etminan et al. (2016) for CH_4 and N_2O, and a simple linear formula for halocarbon gases with their lifetimes and radiative efficiencies assessed in AR5.

The probabilistic runs were conducted with 600-member parameter sets, varied for (1) CO_2 forcing and thermal response, (2) non-CO_2 forcing scaling, and (3) ocean and land CO_2 uptake. The first sets were generated from a multivariate normal distribution built on principal components of individual parameters adjusted to CMIP5 and CMIP6 models (Tsutsui, 2017, 2020). Cross-correlation between the parameters of this group reflects the variation of the CMIP models, such that the ratio of TCR-to-ECS tends to decrease with increase in ECS, and that CO_2 forcing is weakly correlated with response properties. The second sets were implemented as scaling factors of non-CO_2 forcing, and individually generated from a probability distribution modeled for each of the prescribed likely ranges, the third sets were implemented as perturbations on the amplitudes of the impulse response function for the ocean CO_2 uptake, and on two land-CO_2 parameters for the fertilization effect and the temperature dependency of respiration. These perturbations were individually generated from a uniform distribution so that resulting carbon budgets encompass the range of those from CMIP5 and CMIP6 Earth system models presented in Arora et al. (2020). A Bayesian updating was applied to constrain the parameter sets with a Metropolis-Hastings sampling algorithm sequentially as to land CO_2 uptake, the ERF of CO_2, TCR, and the two metrics for the surface blended temperature and the ocean heat content. The land CO_2 constraint was targeted for the excess carbon at doubling along a CO_2 concentration pathway under an idealized 1%-per-year increase scenario from the CMIP Earth system models while the other constraints follow the assessed ranges. It is supposed that the second posterior conforms to the CMIP ensemble and the assessed forcing ranges, and that the last (fourth) posterior, from which the 600 members were sampled, is a compromised distribution reflecting all the metrics together with the CMIP

ensemble. For constraining the temperature and heat content metrics, a bivariate normal distribution was built with factors of 1.04 and 1.08 for conversion from the blended temperature to the air temperature, and from the ocean heat content to the total heat content, respectively.

OSCARv3.1

OSCAR v3.1 is an open-source reduced-form Earth system model, whose modules mimic models of higher complexity in a probabilistic setup (Gasser, Ciais, et al., 2017). The response of the global surface temperature to radiative forcing is the two-layer model (Geoffroy, Saint-Martin, Bellon, et al., 2013). OSCAR calculates the effective radiative forcing caused by greenhouse gases (CO_2, CH_4, N_2O, 37 halogenated compounds), short-lived climate forcers (tropospheric and stratospheric ozone, stratospheric water vapor, nitrates, sulfates, black carbon, primary and secondary organic aerosols) and changes in surface albedo. The ocean carbon cycle is based on the mixed-layer response function of Joos et al. (1996), albeit with an added stratification of the upper ocean derived from CMIP5 (Arora et al., 2013) and with an updated carbonate chemistry. The land carbon cycle is divided into five biomes and five regions, and each of the 25 biome/region combinations follows a three-box model (soil, litter and vegetation). Land cover change, wood harvest and shifting cultivation are also accounted for, thanks to a dedicated book-keeping module that allows OSCAR to estimate its own CO_2 emissions from land-use change (Gasser, Peters, et al., 2017; Gasser et al., 2020). Permafrost thaw and the consequent emission of CO_2 and CH_4 is also modeled (Gasser et al., 2018). In addition, biomass burning emissions are calculated endogenously following the book-keeping module and the

wildfire feedback. These emissions were therefore subtracted from the input RCMIP data used to drive OSCAR to avoid double counting.

In RCMIP phase 2, the same 10 000 elements of the Monte-Carlo ensemble used in RCMIP phase 1 are used. Each simulation is run using all these configurations. The parameters of OSCARv3.1 are not tuned to reflect the assessed ranges required, but instead, each configuration is weighted. The weights are determined by comparing the performances over the emissions-driven historical experiment to the assessed ranges for the cumulative net land to atmosphere and ocean to atmosphere fluxes to constrain long-term dynamics, and the rate of increase in atmospheric CO_2 for short-term dynamics. More details about this weighting approach can be found in Gasser et al. (2020). We choose not to use the historical surface air-ocean blended temperature as an additional constraint, as it causes the final range of the equilibrium climate sensitivity of OSCARv3.1 to be drastically reduced. All final outputs are provided as the resulting quantiles.

SCM4OPT v2.1

The Simple Climate Model for Optimization version 2.1 (SCM4OPT v2.1) (Su et al., 2020) is a simple climate model which can simulate the radiative forcing and global temperature change resulting from a full suite of greenhouse gases, pollutants and aerosols, as well as land-use albedo. The SCM4OPT v2.1 is designed to be lightweight and capable of being used in an integrated assessment model (IAM) with a large-scale optimization process. Compared to the older version (Su et al., 2017, 2018), we updated the ocean carbon cycle following Hector v1.0 (Hartin et al., 2015) and used the Diffusion Ocean Energy balance CLIMate (DOECLIM) model (Kriegler, 2005; Tanaka et al., 2007) to calculate global-mean temperature change. We fitted the CO2 concentration and temper-

ature change of the SCM4OPT v2.1 to the associated outputs of four RCP experiments (RCP2.6, RCP4.5, RCP6.0 and RCP8.5) of 26 coupled atmosphere ocean general circulation models (AOGCMs) in CMIP5. In addition, the method used to estimate aerosol forcing was also renewed based on OSCAR v2.2 (Gasser, Ciais, et al., 2017). However, we removed a few parameter sets which could generate unrealistic outliers, and re-tuned the forcing efficiencies and other related parameters against the aerosol forcings presented in IPCC AR5 (IPCC, 2013). An ensemble of 2000 members was adopted for RCMIP to represent the uncertainties caused by the carbon cycle, aerosol forcings and temperature change by using randomized parameter sets as described above.

References

Arora, V. K., Boer, G. J., Friedlingstein, P., Eby, M., Jones, C. D., Christian, J. R., ... Wu, T. (2013, 07). Carbon–Concentration and Carbon–Climate Feedbacks in CMIP5 Earth System Models. *Journal of Climate*, *26*(15), 5289-5314. Retrieved from https://doi.org/10.1175/JCLI-D-12-00494.1 doi: 10.1175/JCLI-D-12-00494.1

Arora, V. K., Katavouta, A., Williams, R. G., Jones, C. D., Brovkin, V., Friedlingstein, P., ... Ziehn, T. (2020). Carbon–concentration and carbon–climate feedbacks in cmip6 models and their comparison to cmip5 models. *Biogeosciences*, *17*(16), 4173–4222. Retrieved from https://bg.copernicus.org/articles/17/4173/2020/ doi: 10.5194/bg-17-4173-2020

Bellouin, N., Davies, W., Shine, K. P., Quaas, J., Mülmenstädt, J., Forster, P. M., ... Myhre, G. (2020). Radiative forcing of climate change from the copernicus reanalysis of atmospheric composition. *Earth System Science Data*, *12*(3), 1649–1677. Retrieved from https://essd.copernicus.org/articles/12/1649/2020/

doi: 10.5194/essd-12-1649-2020

Canty, T., Mascioli, N. R., Smarte, M. D., & Salawitch, R. J. (2013). An empirical model of global climate – part 1: A critical evaluation of volcanic cooling. *Atmospheric Chemistry and Physics*, *13*(8), 3997–4031. Retrieved from https://www.atmos-chem-phys.net/13/3997/2013/ doi: 10.5194/acp-13-3997-2013

Cowtan, K., & Way, R. G. (2014). Coverage bias in the hadcrut4 temperature series and its impact on recent temperature trends. *Quarterly Journal of the Royal Meteorological Society*, *140*(683), 1935–1944. doi: 10.1002/qj.2297

Cummins, D. P., Stephenson, D. B., & Stott, P. A. (2020). Optimal estimation of stochastic energy balance model parameters. *Journal of Climate*, *33*(18), 7909–7926.

Etminan, M., Myhre, G., Highwood, E. J., & Shine, K. P. (2016, dec). Radiative forcing of carbon dioxide, methane, and nitrous oxide: A significant revision of the methane radiative forcing. *Geophysical Research Letters*, *43*(24), 12,614–12,623. doi: 10.1002/2016gl071930

Eyring, V., Bony, S., Meehl, G. A., Senior, C. A., Stevens, B., Stouffer, R. J., & Taylor, K. E. (2016). Overview of the coupled model intercomparison project phase 6 (cmip6) experimental design and organization. *Geoscientific Model Development (Online)*, *9*(LLNL-JRNL-736881).

Gasser, T., Ciais, P., Boucher, O., Quilcaille, Y., Tortora, M., Bopp, L., & Hauglustaine, D. (2017). The compact earth system model oscar v2.2: description and first results. *Geoscientific Model Development*, *10*(1), 271–319. Retrieved from https://gmd.copernicus.org/articles/10/271/2017/ doi: 10.5194/gmd-10-271-2017

Gasser, T., Crepin, L., Quilcaille, Y., Houghton, R. A., Ciais, P., & Obersteiner,

M. (2020). Historical co2 emissions from land use and land cover change and their uncertainty. *Biogeosciences*, *17*(15), 4075–4101. Retrieved from https://bg.copernicus.org/articles/17/4075/2020/ doi: 10.5194/bg-17-4075-2020

Gasser, T., Kechiar, M., Ciais, P., Burke, E. J., Kleinen, T., Zhu, D., ... Obersteiner, M. (2018, Nov 01). Path-dependent reductions in co2 emission budgets caused by permafrost carbon release. *Nature Geoscience*, *11*(11), 830-835. Retrieved from https://doi.org/10.1038/s41561-018-0227-0 doi: 10.1038/s41561-018-0227-0

Gasser, T., Peters, G. P., Fuglestvedt, J. S., Collins, W. J., Shindell, D. T., & Ciais, P. (2017). Accounting for the climate–carbon feedback in emission metrics. *Earth System Dynamics*, *8*(2), 235–253. Retrieved from https://esd.copernicus.org/articles/8/235/2017/ doi: 10.5194/esd-8-235-2017

Geoffroy, O., Saint-Martin, D., Bellon, G., Voldoire, A., Olivié, D. J. L., & Tytéca, S. (2013, 03). Transient Climate Response in a Two-Layer Energy-Balance Model. Part II: Representation of the Efficacy of Deep-Ocean Heat Uptake and Validation for CMIP5 AOGCMs. *Journal of Climate*, *26*(6), 1859-1876. Retrieved from https://doi.org/10.1175/JCLI-D-12-00196.1 doi: 10.1175/JCLI-D-12-00196.1

Geoffroy, O., Saint-Martin, D., Olivié, D. J. L., Voldoire, A., Bellon, G., & Tytéca, S. (2013, mar). Transient climate response in a two-layer energy-balance model. part i: Analytical solution and parameter calibration using CMIP5 AOGCM experiments. *Journal of Climate*, *26*(6), 1841–1857. doi: 10.1175/jcli-d-12-00195.1

Hartin, C. A., Patel, P., Schwarber, A., Link, R. P., & Bond-Lamberty, B. P. (2015, apr). A simple object-oriented and open-source model for scientific and policy analyses of the global climate system – hector v1.0. *Geoscientific Model Development*, *8*(4),

939–955. doi: 10.5194/gmd-8-939-2015

Hauglustaine, D. A., Balkanski, Y., & Schulz, M. (2014). A global model simulation of present and future nitrate aerosols and their direct radiative forcing of climate. *Atmospheric Chemistry and Physics*, *14*(20), 11031–11063. Retrieved from https://acp.copernicus.org/articles/14/11031/2014/ doi: 10.5194/acp-14-11031-2014

Haustein, K., Allen, M. R., Forster, P. M., Otto, F. E. L., Mitchell, D. M., Matthews, H. D., & Frame, D. J. (2017, Nov 13). A real-time global warming index. *Scientific Reports*, *7*(1), 15417. Retrieved from https://doi.org/10.1038/s41598-017-14828-5 doi: 10.1038/s41598-017-14828-5

Hooss, G., Voss, R., Hasselmann, K., Maier-Reimer, E., & Joos, F. (2001, dec). A nonlinear impulse response model of the coupled carbon cycle-climate system (NICCS). *Climate Dynamics*, *18*(3-4), 189–202. doi: 10.1007/s003820100170

Hope, A. P., Canty, T. P., Salawitch, R. J., Tribett, W. R., & Bennett, B. F. (2017). Forecasting global warming [Book Section]. In *Paris climate agreement: Beacon of hope* (p. 51-114). Springer Climate.

Hope, A. P., McBride, L. A., Canty, T. P., Bennett, B. F., Tribett, W. R., & Salawitch, R. J. (2020). Examining the human influence on global climate using an empirical model. *Earth and Space Science Open Archive*, 79. Retrieved from https://doi.org/10.1002/essoar.10504179.1 doi: 10.1002/essoar.10504179.1

IPCC. (2013). Summary for policymakers [Book Section]. In T. Stocker et al. (Eds.), *Climate change 2013: The physical science basis. contribution of working group i to the fifth assessment report of the intergovernmental panel on climate*

change (p. 1–30). Cambridge, United Kingdom and New York, NY, USA: Cambridge University Press. Retrieved from http://www.climatechange2013.org doi: 10.1017/CBO9781107415324.004

Joos, F., Bruno, M., Fink, R., Siegenthaler, U., Stocker, T. F., Quéré, C. L., & Sarmiento, J. L. (1996). An efficient and accurate representation of complex oceanic and biospheric models of anthropogenic carbon uptake. *Tellus B: Chemical and Physical Meteorology*, *48*(3), 394-417. Retrieved from https://doi.org/10.3402/tellusb.v48i3.15921 doi: 10.3402/tellusb.v48i3.15921

Kriegler, E. (2005). *Imprecise probability analysis for integrated assessment of climate change* (Doctoral thesis, Universität Potsdam). Retrieved from https://publishup.uni-potsdam.de/opus4-ubp/frontdoor/index/index/docId/497

Leach, N. J., Jenkins, S., Nicholls, Z., Smith, C. J., Lynch, J., Cain, M., ... Allen, M. R. (2020). Fairv2.0.0: a generalised impulse-response model for climate uncertainty and future scenario exploration. *Geoscientific Model Development Discussions*, *2020*, 1–48. Retrieved from https://gmd.copernicus.org/preprints/gmd-2020-390/ doi: 10.5194/gmd-2020-390

McBride, L. A., Hope, A. P., Canty, T. P., Bennett, B. F., Tribett, W. R., & Salawitch, R. J. (2020). Comparison of cmip6 historical climate simulations and future projected warming to an empirical model of global climate. *Earth System Dynamics Discussions*, *2020*, 1–59. Retrieved from https://esd.copernicus.org/preprints/esd-2020-67/ doi: 10.5194/esd-2020-67

Meinshausen, M., Meinshausen, N., Hare, W., Raper, S. C. B., Frieler, K., Knutti, R., ... Allen, M. R. (2009, Apr 01). Greenhouse-gas emission targets for limiting global

warming to 2 °c. *Nature*, *458*(7242), 1158-1162. Retrieved from https://doi.org/10.1038/nature08017 doi: 10.1038/nature08017

Meinshausen, M., Nicholls, Z. R. J., Lewis, J., Gidden, M. J., Vogel, E., Freund, M., ... Wang, R. H. J. (2020). The shared socio-economic pathway (ssp) greenhouse gas concentrations and their extensions to 2500. *Geoscientific Model Development*, *13*(8), 3571–3605. Retrieved from https://gmd.copernicus.org/articles/13/3571/2020/ doi: 10.5194/gmd-13-3571-2020

Meinshausen, M., Raper, S. C. B., & Wigley, T. M. L. (2011). Emulating coupled atmosphere-ocean and carbon cycle models with a simpler model, MAGICC6 – part 1: Model description and calibration. *Atmospheric Chemistry and Physics*, *11*(4), 1417–1456. doi: 10.5194/acp-11-1417-2011

Millar, R. J., Nicholls, Z. R., Friedlingstein, P., & Allen, M. R. (2017, jun). A modified impulse-response representation of the global near-surface air temperature and atmospheric concentration response to carbon dioxide emissions. *Atmospheric Chemistry and Physics*, *17*(11), 7213–7228. doi: 10.5194/acp-17-7213-2017

Morice, C. P., Kennedy, J. J., Rayner, N. A., & Jones, P. D. (2012, apr). Quantifying uncertainties in global and regional temperature change using an ensemble of observational estimates: The HadCRUT4 data set. *Journal of Geophysical Research: Atmospheres*, *117*(D8), n/a–n/a. doi: 10.1029/2011jd017187

Morice, C. P., Kennedy, J. J., Rayner, N. A., Winn, J. P., Hogan, E., Killick, R. E., ... Simpson, I. R. (2021). An updated assessment of near-surface temperature change from 1850: The hadcrut5 data set. *Journal of Geophysical Research: Atmospheres*, *126*(3), e2019JD032361. Retrieved from https://agupubs.onlinelibrary

.wiley.com/doi/abs/10.1029/2019JD032361 (e2019JD032361 2019JD032361) doi: https://doi.org/10.1029/2019JD032361

Myhre, G., Shindell, D., Bréon, F.-M., Collins, W., Fuglestvedt, J., Huang, J., ... Zhang, H. (2013). Anthropogenic and natural radiative forcing [Book Section]. In T. Stocker et al. (Eds.), *Climate change 2013: The physical science basis. contribution of working group i to the fifth assessment report of the intergovernmental panel on climate change* (p. 659–740). Cambridge, United Kingdom and New York, NY, USA: Cambridge University Press. Retrieved from http://www.climatechange2013.org doi: 10.1017/CBO9781107415324.018

Nicholls, Z. R. J., Meinshausen, M., Lewis, J., Gieseke, R., Dommenget, D., Dorheim, K., ... Xie, Z. (2020). Reduced complexity model intercomparison project phase 1: introduction and evaluation of global-mean temperature response. *Geoscientific Model Development*, *13*(11), 5175–5190. Retrieved from https://gmd.copernicus.org/articles/13/5175/2020/ doi: 10.5194/gmd-13-5175-2020

O'Neill, B. C., Tebaldi, C., van Vuuren, D. P., Eyring, V., Friedlingstein, P., Hurtt, G., ... Sanderson, B. M. (2016). The scenario model intercomparison project (scenariomip) for cmip6. *Geoscientific Model Development*, *9*(9), 3461–3482. Retrieved from https://gmd.copernicus.org/articles/9/3461/2016/ doi: 10.5194/gmd-9-3461-2016

Schlesinger, M. E., Jiang, X., & Charlson, R. J. (1992). Implication of anthropogenic atmospheric sulphate for the sensitivity of the climate system. In *Climate change and energy policy: Proceedings of the international conference on global climate change: Its mitigation through improved production and use of energy [rosen, l. and r. glasser*

(eds.)]. amer. inst. phys., new york, ny, usa (pp. 75–108).

Skeie, R. B., Berntsen, T., Aldrin, M., Holden, M., & Myhre, G. (2018, jun). Climate sensitivity estimates – sensitivity to radiative forcing time series and observational data. *Earth System Dynamics*, *9*(2), 879–894. doi: 10.5194/esd-9-879-2018

Skeie, R. B., Fuglestvedt, J., Berntsen, T., Peters, G. P., Andrew, R., Allen, M., & Kallbekken, S. (2017, feb). Perspective has a strong effect on the calculation of historical contributions to global warming. *Environmental Research Letters*, *12*(2), 024022. doi: 10.1088/1748-9326/aa5b0a

Smith, C. J., Forster, P. M., Allen, M., Leach, N., Millar, R. J., Passerello, G. A., & Regayre, L. A. (2018, jun). FAIR v1.3: a simple emissions-based impulse response and carbon cycle model. *Geoscientific Model Development*, *11*(6), 2273–2297. doi: 10.5194/gmd-11-2273-2018

Smith, C. J., et al. (submitted).

JGR: Atmospheres.

Su, X., Shiogama, H., Tanaka, K., Fujimori, S., Hasegawa, T., Hijioka, Y., ... Liu, J. (2018). How do climate-related uncertainties influence 2 and 1.5° c pathways? *Sustainability science*, *13*(2), 291–299.

Su, X., Tachiiri, K., Tanaka, K., Watanabe, M., & Kawamiya, M. (2020). Source attributions of radiative forcing by regions, sectors, and climate forcers. *arXiv preprint arXiv:2009.07472*.

Su, X., Takahashi, K., Fujimori, S., Hasegawa, T., Tanaka, K., Kato, E., ... Emori, S. (2017). Emission pathways to achieve 2.0 c and 1.5 c climate targets. *Earth's Future*, *5*(6), 592–604.

Tanaka, K., Kriegler, E., Bruckner, T., Hooss, G., Knorr, W., Raddatz, T., & Tol, R. (2007). *Aggregated carbon cycle, atmospheric chemistry and climate model (acc2): description of forward and inverse mode.* Retrieved from https://pure.mpg.de/rest/items/item_994422/component/file_994421/content

Tsutsui, J. (2017). Quantification of temperature response to CO2 forcing in atmosphere–ocean general circulation models. *Climatic Change*, *140*(2), 287–305. doi: 10.1007/s10584-016-1832-9

Tsutsui, J. (2020). Diagnosing transient response to CO2 forcing in coupled atmosphere-ocean model experiments using a climate model emulator. *Geophysical Research Letters*, *47*(7). doi: 10.1029/2019gl085844

Tsutsui, J. (2021). *tsutsui1872/mce: Minimal CMIP Emulator (MCE v1.2).* Zenodo. Retrieved from https://doi.org/10.5281/zenodo.4604695 doi: 10.5281/zenodo.4604695

Urban, N. M., Holden, P. B., Edwards, N. R., Sriver, R. L., & Keller, K. (2014). Historical and future learning about climate sensitivity. *Geophysical Research Letters*, *41*(7), 2543-2552. Retrieved from https://agupubs.onlinelibrary.wiley.com/doi/abs/10.1002/2014GL059484 doi: 10.1002/2014GL059484

Vega-Westhoff, B., Sriver, R. L., Hartin, C. A., Wong, T. E., & Keller, K. (2019, jun). Impacts of observational constraints related to sea level on estimates of climate sensitivity. *Earth's Future*, *7*(6), 677–690. doi: 10.1029/2018ef001082

von Schuckmann, K., Cheng, L., Palmer, M. D., Hansen, J., Tassone, C., Aich, V., ... Wijffels, S. E. (2020). Heat stored in the earth system: where does the energy go? *Earth System Science Data*, *12*(3), 2013–2041. Retrieved from https://essd

.copernicus.org/articles/12/2013/2020/ doi: 10.5194/essd-12-2013-2020

Table S1. Comparison of each model's probabilistic distribution with the proxy assessment. The assessed ranges are labelled as 'vll' (very-likely lower i.e. 5th percentile), 'll' (likely lower, 17th percentile), 'c' (central, 50th percentile), 'lu' (likely upper, 83th percentile) and 'vlu' (very-likely upper, 95th percentile).

Metric	Source<br>Assessed range<br>Unit	Assessed ranges vll	ll	c	lu	vlu	CICERO-SCM vll	ll	c	lu	vlu
2000-2019 GMST rel. to 1961-1990	K	0.46		0.54		0.61	0.49		0.60		0.72
Equilibrium Climate Sensitivity	K	2.30	2.60	3.10	3.90	4.70	2.52	2.69	3.03	3.43	3.67
Transient Climate Response	K	0.98	1.26	1.64	2.02	2.29	1.31	1.44	1.66	1.91	2.03
Transient Climate Response to Emissions	K / TtC	1.03	1.40	1.77	2.14	2.51					
2014 CO_2 Effective Radiative Forcing	W / m^2		1.69	1.80	1.91			1.94	1.94	1.94	
2014 Aerosol Effective Radiative Forcing	W / m^2		-1.37	-1.01	-0.63			-0.99	-0.64	-0.23	
2018 Ocean Heat Content rel. to 1971	ZJ		303	320	337			303	320	337	
2011 CH_4 Effective Radiative Forcing	W / m^2		0.47	0.60	0.73			0.53	0.53	0.53	
2011 N_2O Effective Radiative Forcing	W / m^2		0.14	0.17	0.20			0.16	0.16	0.16	
2011 F-Gases Effective Radiative Forcing	W / m^2		0.03	0.03	0.03						

Table S1 (cont.). Comparison of each model's probabilistic distribution with the proxy assessment. The assessed ranges are labelled as 'vll' (very-likely lower i.e. 5th percentile), 'll' (likely lower, 17th percentile), 'c' (central, 50th percentile), 'lu' (likely upper, 83th percentile) and 'vlu' (very-likely upper, 95th percentile).

	Source	Assessed ranges					EMGC				
	Assessed range	vll	ll	c	lu	vlu	vll	ll	c	lu	vlu
Metric	Unit										
2000-2019 GMST rel. to 1961-1990	K	0.46		0.54		0.61	0.32		0.48		0.83
Equilibrium Climate Sensitivity	K	2.30	2.60	3.10	3.90	4.70	1.30	1.50	1.93	2.79	4.14
Transient Climate Response	K	0.98	1.26	1.64	2.02	2.29					
Transient Climate Response to Emissions	K / TtC	1.03	1.40	1.77	2.14	2.51					
2014 CO_2 Effective Radiative Forcing	W / m^2		1.69	1.80	1.91			1.90	1.90	1.90	
2014 Aerosol Effective Radiative Forcing	W / m^2		-1.37	-1.01	-0.63			-1.16	-0.84	-0.53	
2018 Ocean Heat Content rel. to 1971	ZJ		303	320	337			277	327	426	
2011 CH_4 Effective Radiative Forcing	W / m^2		0.47	0.60	0.73			0.58	0.58	0.58	
2011 N_2O Effective Radiative Forcing	W / m^2		0.14	0.17	0.20			0.18	0.18	0.18	
2011 F-Gases Effective Radiative Forcing	W / m^2		0.03	0.03	0.03						

Table S1 (cont.). Comparison of each model's probabilistic distribution with the proxy assessment. The assessed ranges are labelled as 'vll' (very-likely lower i.e. 5th percentile), 'll' (likely lower, 17th percentile), 'c' (central, 50th percentile), 'lu' (likely upper, 83th percentile) and 'vlu' (very-likely upper, 95th percentile).

	Source	Assessed ranges					FaIR1.6				
	Assessed range	vll	ll	c	lu	vlu	vll	ll	c	lu	vlu
Metric	Unit										
2000-2019 GMST rel. to 1961-1990	K	0.46		0.54		0.61	0.53		0.66		0.84
Equilibrium Climate Sensitivity	K	2.30	2.60	3.10	3.90	4.70	1.93	2.33	3.01	4.33	6.21
Transient Climate Response	K	0.98	1.26	1.64	2.02	2.29	1.40	1.55	1.80	2.12	2.48
Transient Climate Response to Emissions	K / TtC	1.03	1.40	1.77	2.14	2.51	1.21	1.36	1.59	1.91	2.22
2014 CO_2 Effective Radiative Forcing	W / m^2		1.69	1.80	1.91			1.65	1.89	2.17	
2014 Aerosol Effective Radiative Forcing	W / m^2		-1.37	-1.01	-0.63			-1.34	-1.01	-0.65	
2018 Ocean Heat Content rel. to 1971	ZJ		303	320	337			303	359	417	
2011 CH_4 Effective Radiative Forcing	W / m^2		0.47	0.60	0.73			0.49	0.55	0.62	
2011 N_2O Effective Radiative Forcing	W / m^2		0.14	0.17	0.20			0.15	0.17	0.18	
2011 F-Gases Effective Radiative Forcing	W / m^2		0.03	0.03	0.03			0.03	0.03	0.03	

Table S1 (cont.). Comparison of each model's probabilistic distribution with the proxy assessment. The assessed ranges are labelled as 'vll' (very-likely lower i.e. 5th percentile), 'll' (likely lower, 17th percentile), 'c' (central, 50th percentile), 'lu' (likely upper, 83th percentile) and 'vlu' (very-likely upper, 95th percentile).

	Source	Assessed ranges					FaIRv2.0.0-alpha				
	Assessed range	vll	ll	c	lu	vlu	vll	ll	c	lu	vlu
Metric	Unit										
2000-2019 GMST rel. to 1961-1990	K	0.46		0.54		0.61	0.51		0.66		0.85
Equilibrium Climate Sensitivity	K	2.30	2.60	3.10	3.90	4.70	1.80	2.20	3.05	4.50	6.17
Transient Climate Response	K	0.98	1.26	1.64	2.02	2.29	1.23	1.41	1.71	2.08	2.38
Transient Climate Response to Emissions	K / TtC	1.03	1.40	1.77	2.14	2.51	1.02	1.17	1.41	1.74	2.01
2014 CO_2 Effective Radiative Forcing	W / m^2		1.69	1.80	1.91			1.73	1.95	2.18	
2014 Aerosol Effective Radiative Forcing	W / m^2		-1.37	-1.01	-0.63			-1.53	-1.14	-0.80	
2018 Ocean Heat Content rel. to 1971	ZJ		303	320	337						
2011 CH_4 Effective Radiative Forcing	W / m^2		0.47	0.60	0.73			0.50	0.59	0.69	
2011 N_2O Effective Radiative Forcing	W / m^2		0.14	0.17	0.20			0.15	0.17	0.19	
2011 F-Gases Effective Radiative Forcing	W / m^2		0.03	0.03	0.03			0.03	0.03	0.04	

Table S1 (cont.). Comparison of each model's probabilistic distribution with the proxy assessment. The assessed ranges are labelled as 'vll' (very-likely lower i.e. 5th percentile), 'll' (likely lower, 17th percentile), 'c' (central, 50th percentile), 'lu' (likely upper, 83th percentile) and 'vlu' (very-likely upper, 95th percentile).

	Source	Assessed ranges					Hector				
	Assessed range	vll	ll	c	lu	vlu	vll	ll	c	lu	vlu
Metric	Unit										
2000-2019 GMST rel. to 1961-1990	K	0.46		0.54		0.61	0.50		0.62		0.77
Equilibrium Climate Sensitivity	K	2.30	2.60	3.10	3.90	4.70	1.83	2.17	2.86	3.91	5.44
Transient Climate Response	K	0.98	1.26	1.64	2.02	2.29	1.42	1.58	1.81	2.07	2.30
Transient Climate Response to Emissions	K / TtC	1.03	1.40	1.77	2.14	2.51					
2014 CO_2 Effective Radiative Forcing	W / m^2		1.69	1.80	1.91						
2014 Aerosol Effective Radiative Forcing	W / m^2		-1.37	-1.01	-0.63			-0.68	-0.57	-0.45	
2018 Ocean Heat Content rel. to 1971	ZJ		303	320	337						
2011 CH_4 Effective Radiative Forcing	W / m^2		0.47	0.60	0.73						
2011 N_2O Effective Radiative Forcing	W / m^2		0.14	0.17	0.20						
2011 F-Gases Effective Radiative Forcing	W / m^2		0.03	0.03	0.03						

Table S1 (cont.). Comparison of each model's probabilistic distribution with the proxy assessment. The assessed ranges are labelled as 'vll' (very-likely lower i.e. 5th percentile), 'll' (likely lower, 17th percentile), 'c' (central, 50th percentile), 'lu' (likely upper, 83th percentile) and 'vlu' (very-likely upper, 95th percentile).

	Source	Assessed ranges					MAGICCv7.5.1				
	Assessed range	vll	ll	c	lu	vlu	vll	ll	c	lu	vlu
Metric	Unit										
2000-2019 GMST rel. to 1961-1990	K	0.46		0.54		0.61	0.47		0.54		0.63
Equilibrium Climate Sensitivity	K	2.30	2.60	3.10	3.90	4.70	2.00	2.20	2.66	3.27	3.90
Transient Climate Response	K	0.98	1.26	1.64	2.02	2.29	1.30	1.46	1.76	2.07	2.30
Transient Climate Response to Emissions	K / TtC	1.03	1.40	1.77	2.14	2.51	1.13	1.38	1.75	2.17	2.47
2014 CO_2 Effective Radiative Forcing	W / m^2		1.69	1.80	1.91			1.66	1.78	1.89	
2014 Aerosol Effective Radiative Forcing	W / m^2		-1.37	-1.01	-0.63			-1.39	-1.08	-0.75	
2018 Ocean Heat Content rel. to 1971	ZJ		303	320	337			305	323	340	
2011 CH_4 Effective Radiative Forcing	W / m^2		0.47	0.60	0.73			0.47	0.58	0.71	
2011 N_2O Effective Radiative Forcing	W / m^2		0.14	0.17	0.20			0.14	0.17	0.20	
2011 F-Gases Effective Radiative Forcing	W / m^2		0.03	0.03	0.03			0.03	0.03	0.03	

Table S1 (cont.). Comparison of each model's probabilistic distribution with the proxy assessment. The assessed ranges are labelled as 'vll' (very-likely lower i.e. 5th percentile), 'll' (likely lower, 17th percentile), 'c' (central, 50th percentile), 'lu' (likely upper, 83th percentile) and 'vlu' (very-likely upper, 95th percentile).

	Source	Assessed ranges					MCE-v1-2				
	Assessed range	vll	ll	c	lu	vlu	vll	ll	c	lu	vlu
Metric	Unit										
2000-2019 GMST rel. to 1961-1990	K	0.46		0.54		0.61	0.46		0.55		0.63
Equilibrium Climate Sensitivity	K	2.30	2.60	3.10	3.90	4.70	1.74	2.02	2.42	3.01	3.50
Transient Climate Response	K	0.98	1.26	1.64	2.02	2.29	1.20	1.31	1.54	1.77	1.95
Transient Climate Response to Emissions	K / TtC	1.03	1.40	1.77	2.14	2.51	1.03	1.15	1.36	1.64	1.84
2014 CO_2 Effective Radiative Forcing	W / m^2		1.69	1.80	1.91			1.67	1.79	1.93	
2014 Aerosol Effective Radiative Forcing	W / m^2		-1.37	-1.01	-0.63			-1.23	-0.95	-0.69	
2018 Ocean Heat Content rel. to 1971	ZJ		303	320	337			299	319	340	
2011 CH_4 Effective Radiative Forcing	W / m^2		0.47	0.60	0.73			0.47	0.58	0.70	
2011 N_2O Effective Radiative Forcing	W / m^2		0.14	0.17	0.20			0.14	0.17	0.20	
2011 F-Gases Effective Radiative Forcing	W / m^2		0.03	0.03	0.03			0.03	0.03	0.03	

Table S1 (cont.). Comparison of each model's probabilistic distribution with the proxy assessment. The assessed ranges are labelled as 'vll' (very-likely lower i.e. 5th percentile), 'll' (likely lower, 17th percentile), 'c' (central, 50th percentile), 'lu' (likely upper, 83th percentile) and 'vlu' (very-likely upper, 95th percentile).

	Source	Assessed ranges					OSCARv3.1				
	Assessed range	vll	ll	c	lu	vlu	vll	ll	c	lu	vlu
Metric	Unit										
2000-2019 GMST rel. to 1961-1990	K	0.46		0.54		0.61	0.48		0.55		0.62
Equilibrium Climate Sensitivity	K	2.30	2.60	3.10	3.90	4.70	2.36	2.37	2.63	3.34	3.75
Transient Climate Response	K	0.98	1.26	1.64	2.02	2.29	1.41	1.52	1.62	1.81	1.96
Transient Climate Response to Emissions	K / TtC	1.03	1.40	1.77	2.14	2.51	1.15	1.25	1.41	1.62	1.82
2014 CO_2 Effective Radiative Forcing	W / m^2		1.69	1.80	1.91			1.91	1.91	1.91	
2014 Aerosol Effective Radiative Forcing	W / m^2		-1.37	-1.01	-0.63			-1.65	-1.11	-0.64	
2018 Ocean Heat Content rel. to 1971	ZJ		303	320	337			241	335	432	
2011 CH_4 Effective Radiative Forcing	W / m^2		0.47	0.60	0.73			0.50	0.50	0.50	
2011 N_2O Effective Radiative Forcing	W / m^2		0.14	0.17	0.20			0.18	0.18	0.18	
2011 F-Gases Effective Radiative Forcing	W / m^2		0.03	0.03	0.03			0.03	0.03	0.03	

Table S1 (cont.). Comparison of each model's probabilistic distribution with the proxy assessment. The assessed ranges are labelled as 'vll' (very-likely lower i.e. 5th percentile), 'll' (likely lower, 17th percentile), 'c' (central, 50th percentile), 'lu' (likely upper, 83th percentile) and 'vlu' (very-likely upper, 95th percentile).

	Source	Assessed ranges					SCM4OPTv2.1				
	Assessed range	vll	ll	c	lu	vlu	vll	ll	c	lu	vlu
Metric	Unit										
2000-2019 GMST rel. to 1961-1990	K	0.46		0.54		0.61	0.43		0.59		0.79
Equilibrium Climate Sensitivity	K	2.30	2.60	3.10	3.90	4.70	2.60	2.71	3.47	4.11	4.58
Transient Climate Response	K	0.98	1.26	1.64	2.02	2.29	1.57	1.69	1.78	2.03	2.16
Transient Climate Response to Emissions	K / TtC	1.03	1.40	1.77	2.14	2.51					
2014 CO_2 Effective Radiative Forcing	W / m^2		1.69	1.80	1.91			1.80	1.80	1.80	
2014 Aerosol Effective Radiative Forcing	W / m^2		-1.37	-1.01	-0.63			-1.56	-1.11	-0.78	
2018 Ocean Heat Content rel. to 1971	ZJ		303	320	337			241	321	392	
2011 CH_4 Effective Radiative Forcing	W / m^2		0.47	0.60	0.73			0.48	0.48	0.48	
2011 N_2O Effective Radiative Forcing	W / m^2		0.14	0.17	0.20			0.17	0.17	0.17	
2011 F-Gases Effective Radiative Forcing	W / m^2		0.03	0.03	0.03			0.03	0.03	0.03	

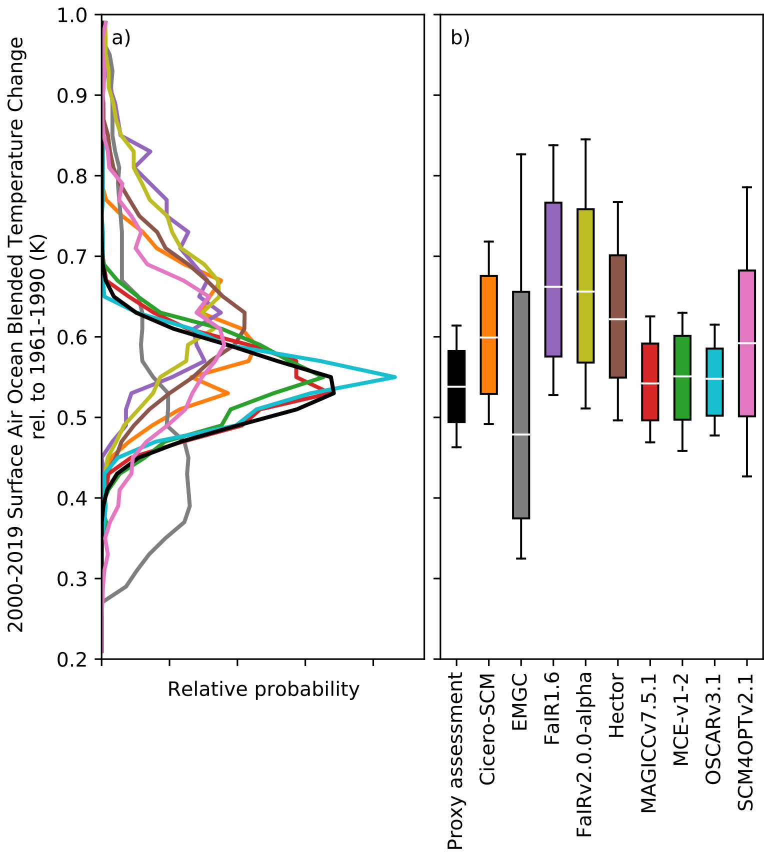

Figure S1. As in Figure 1, except for 2000-2019 mean global-mean surface temperature (GMST) change relative to 1961-1990.

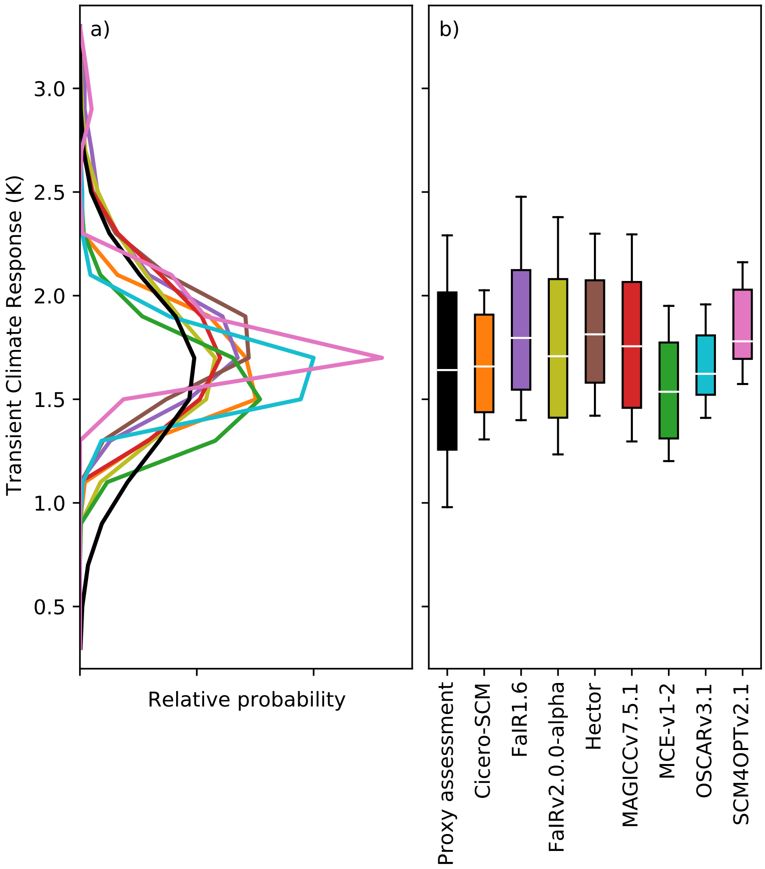

Figure S2. As in Figure 1, except for TCR.

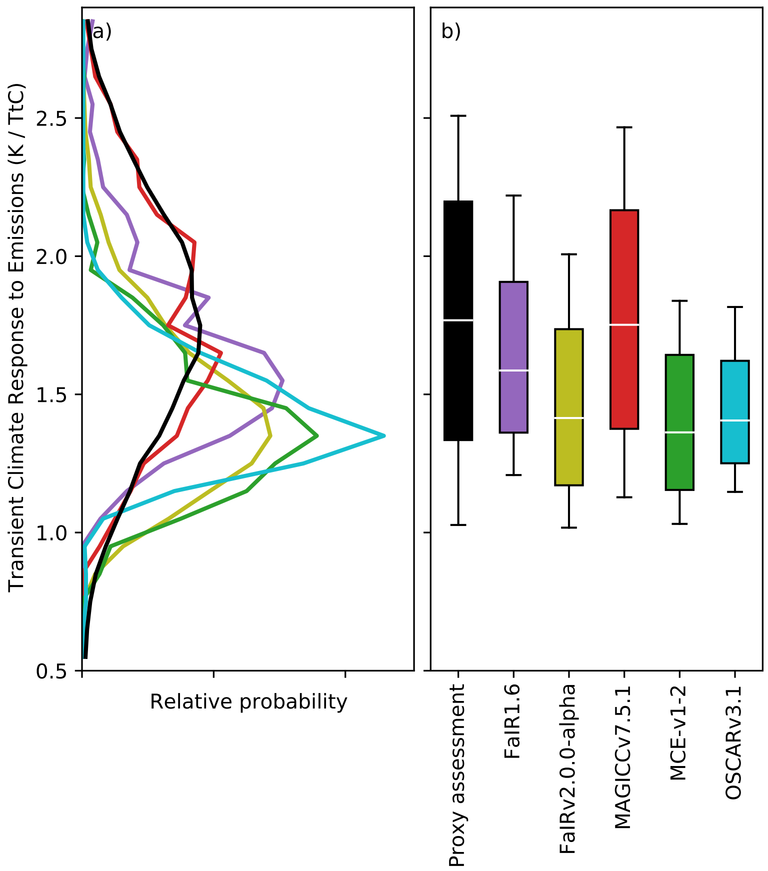

Figure S3. As in Figure 1, except for TCRE.

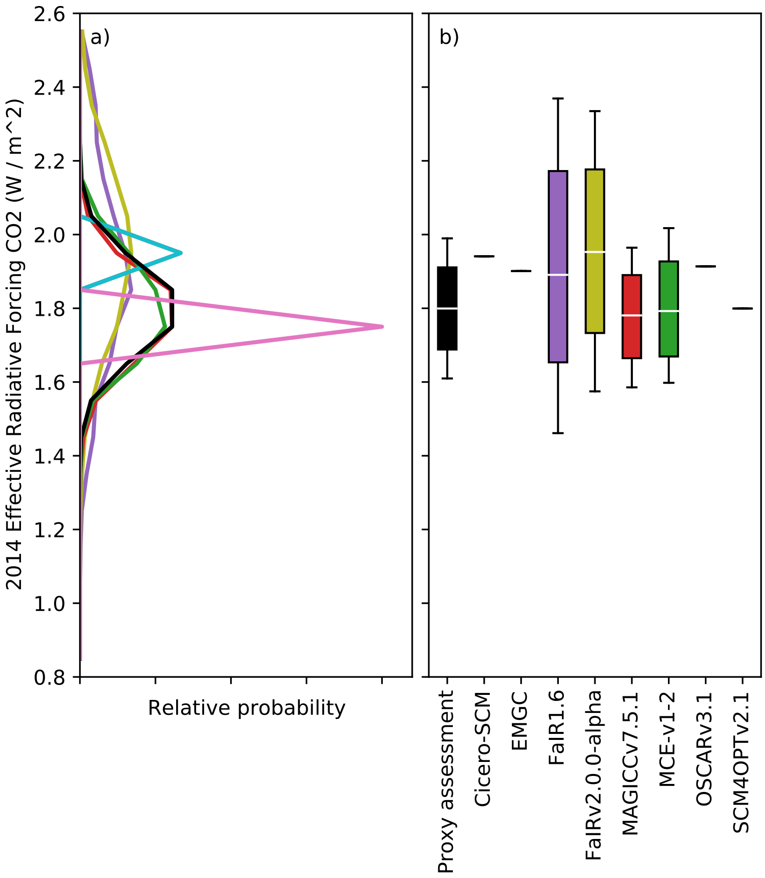

Figure S4. As in Figure 1, except for 2014 CO_2 effective radiative forcing.

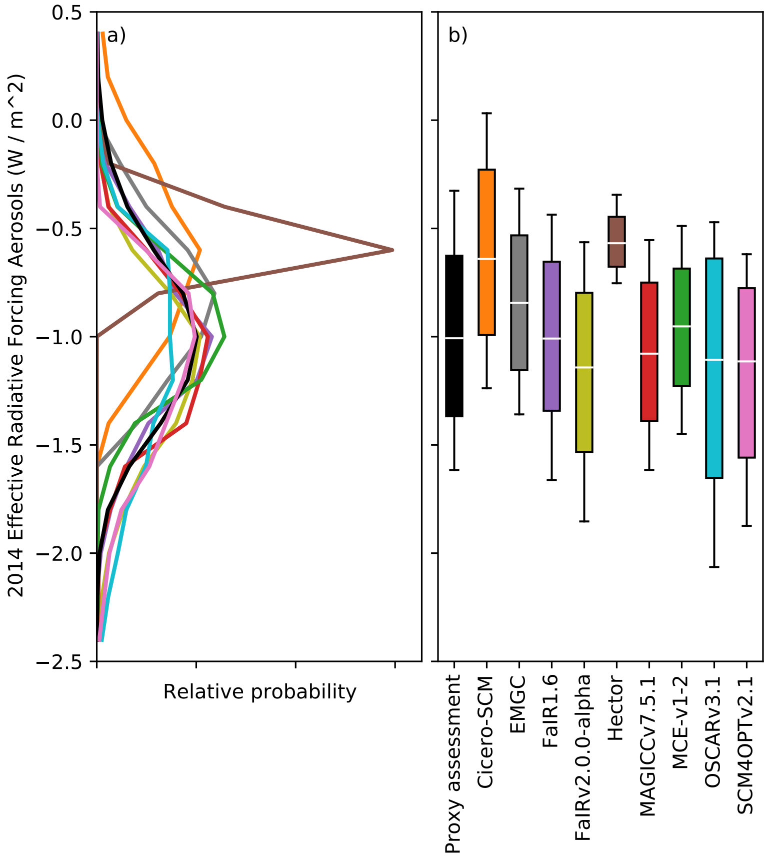

Figure S5. As in Figure 1, except for 2014 aerosol effective radiative forcing.

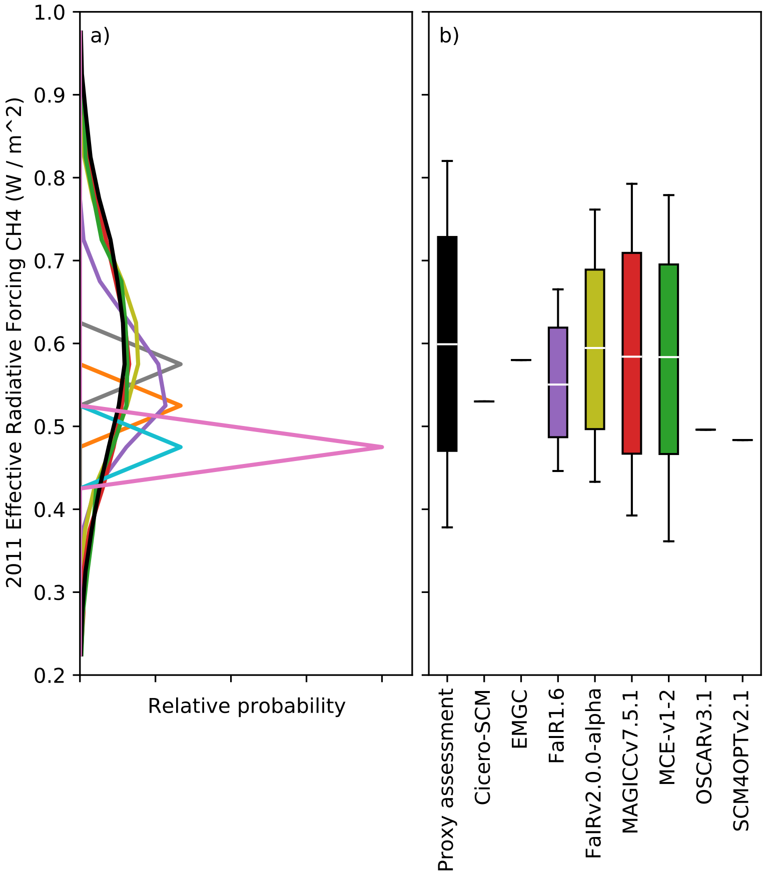

Figure S6. As in Figure 1, except for 2011 CH_4 effective radiative forcing.

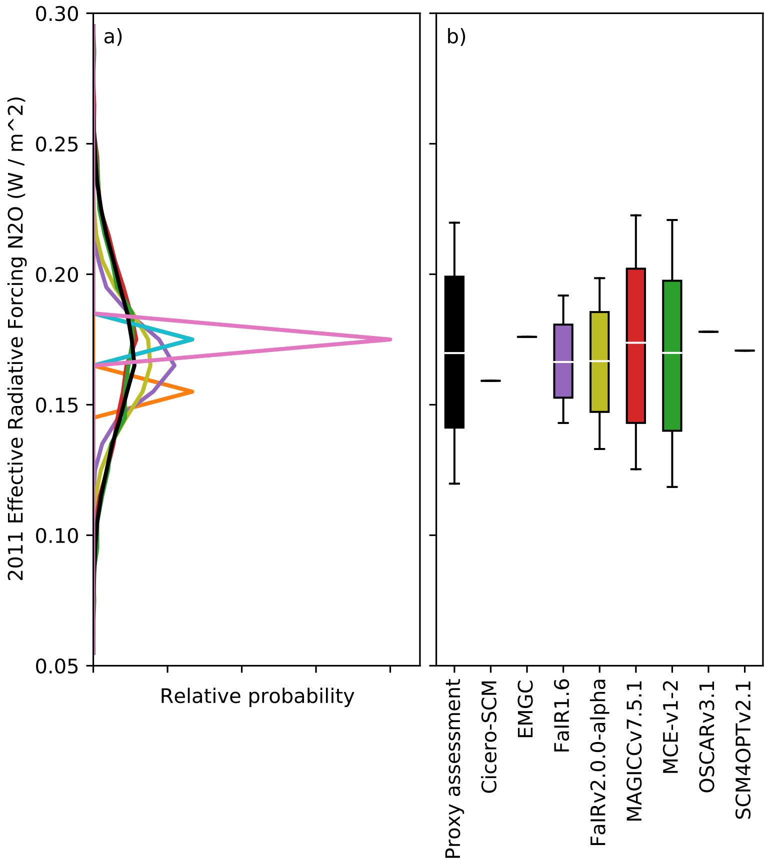

Figure S7. As in Figure 1, except for 2011 N_2O effective radiative forcing.

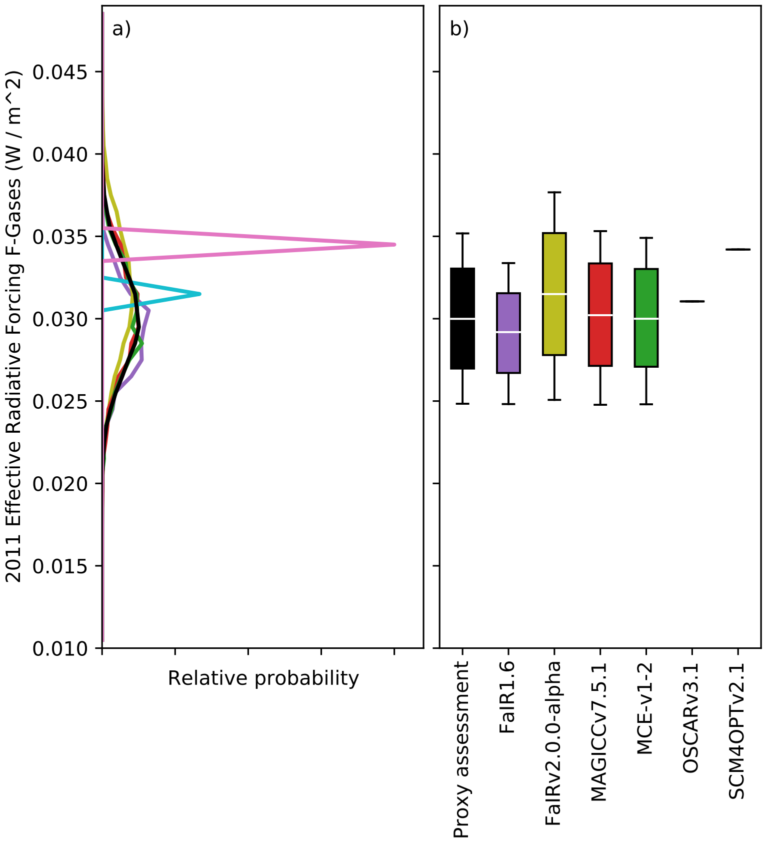

Figure S8. As in Figure 1, except for 2011 F-Gases effective radiative forcing.

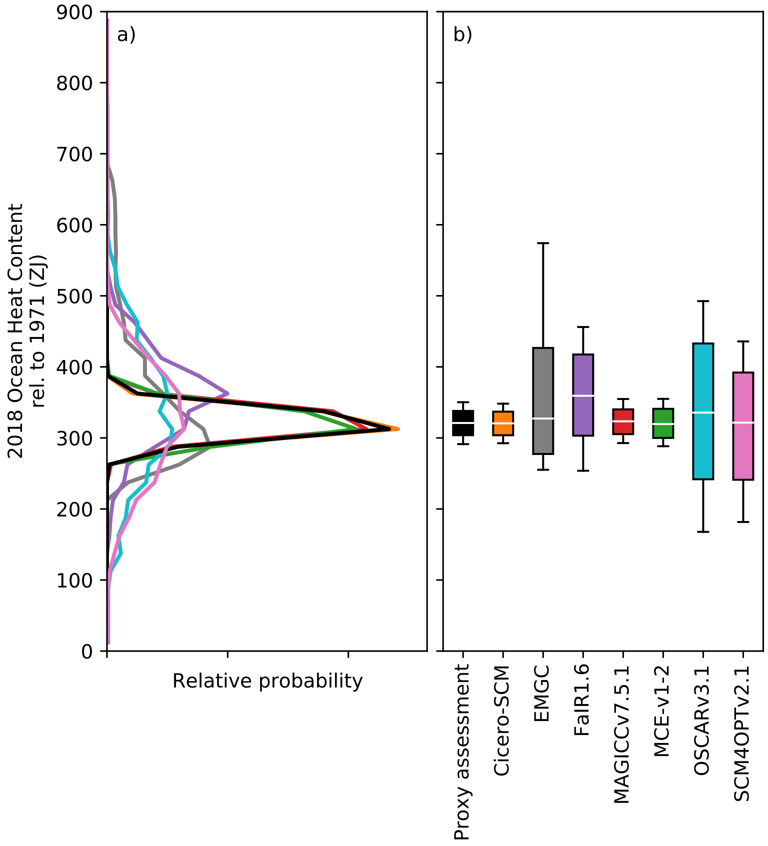

Figure S9. As in Figure 1, except for 2018 ocean heat content change relative to 1971.

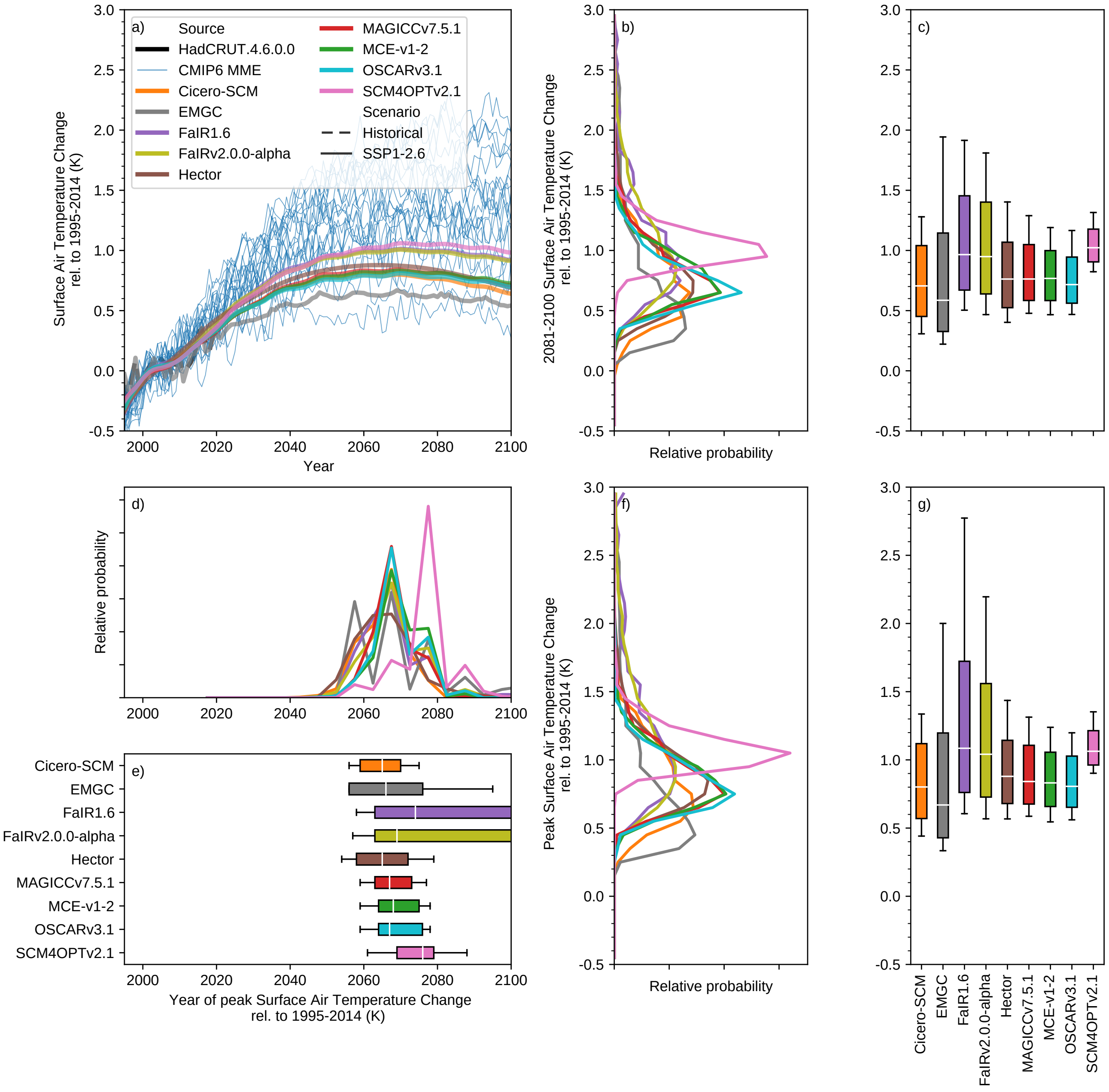

Figure S10. As in Figure 1 except for the low-emissions SSP1-2.6 scenario.

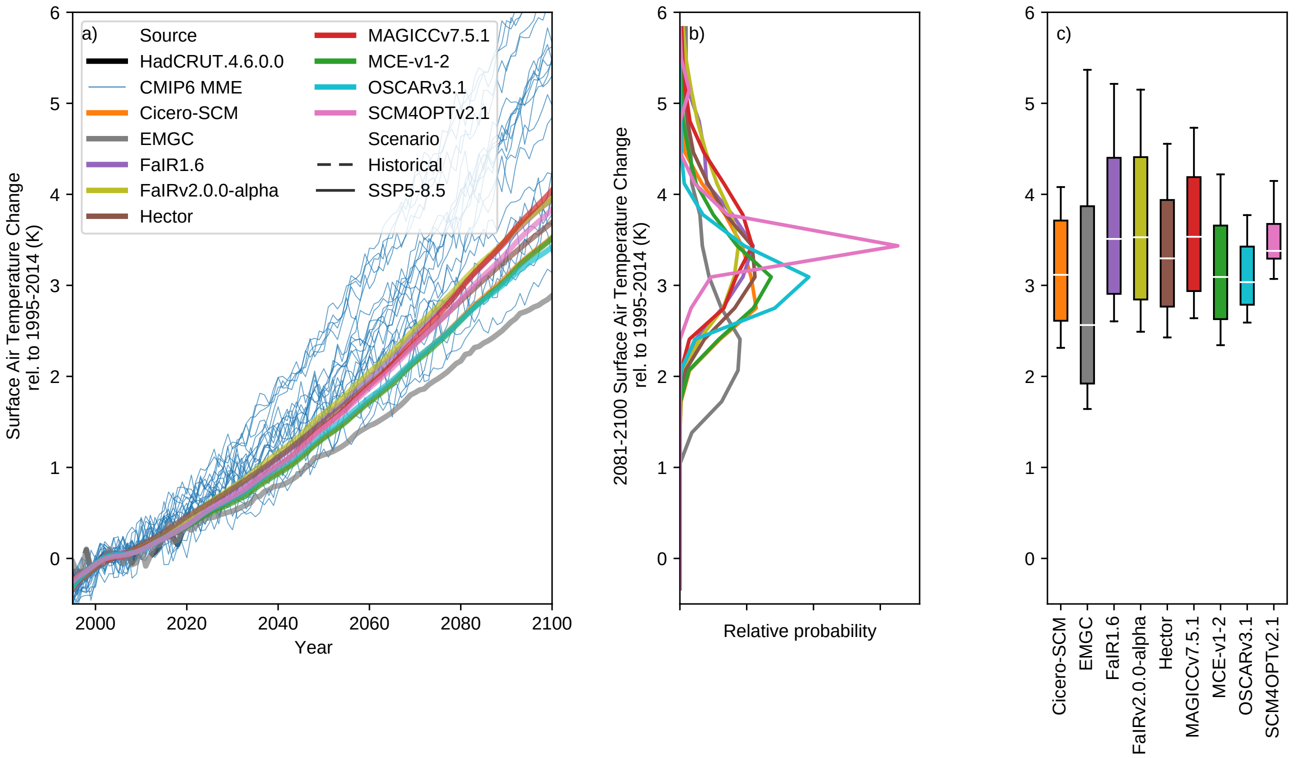

Figure S11. As in panels a), b) and c) of Figure 1 except for the high-emissions SSP5-8.5 scenario.

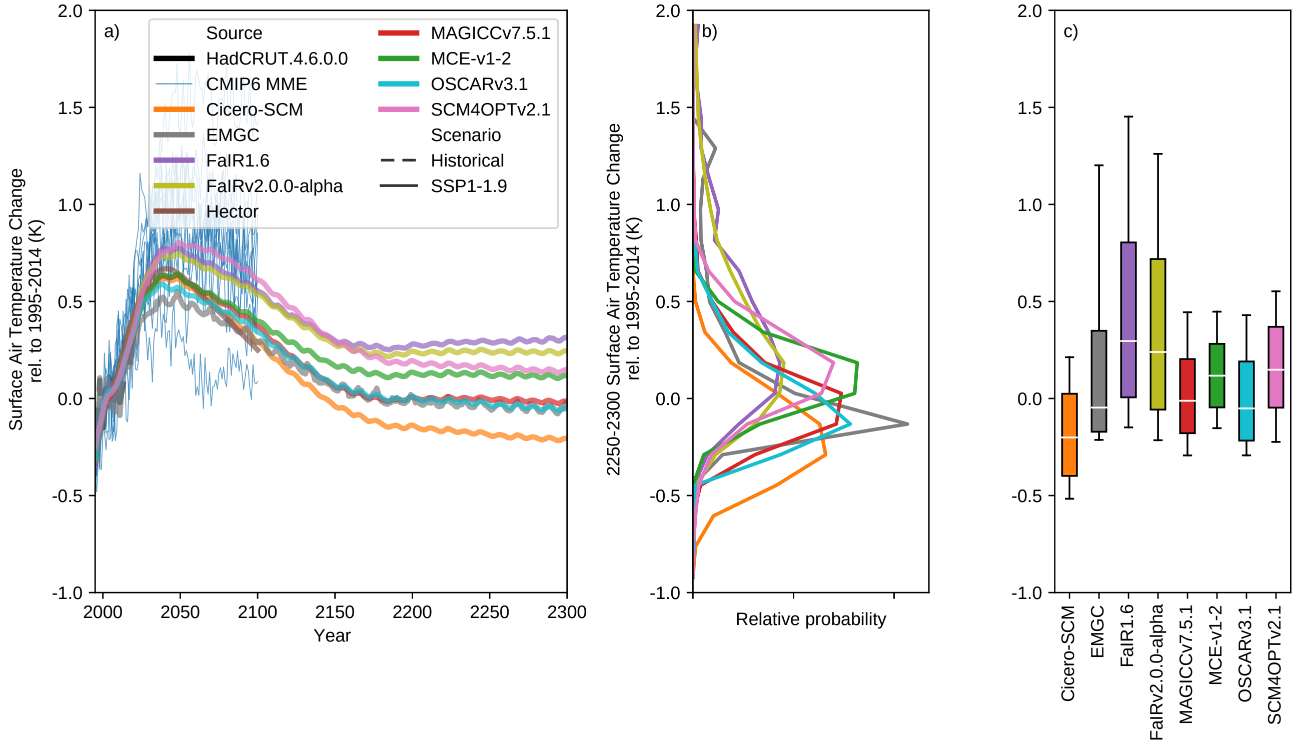

Figure S12. As in Figure 2 except for the low-emissions SSP1-1.9 scenario.

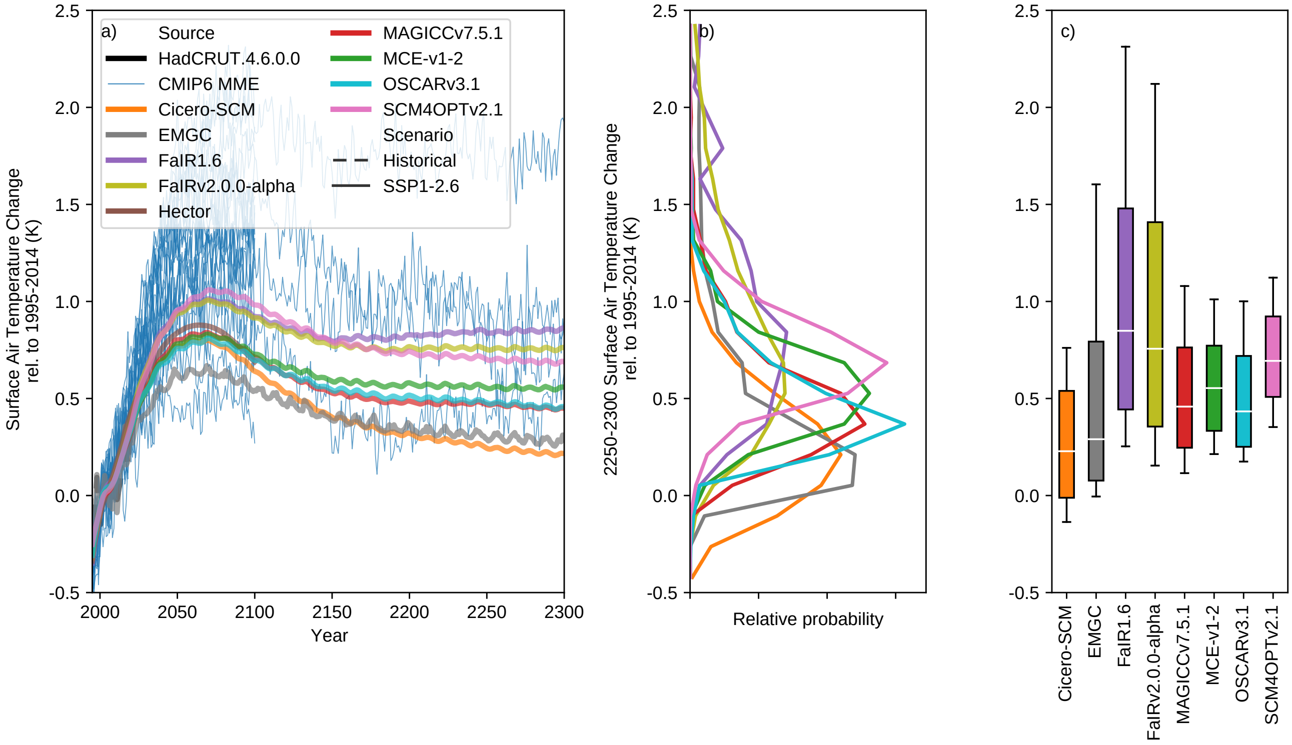

Figure S13. As in Figure 2 except for the low-emissions SSP1-2.6 scenario.

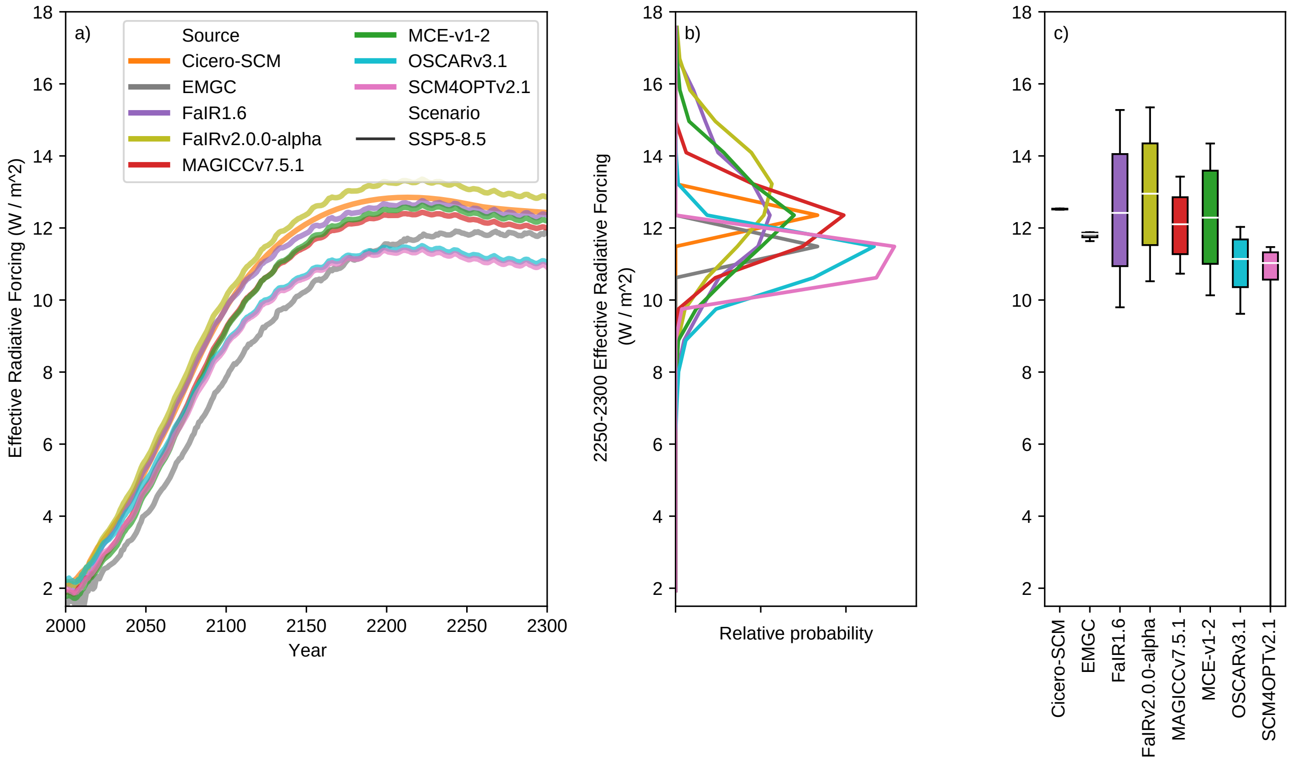

Figure S14. Long-term effective radiative forcing under the high emissions SSP5-8.5 scenario. a) Effective radiative forcing projections from 1995 to 2300 for each RCM; b) distribution of 2250-2300 mean effective radiative forcing from each RCM; c) very likely (whiskers), likely (box) and central (white line) 2250-2300 mean effective radiative forcing estimate from each RCM.

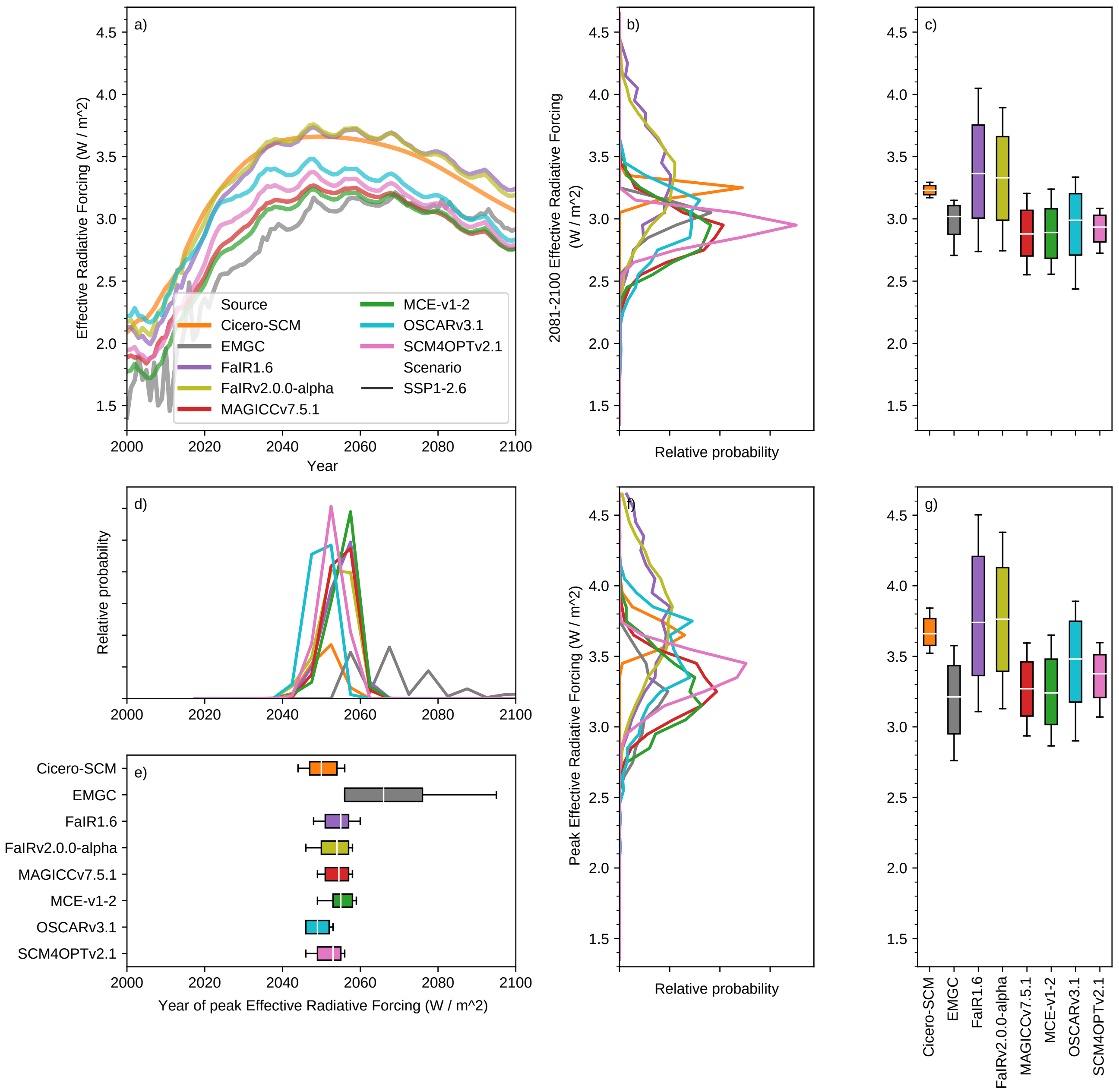

Figure S15. As in Figure 3 except for the low-emissions SSP1-2.6 scenario.

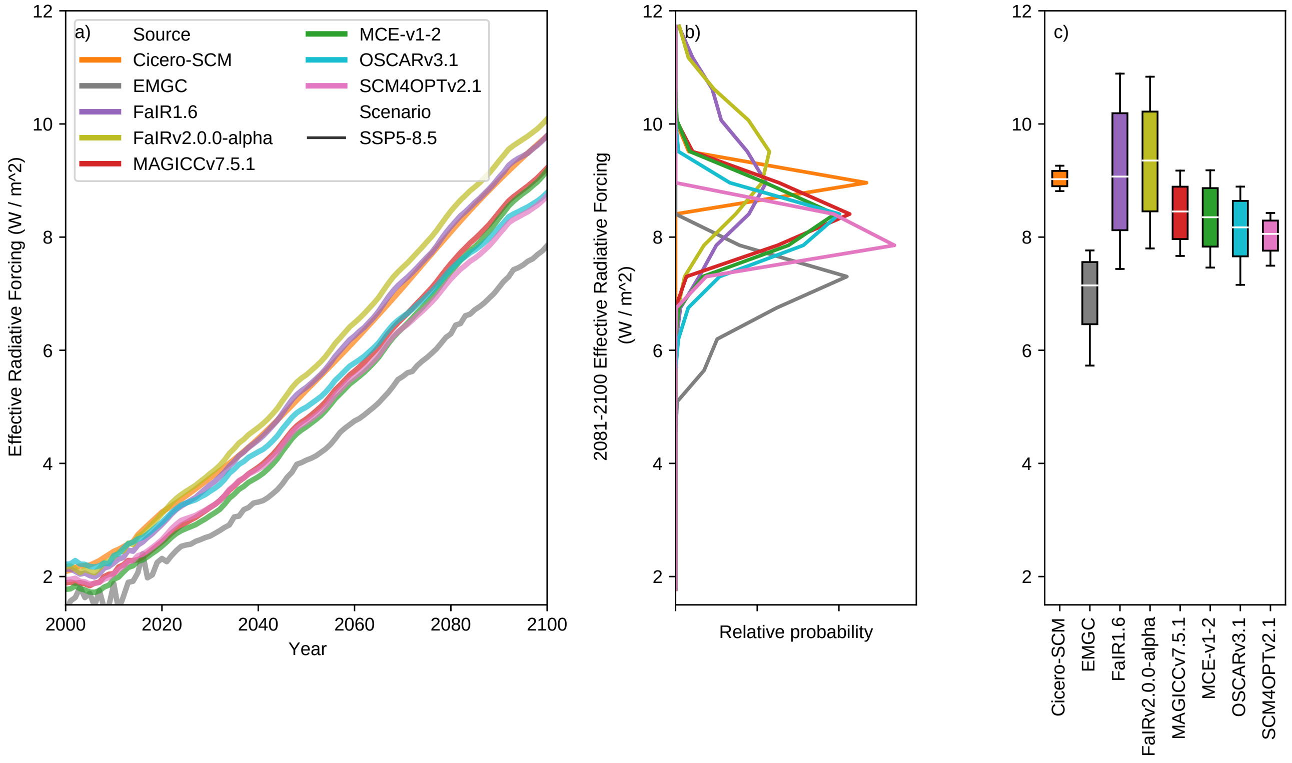

Figure S16. As in panels a), b) and c) of Figure 3 except for the high-emissions SSP5-8.5 scenario.

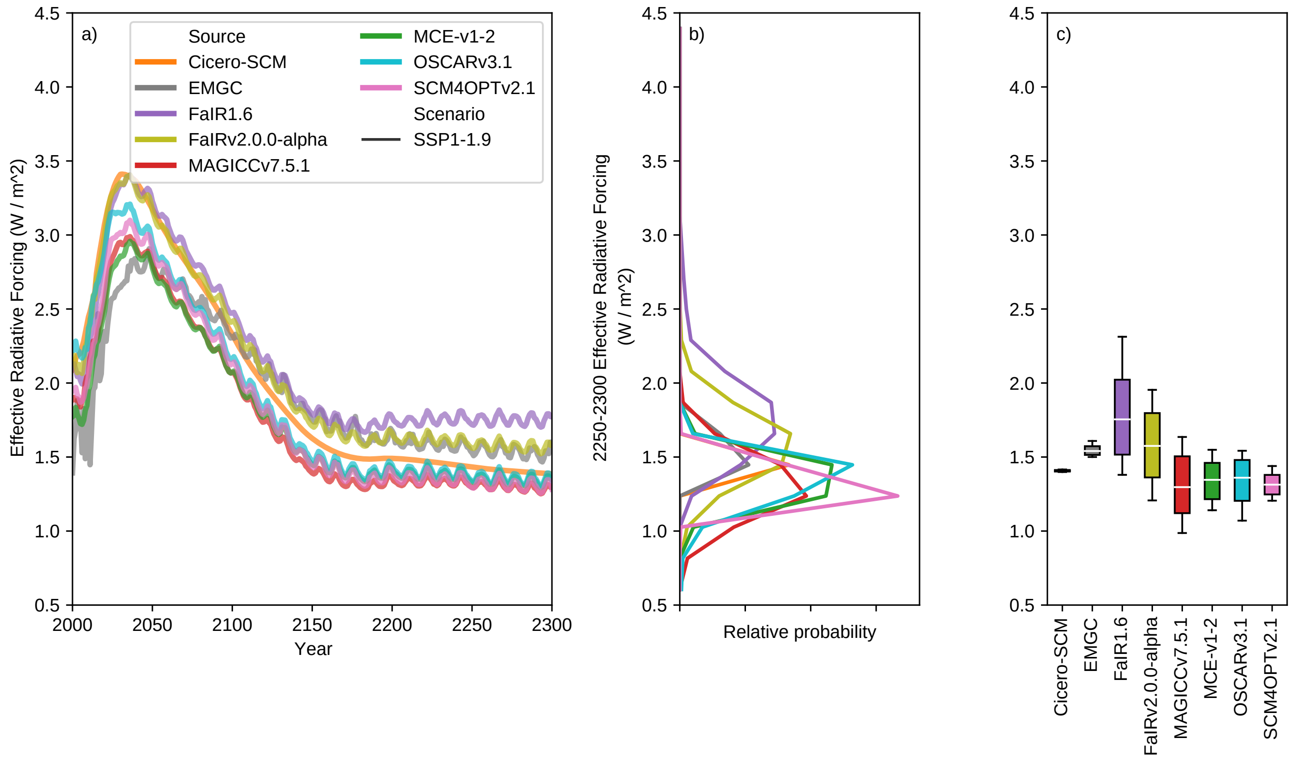

Figure S17. As in Figure S14 except for the low-emissions SSP1-1.9 scenario.

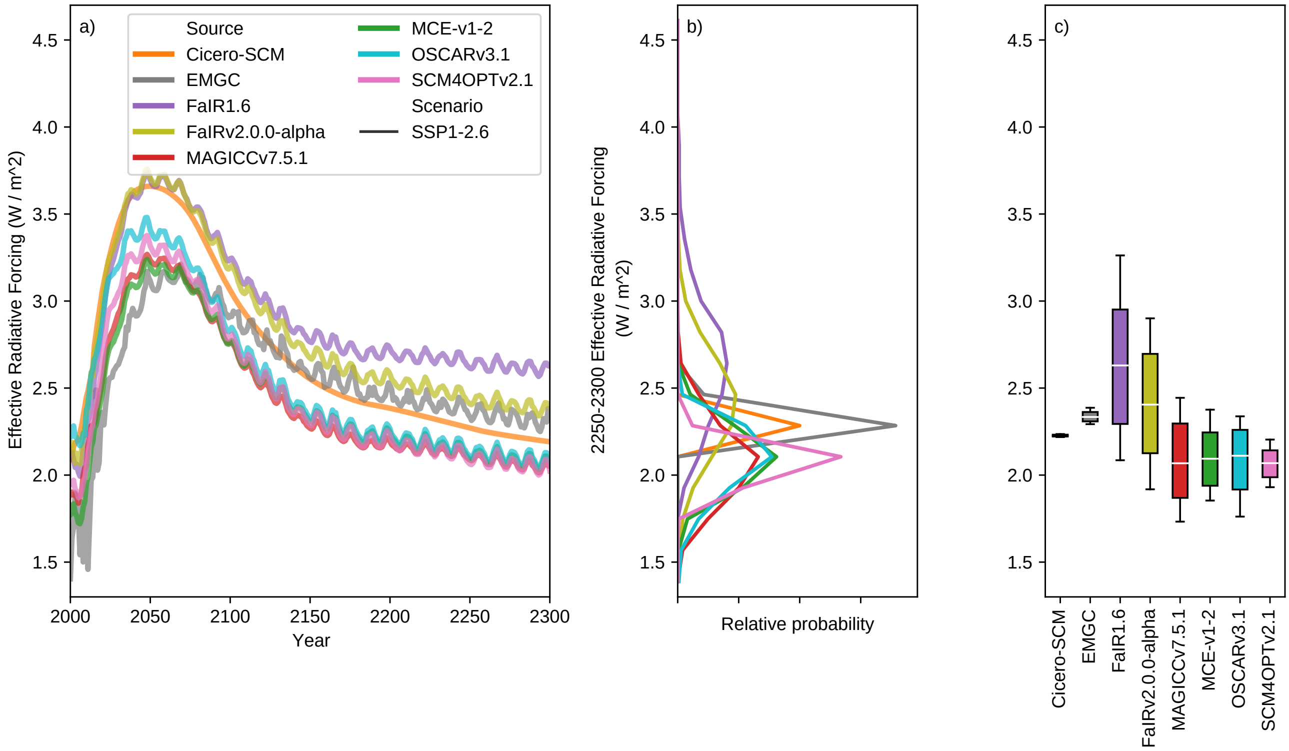

Figure S18. As in Figure S14 except for the low-emissions SSP1-2.6 scenario.

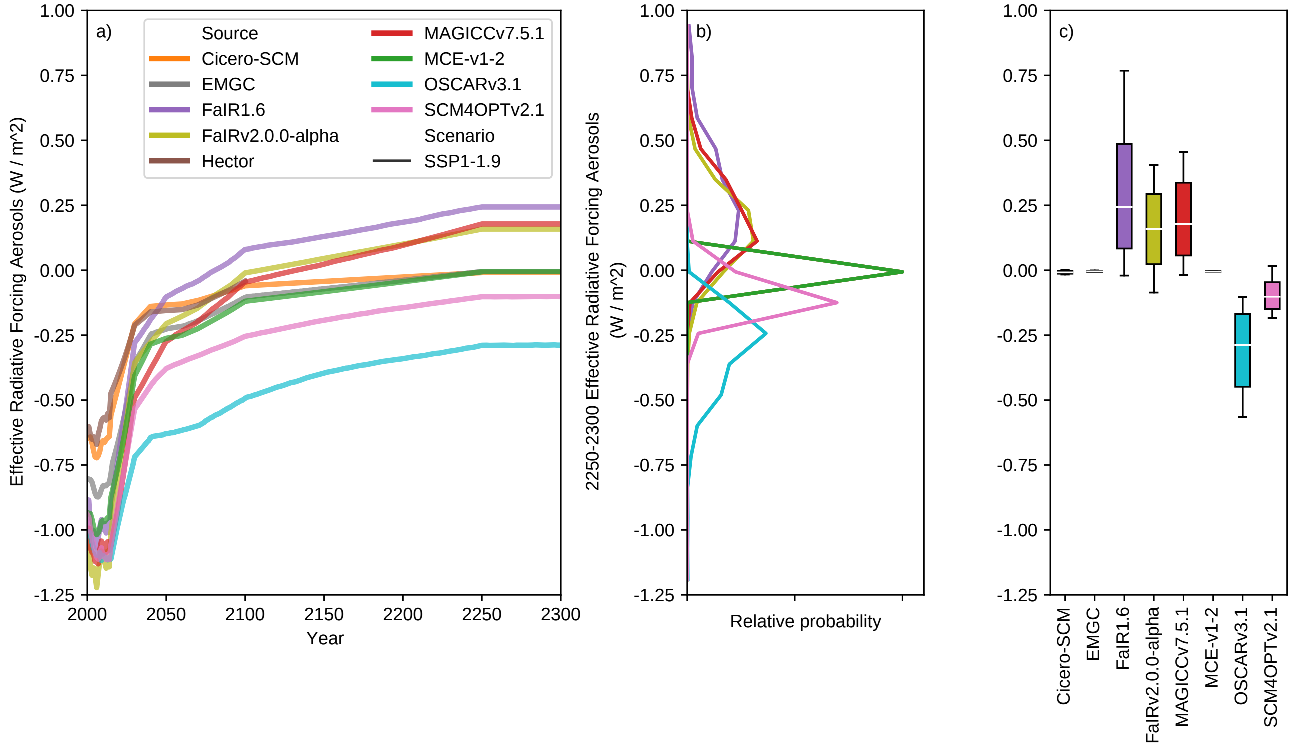

Figure S19. As in Figure S14 except for effective radiative forcing due to aerosols in the low-emissions SSP1-1.9 scenario.

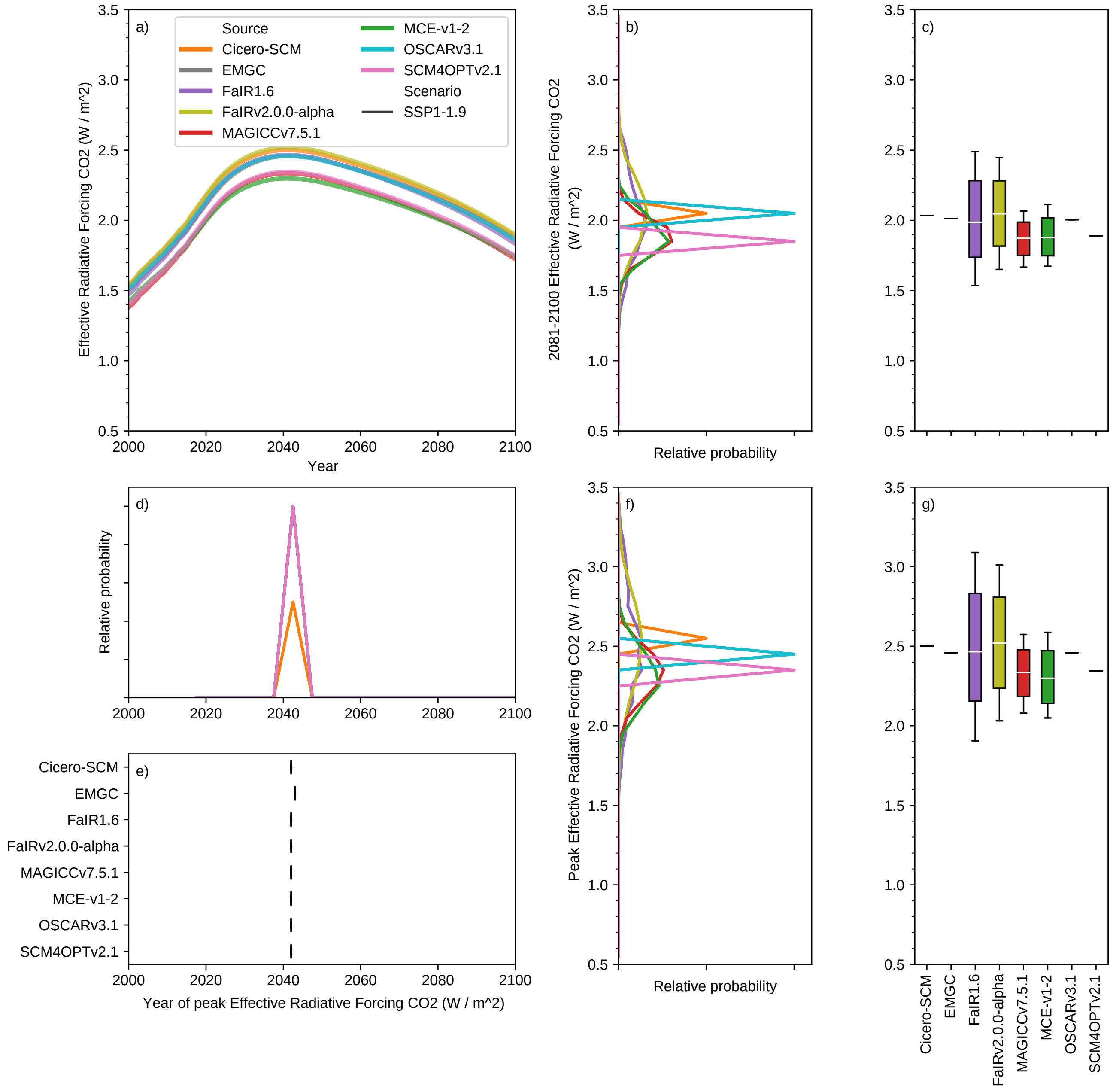

Figure S20. As Figure 3, except for effective radiative forcing due to CO_2.

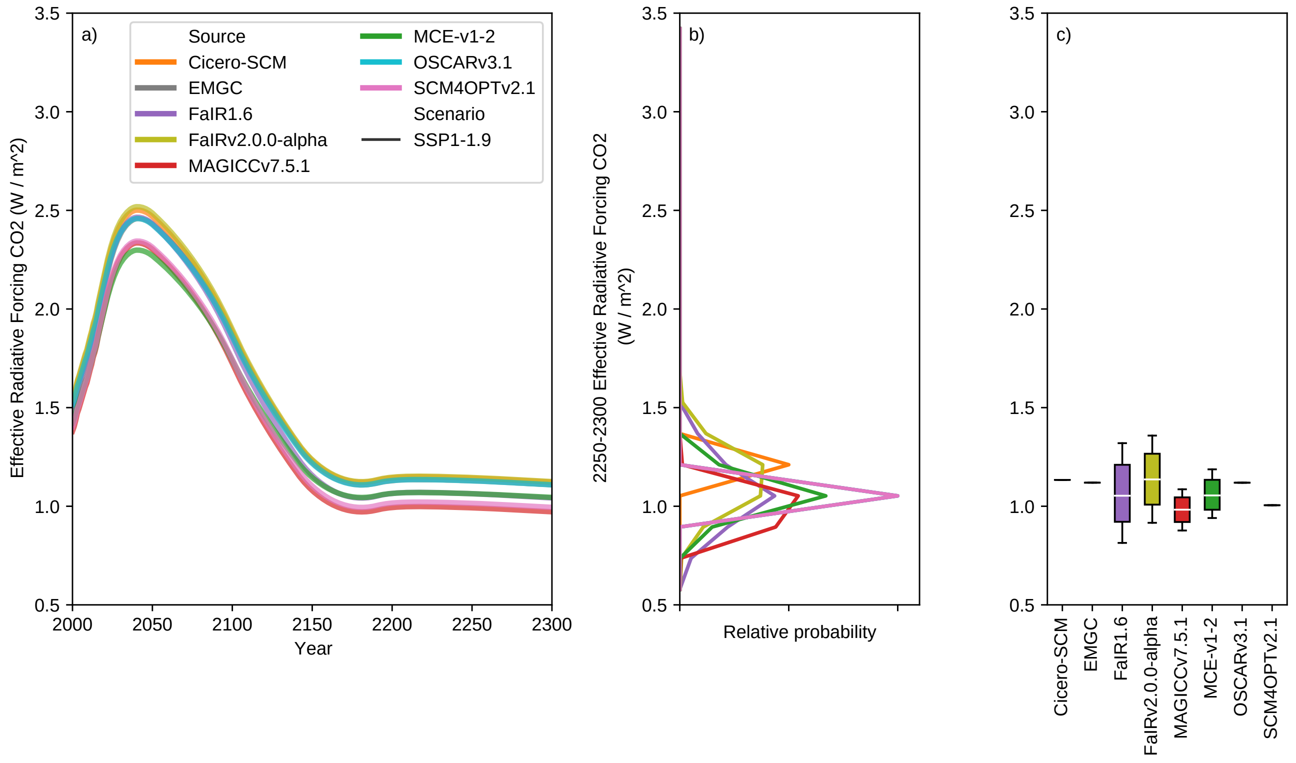

Figure S21. As in Figure S14 except for effective radiative forcing due to CO_2 in the low-emissions SSP1-1.9 scenario.

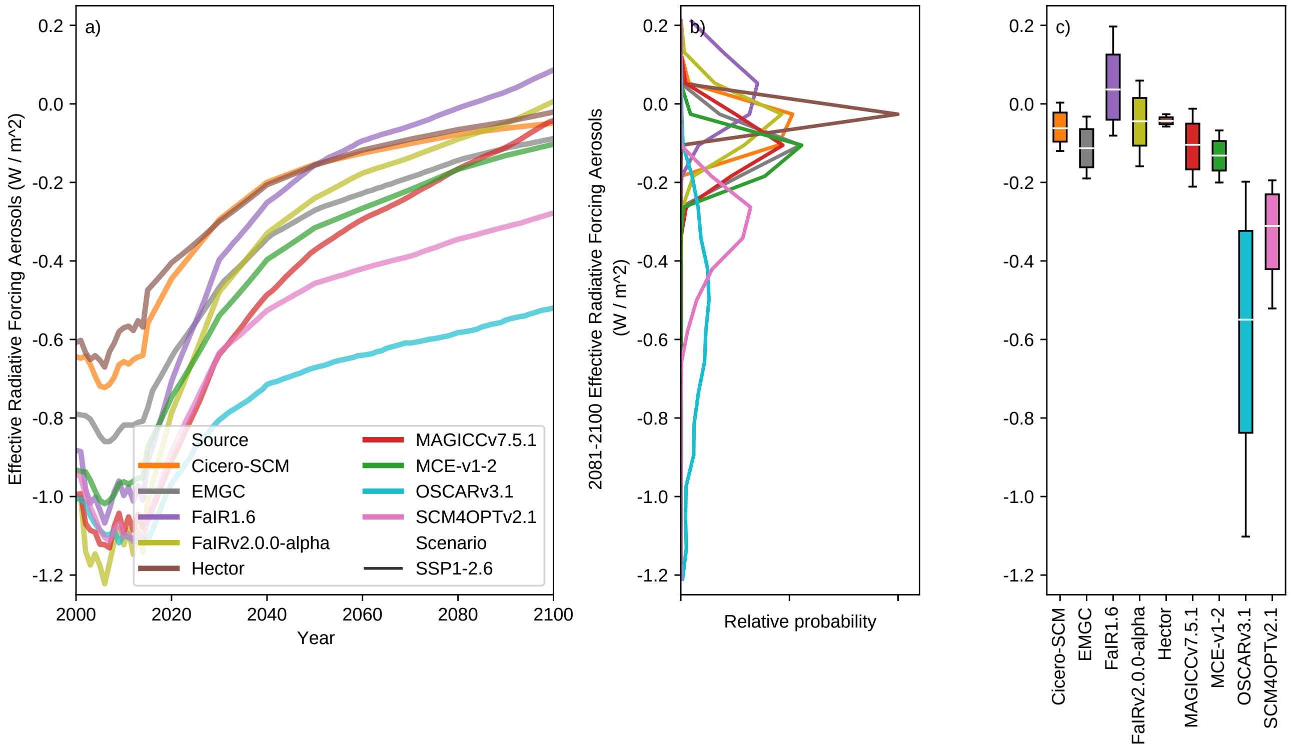

Figure S22. As in panels a), b) and c) of Figure 3, except for effective radiative forcing due to aerosols under the low-emissions SSP1-2.6 scenario.

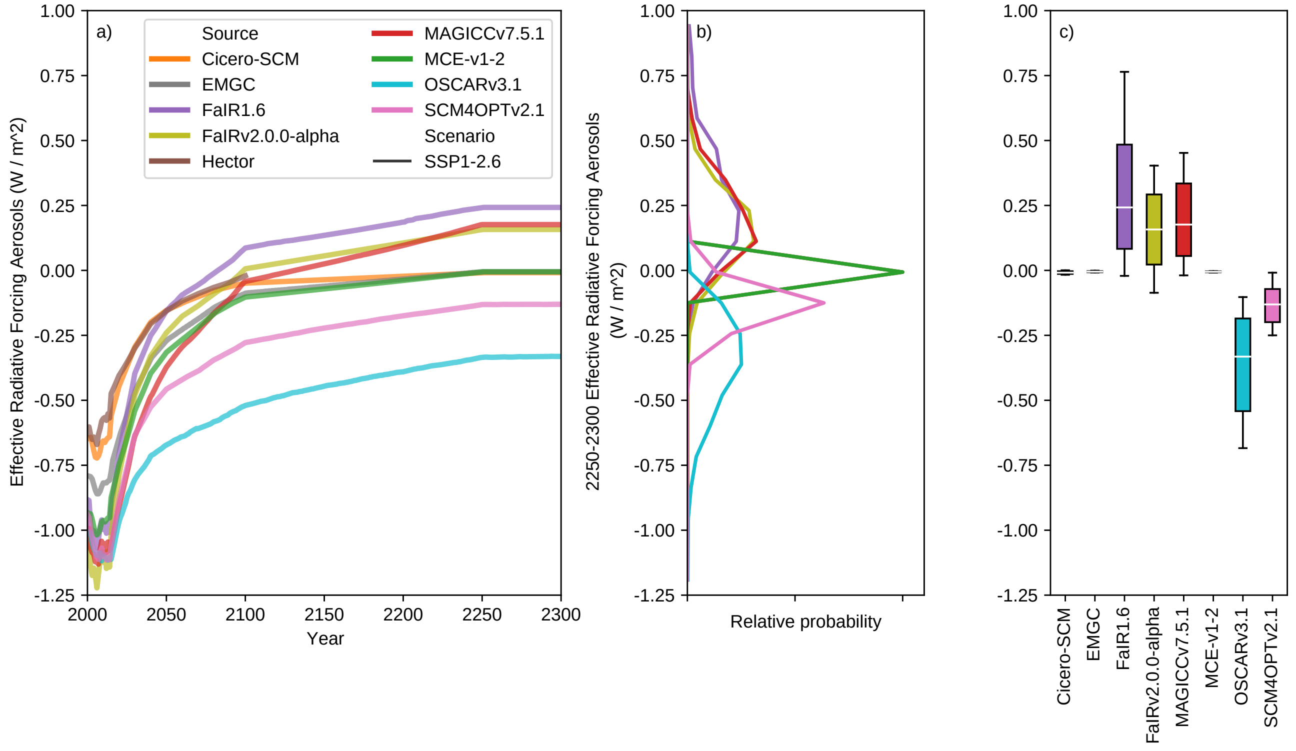

Figure S23. As in Figure S14 except for effective radiative forcing due to aerosols in the low-emissions SSP1-2.6 scenario.

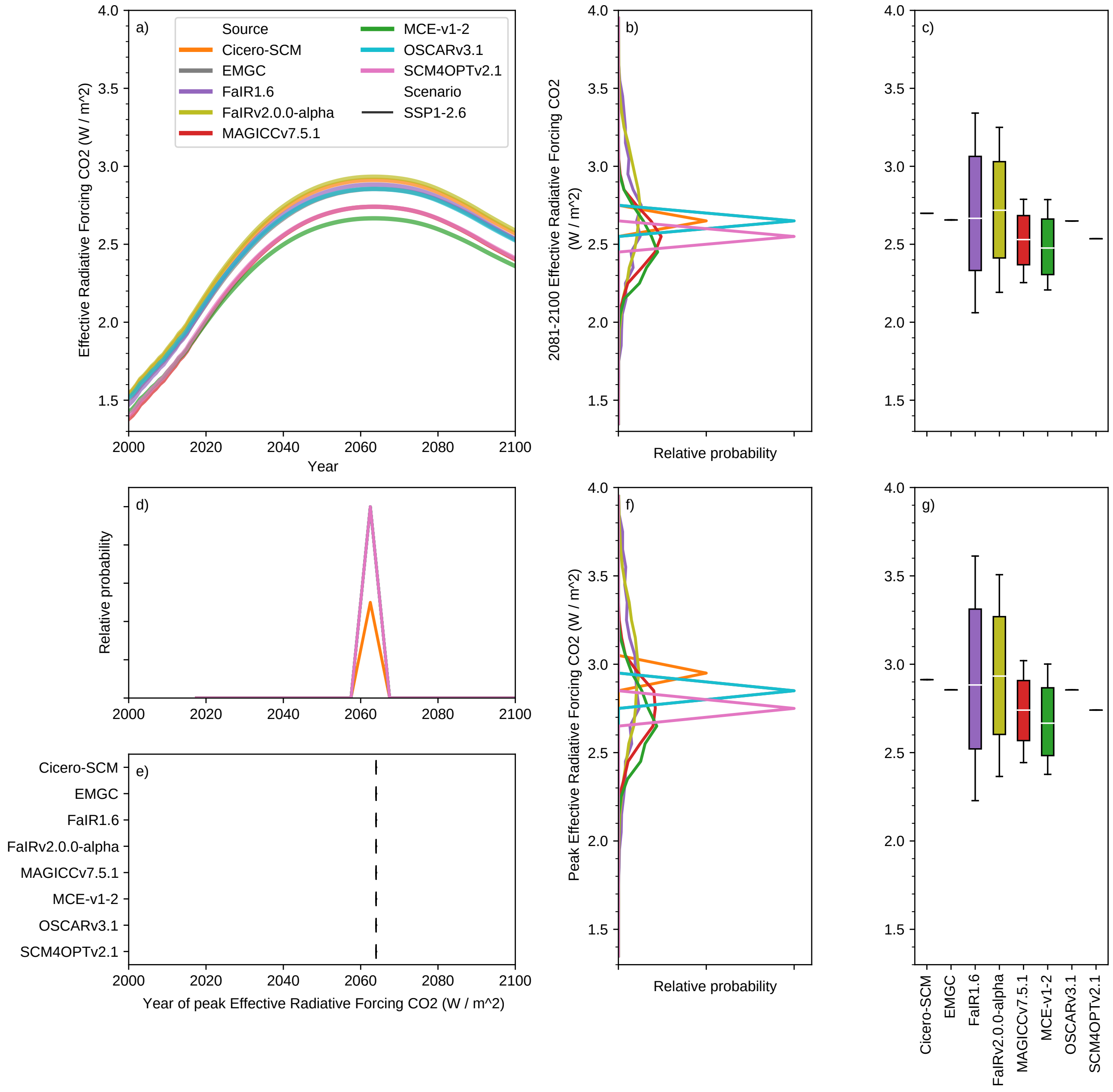

Figure S24. As Figure 3, except for effective radiative forcing due to CO_2 under the low-emissions SSP1-2.6 scenario.

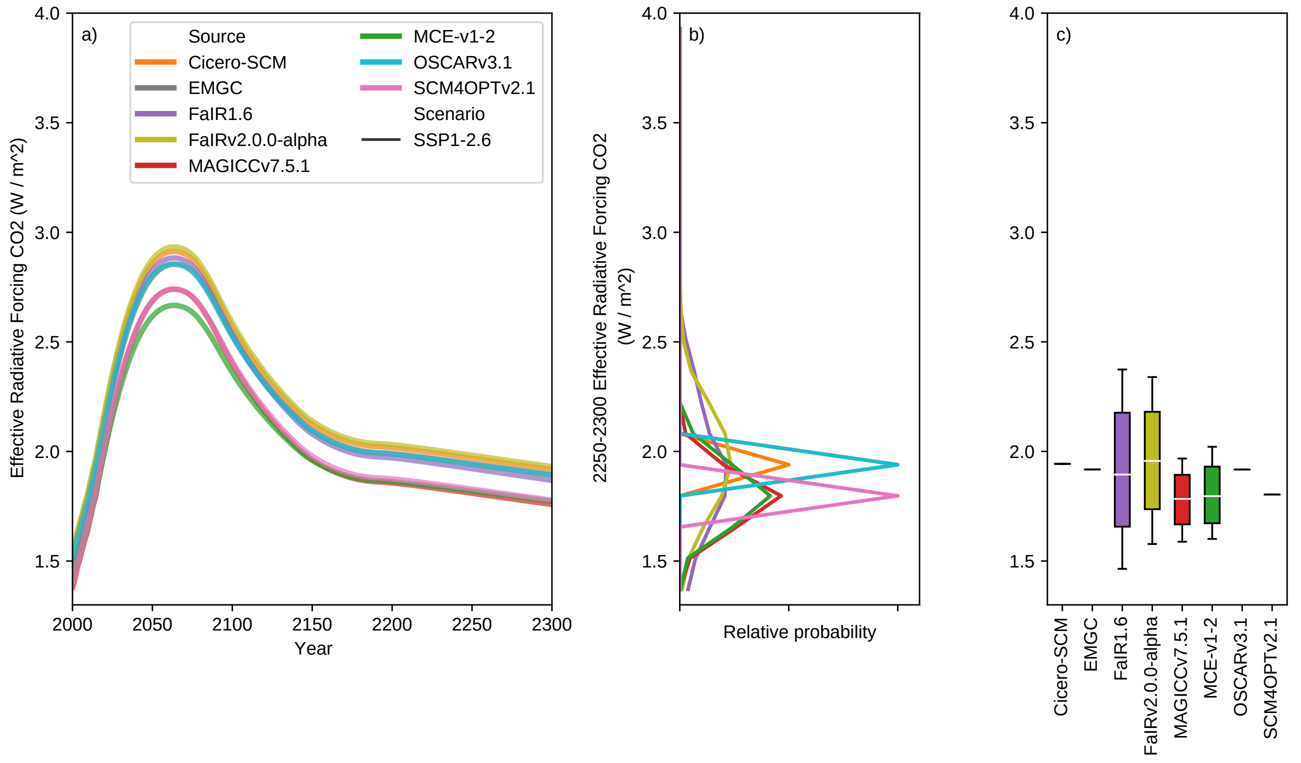

Figure S25. As in Figure S14 except for effective radiative forcing due to CO_2 in the low-emissions SSP1-2.6 scenario.

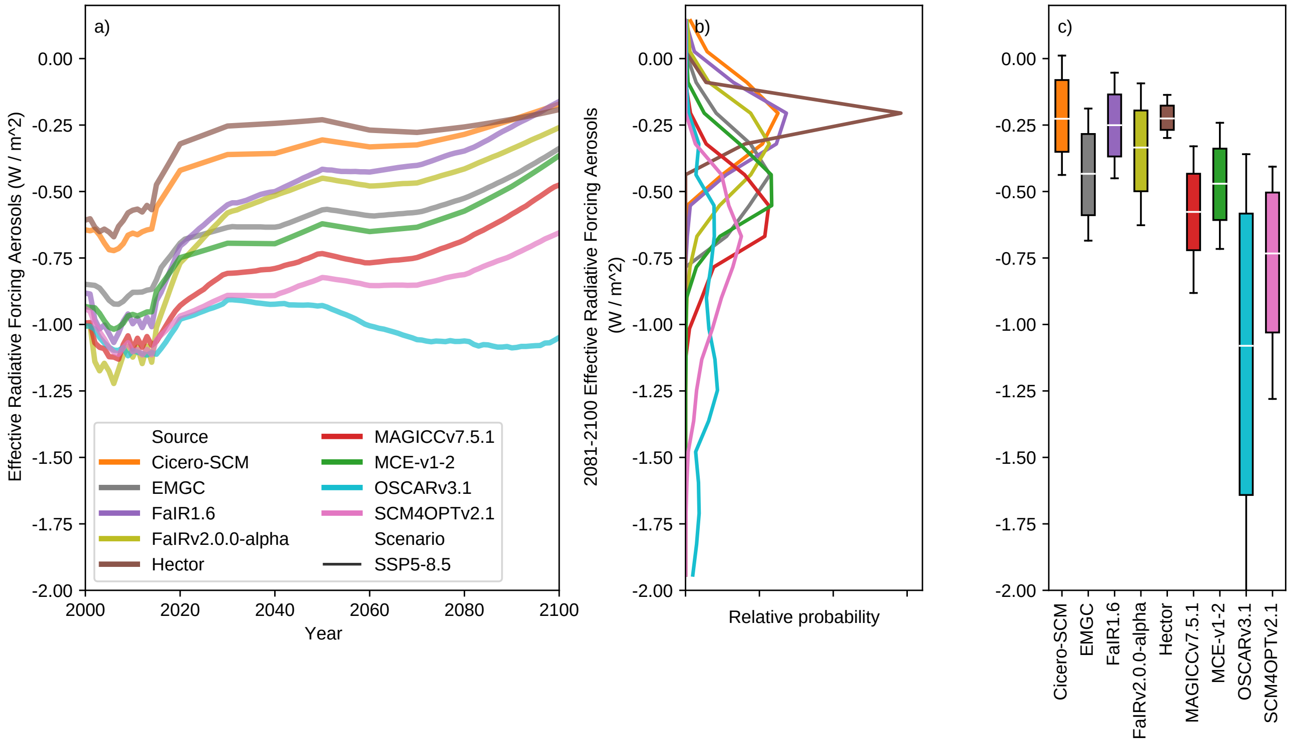

Figure S26. As in panels a), b) and c) of Figure 3, except for effective radiative forcing due to aerosols under the high-emissions SSP5-8.5 scenario.

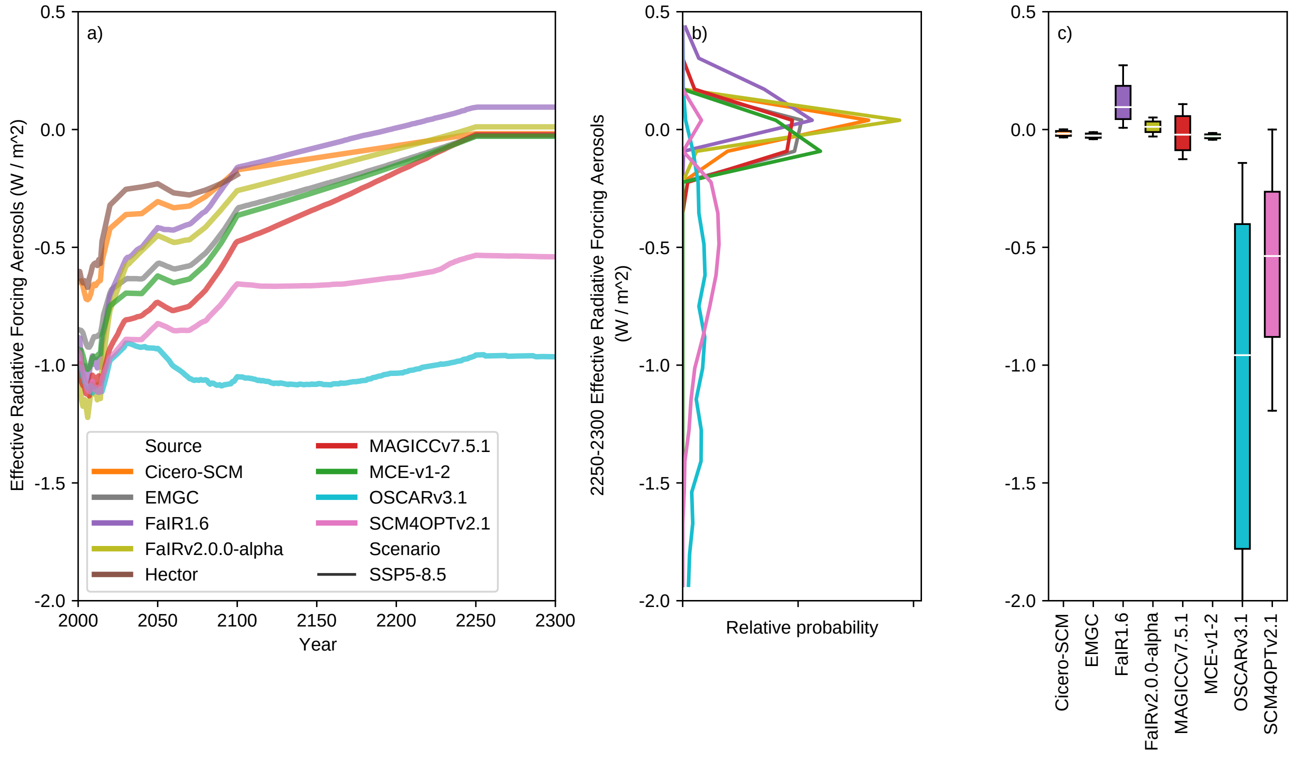

Figure S27. As in Figure S14 except for effective radiative forcing due to aerosols in the high-emissions SSP5-8.5 scenario.

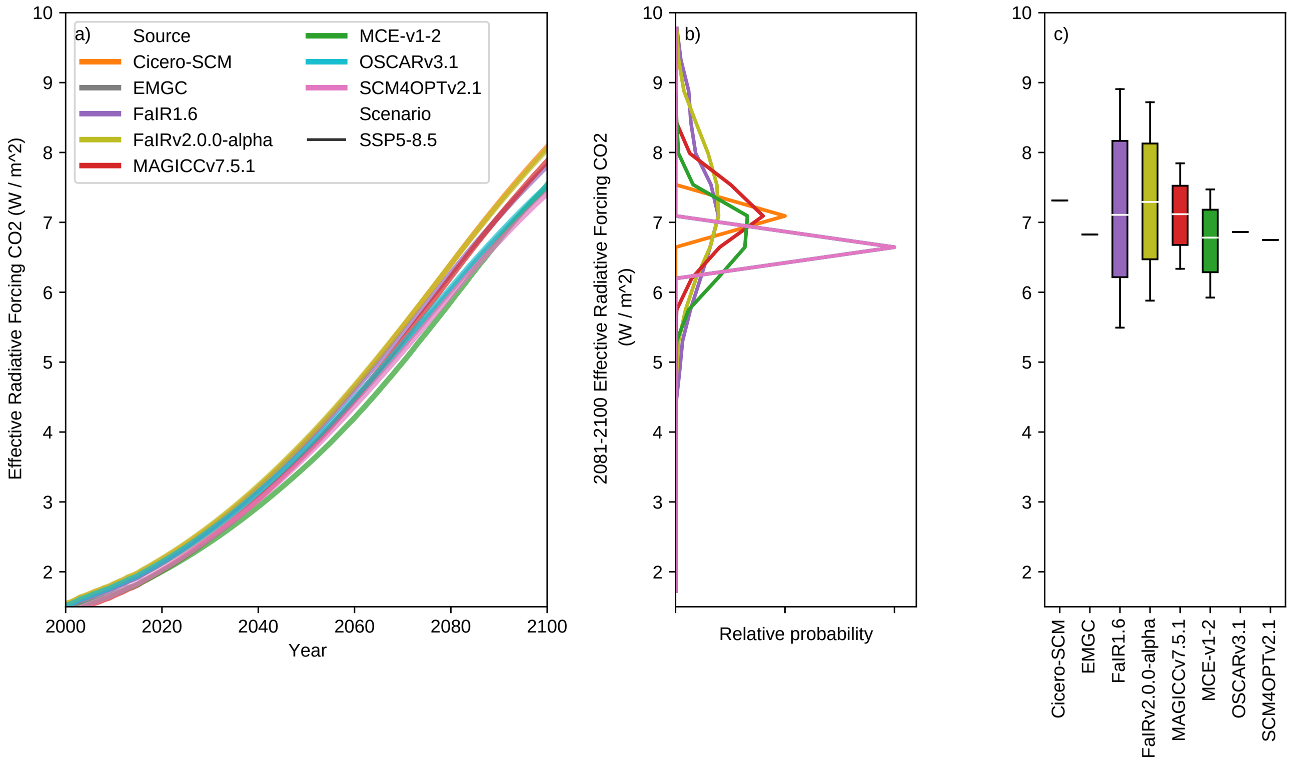

Figure S28. As in panels a), b) and c) of Figure 3, except for effective radiative forcing due to CO_2 under the high-emissions SSP5-8.5 scenario.

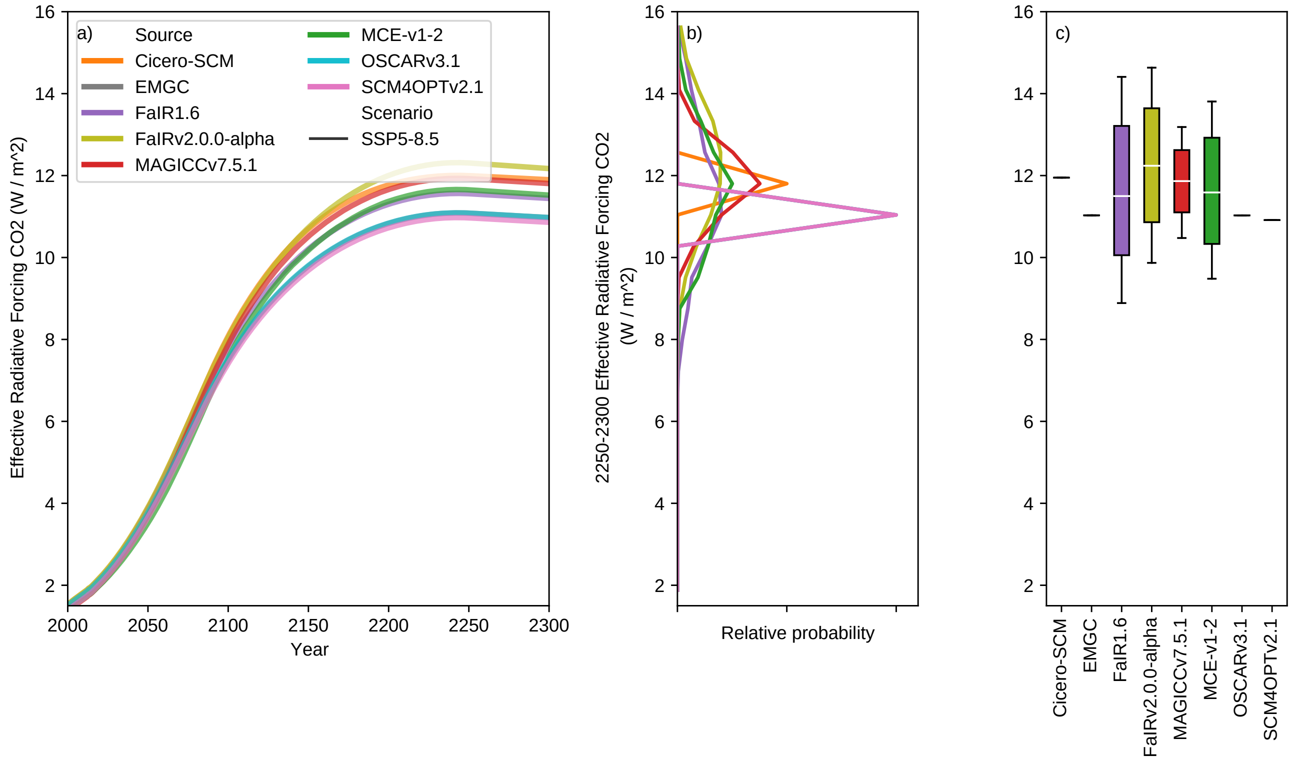

Figure S29. As in Figure S14 except for effective radiative forcing due to CO_2 in the high-emissions SSP5-8.5 scenario.

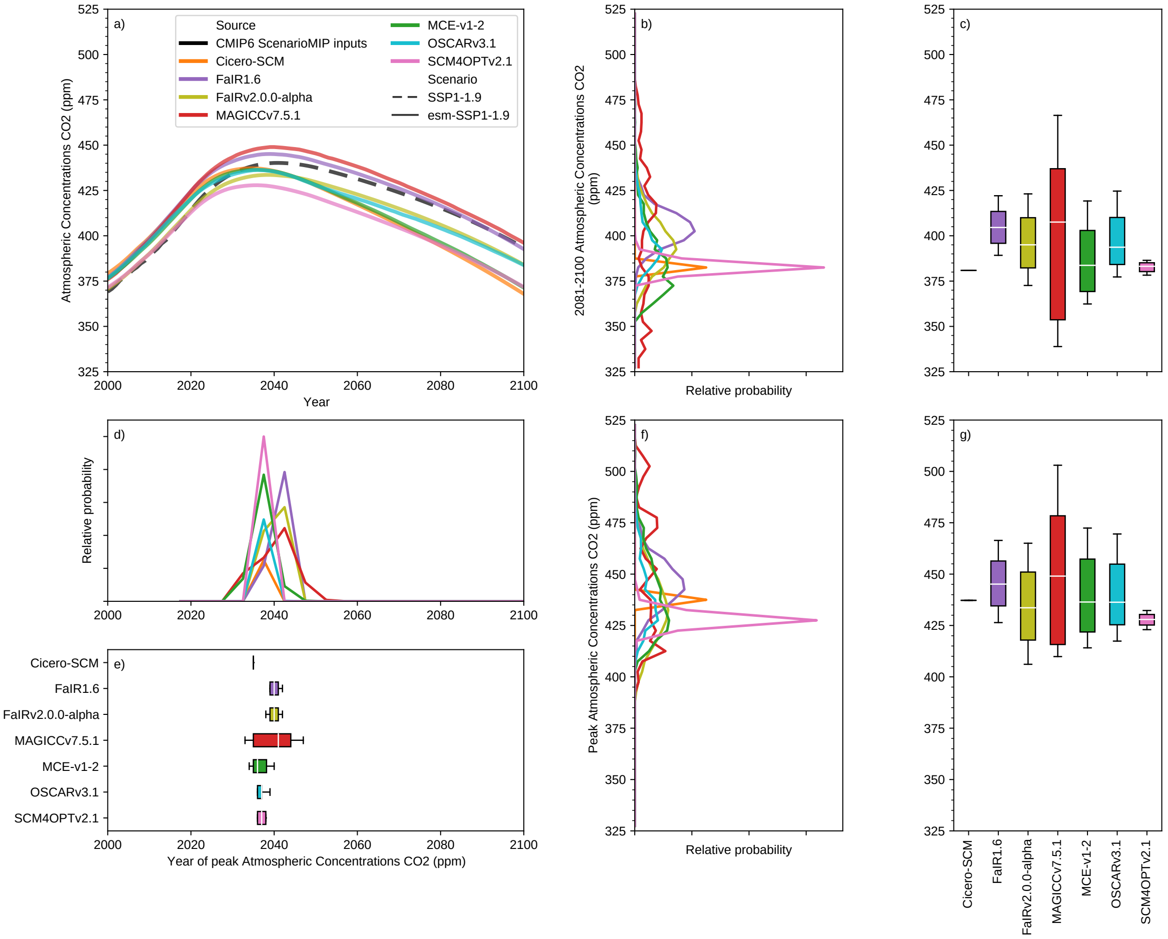

Figure S30. Atmospheric CO_2 concentration projections in the esm-SSP1-1.9 experiment. a) Atmospheric CO_2 concentration projections from 1995 to 2100. We show the median RCM projections (coloured lines) and prescribed CMIP6 ScenarioMIP input concentrations from the SSP1-1.9 concentration-driven experiment (dashed black line); b) distribution of 2081-2100 mean atmospheric CO_2 concentration projections from each RCM; c) very likely (whiskers), likely (box) and central (white line) 2081-2100 mean atmospheric CO_2 concentration projections estimate from each RCM. d) as in b) except for the year in which atmospheric CO_2 concentrations peak; e) as in c) except for the year in which atmospheric CO_2 concentrations peak; f) as in b) except for the peak atmospheric CO_2 concentrations; g) as in c) except for the peak atmospheric CO_2 concentrations. Note that FaIR1.6 data is taken from the esm-SSP1-1.9-allGHG simulations because esm-SSP1-1.9 simulations are not available.

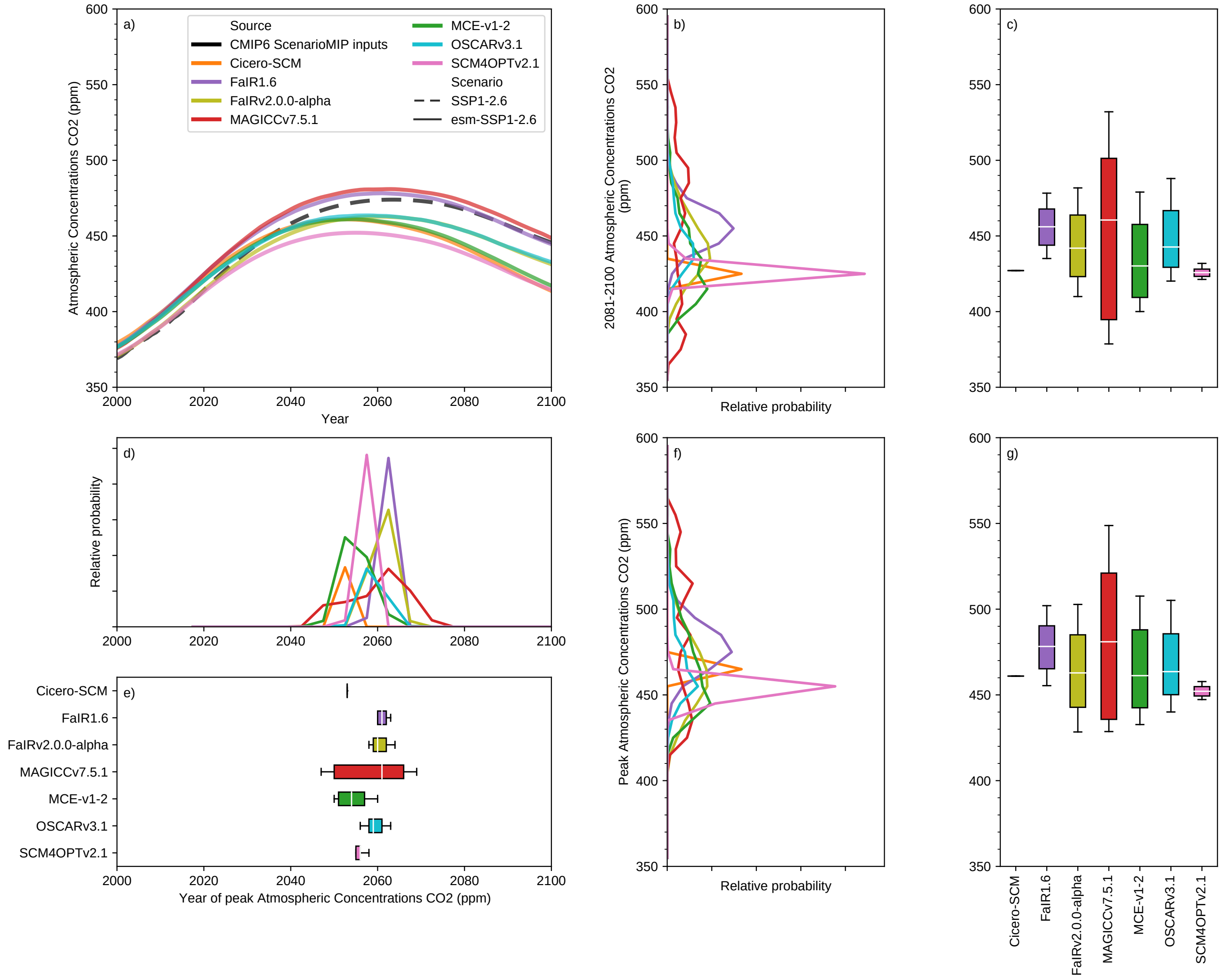

Figure S31. As in Figure S30 except for the esm-SSP1-2.6 experiment. Note that FaIR data is taken from the esm-SSP1-2.6-allGHG simulations because esm-SSP1-2.6 simulations are not available.

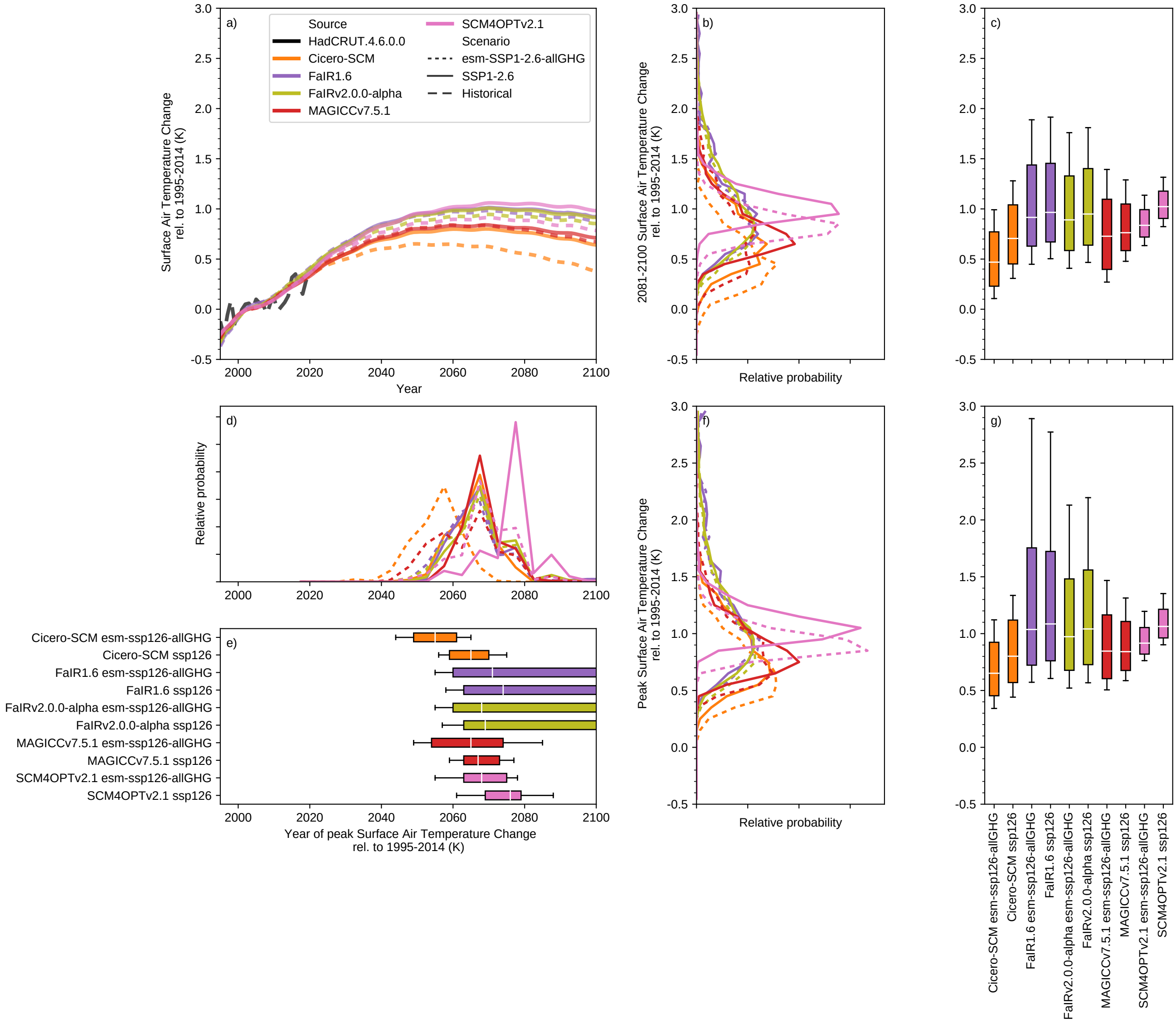

Figure S32. As in Figure 6 except for the SSP1-2.6, esm-SSP1-2.6-allGHG scenario pair.

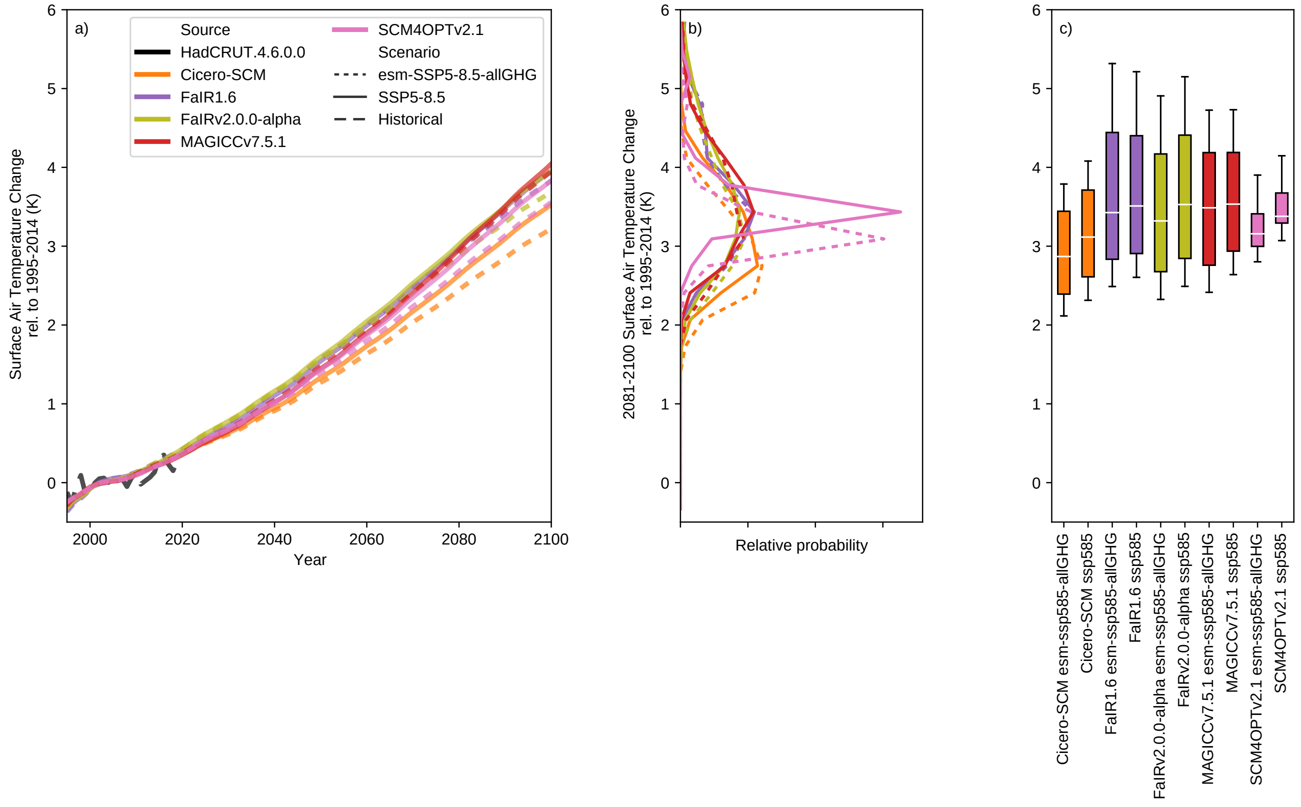

Figure S33. As in panels a), b) and c) of Figure 6 except for the SSP5-8.5, esm-SSP5-8.5-allGHG scenario pair.

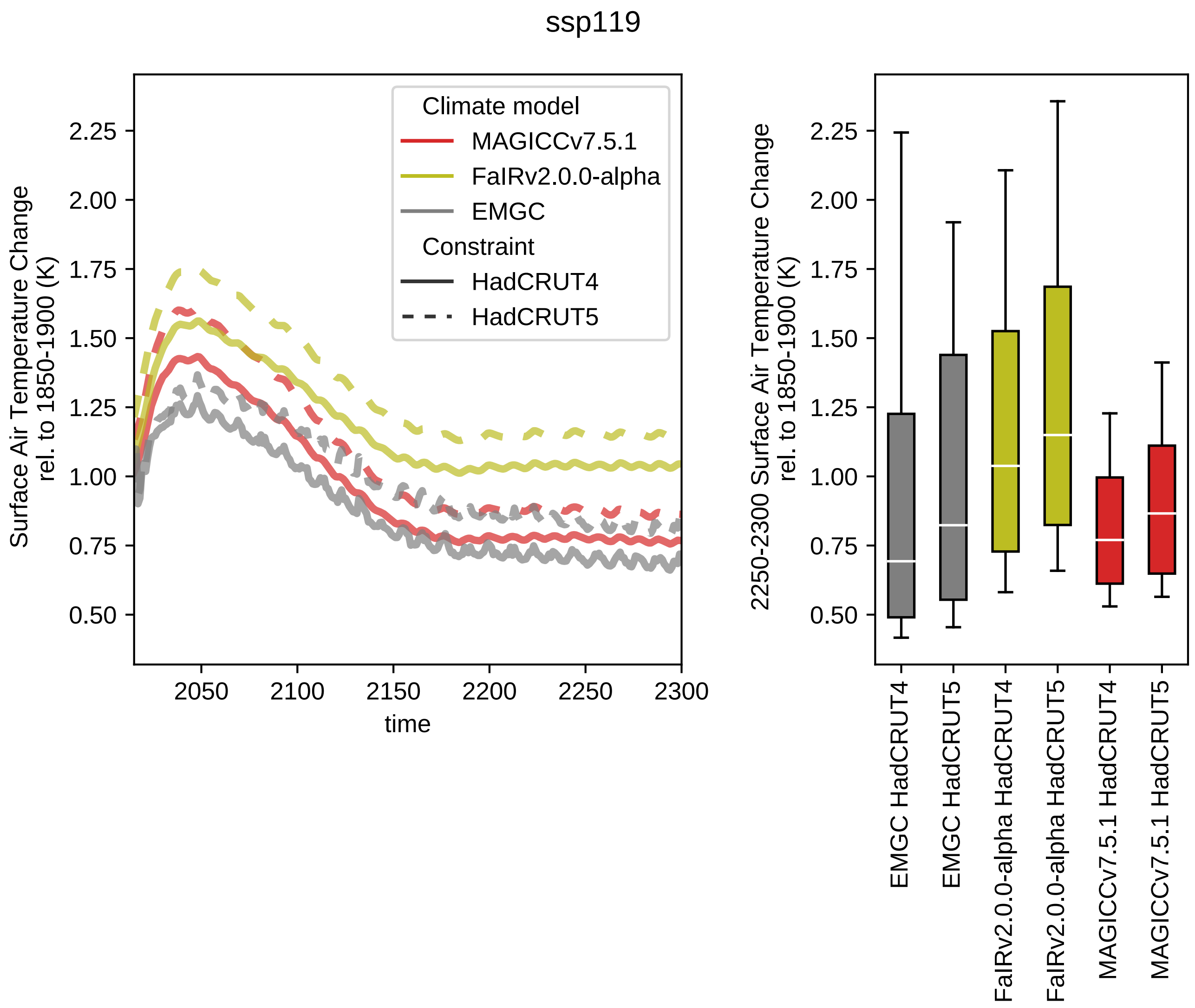

Figure S34. Surface air temperature (also referred to as global-mean surface air temperature, GSAT) change in the concentration-driven SSP1-1.9 experiment. For each model, two different probabilistic distributions are shown. One is constrained to HadCRUT.4.6.0.0 (Morice et al., 2012, as used in the main study), the second is constrained to HadCRUT.5.0.1.0 (Morice et al., 2021), which makes higher estimates of historical-warming. a) GSAT projections from 1995 to 2300. We show the median RCM projections for the probabilistic distributions which used HadCRUT.4.6.0.0 (solid lines) and HadCRUT.5.0.1.0 (dashed lines) as constraints; b) very likely (whiskers), likely (box) and central (white line) 2250-2300 mean GSAT for each RCM for each probabilistic distribution. All results are shown relative to the 1850-1900 reference period.

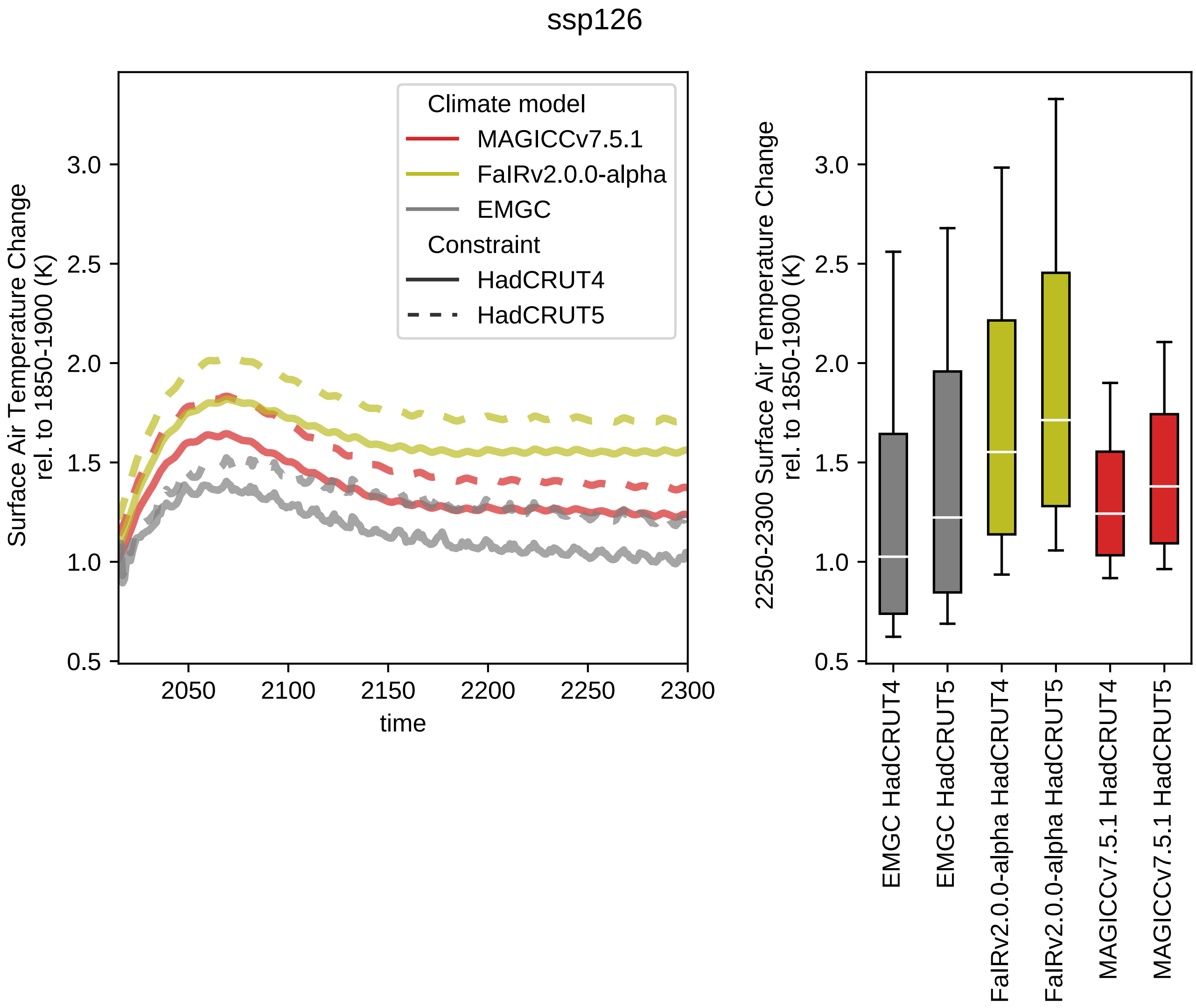

Figure S35. As in Figure S35, except for the concentration-driven SSP1-2.6 experiment.

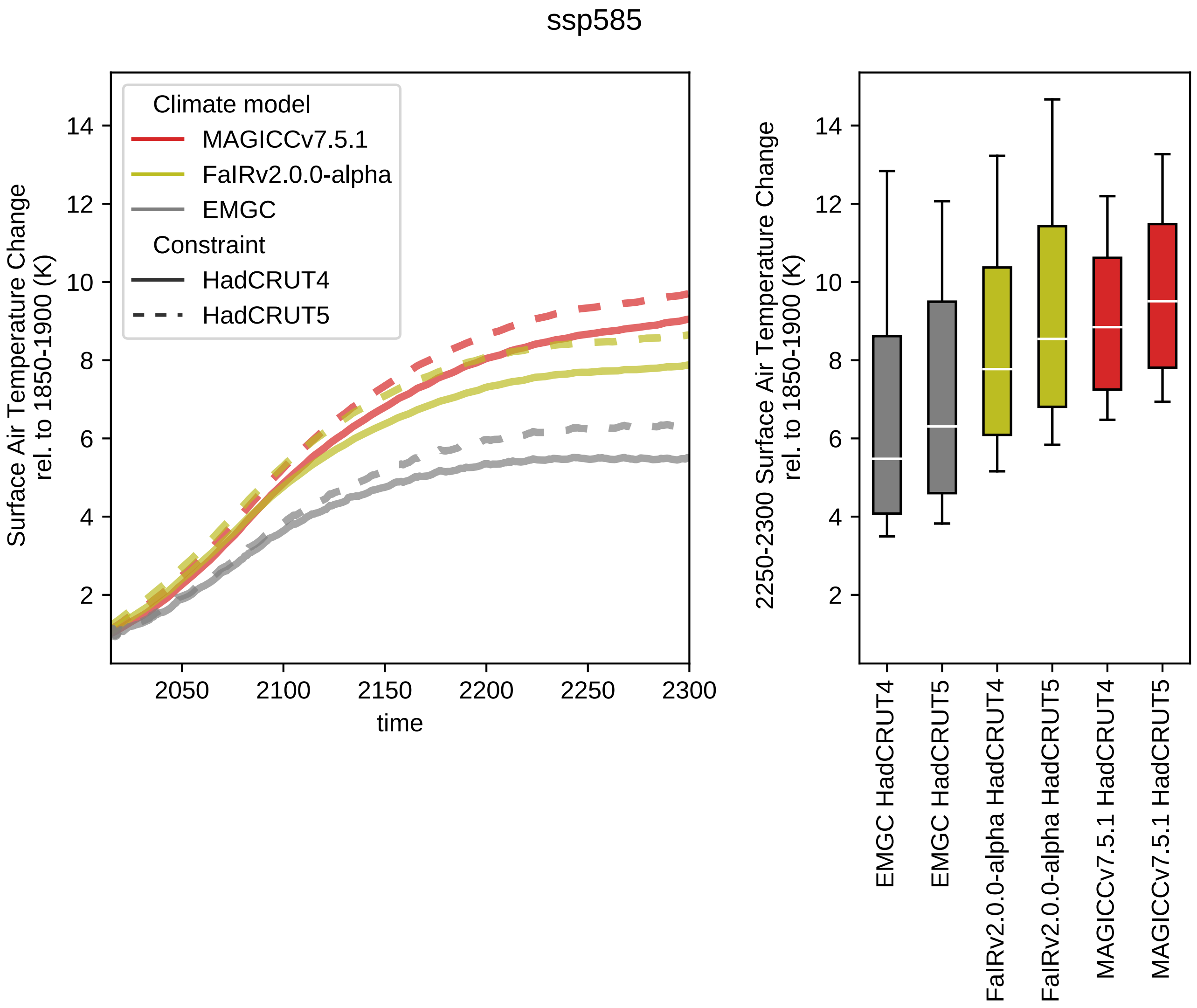

Figure S36. As in Figure S36, except for the concentration-driven SSP5-8.5 experiment.
